# Development of the Pyrido[2,3-*d*]pyrimidin-7(8*H*)-one Scaffold toward Potent and Selective NUAK1 Inhibitors

**DOI:** 10.1021/acsmedchemlett.4c00579

**Published:** 2025-01-25

**Authors:** Timothy P. C. Rooney, Gregory G. Aldred, David Winpenny, Helen Scott, Henriette M. G. Willems, Iryna Voytyuk, Jonathan H. Clarke, Helen K. Boffey, Stephen P. Andrews, John Skidmore

**Affiliations:** The ALBORADA Drug Discovery Institute, University of Cambridge, Cambridge CB2 0AH, U.K.

**Keywords:** NUAK1, Kinase selectivity, ADME properties, In vivo profile

## Abstract

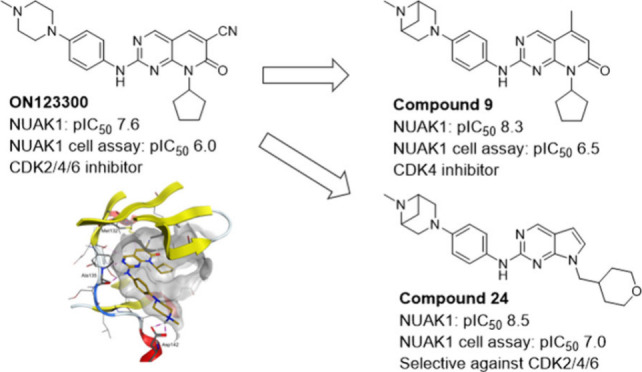

The protein kinase NUAK1 has been implicated in various biological functions including cell adhesion, migration and proliferation. Genetic reduction of NUAK1 expression has notably been shown to lower total levels of human tau in a tauopathy mouse model, identifying this kinase as a potential therapeutic target for neurodegenerative disease. In this paper, we describe improvement of the NUAK1 potency, kinase-selectivity and pharmacokinetic properties of the brain-penetrant but unselective CDK4/CDK6/NUAK1 inhibitor ON123300. Through a scaffold-optimization approach we have identified different chemotypes delivering NUAK1 inhibition with improved potency and selectivity over CDK kinases compared with ON123300. We present ADME profiling and in vivo pharmacokinetic data for these compounds.

The NUAK1 family SNF1-like kinase 1 (NUAK1, also known as AMPK-related protein kinase 5 or ARK5) is related to the cellular AMP:ATP sensor AMP-activated protein kinase (AMPK). Under conditions of metabolic stress, AMPK is activated by liver kinase B1 (LKB1, STK11), which leads to the regulation of diverse cell survival mechanisms.^1^ The family of 12 AMPK-related serine/threonine kinases (ARKs), of which NUAK1 is a member, is defined by amino acid sequence homology to the catalytic subunit of AMPK, and the majority of these kinases are also activated by LKB1 phosphorylation of a threonine residue in the conserved T-loop structure.^2^ Unlike AMPK itself, the ARK proteins are activated independently of cellular ATP levels and thus regulate cellular homeostasis indirectly.^3^ NUAK1 is attributed to a broad range of functions such as cell adhesion, migration and proliferation.^4^ Despite canonical activation by the tumor suppressor kinase LKB1, the role of NUAK1 in these cellular functions has identified this kinase as a target involved in tumor survival and promotion of metastasis.^5,6^ Evidence has shown NUAK1 is a survival factor in MYC-driven tumors,^7^ promotes tumor progression^8,9^ and is upregulated in a number of tumor types.^10,11^ NUAK1 also has a well-established role in embryonic development, causing neural tube closure defects^12,13^ and has a role in neuronal shaping and branching.^14,15^ Subsequent research has implicated NUAK1 in neuro-developmental disorders and neurodegenerative disease.^16^ Genetic mutation or haploinsufficiency of NUAK1 has been observed in autism spectrum disorders and neurodevelopmental cognitive impairment.^17−19^ Tauopathies such as Alzheimer’s disease (AD) and progressive supranuclear palsy (PSP) show characteristic accumulation of aggregated, hyperphosphorylated tau protein,^20^ and NUAK1 has been shown to associate with these aggregates in human post-mortem brain from AD patients and to directly phosphorylate tau.^21^ Genetic reduction of NUAK1 activity was able to lower total levels of human tau in a tauopathy mouse model, suggesting this kinase as a therapeutic target for neurodegenerative disease.^21^

The deregulation of NUAK1 activity, usually involving overexpression of endogenous protein levels, in both cancer and in neurodevelopmental disorders and neurodegenerative disease has encouraged the development of inhibitors for this kinase.^6,16^

The chemical probes portal^22^ identifies WZ4003 (**1**) and HTH-01-015 (**2**) as selective NUAK1/NUAK2 inhibitors^23^ (see Figure 1). These tool compounds have been used extensively and enabled further research into NUAK1 mechanisms of action.^10,21,24−26^ In our hands, while these compounds are moderately potent toward NUAK1, they demonstrated low target engagement in a cell-based NanoBRET assay.^27^ In addition, no in vivo data is publicly available for these compounds.

**Figure 1:**
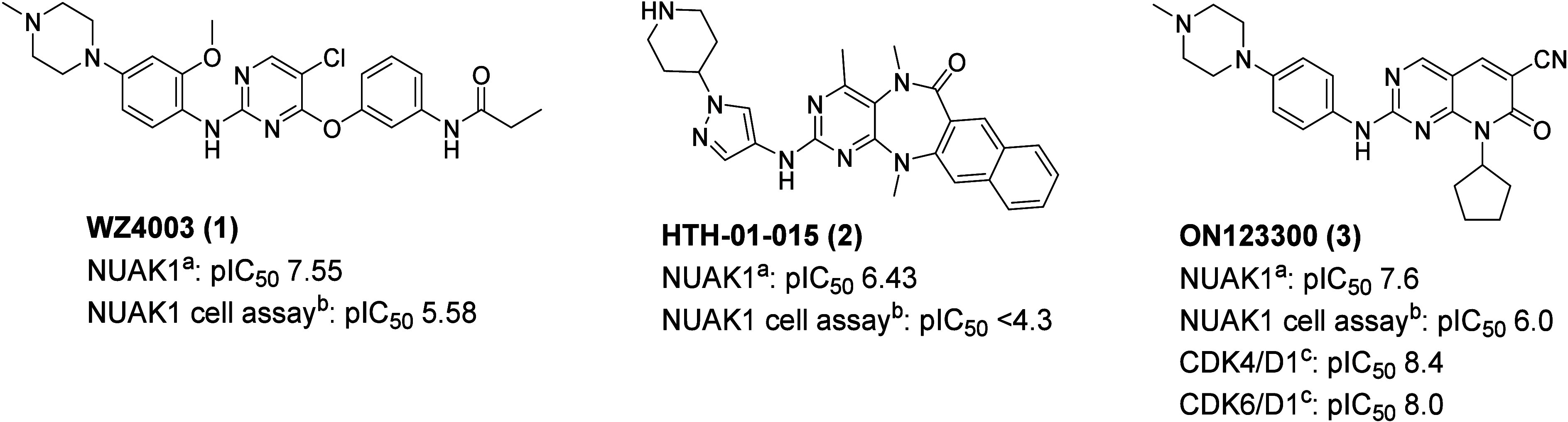
NUAK1 inhibitors WZ4003 (**1**), HTH-01-015 (**2**) and ON123300 (**3**). ^a^Promega ADP-Glo kinase assay, ^b^cell-based NanoBRET target engagement assay, ^c^data from Reddy et al.^28^

Additional NUAK1 and NUAK2 inhibitors have been reported in the literature since the start of our work in this area^24,29−32^ including those summarized in a review of NUAK inhibitors.^6^ However, many of these compounds do not have NUAK1 or NUAK2 as their primary target and no associated ADME or in vivo PK data are available, limiting their value as chemical probes. A report describing a novel class of brain penetrant NUAK1 inhibitors from a high-throughput screening campaign was very recently published,^33^ complementing the approach described herein.

ON123300 (Narazaciclib, **3**) is a dual CDK4/6 and NUAK1 inhibitor^28^ currently in clinical trials for patients with advanced solid tumors (see Figure 1). This compound has a relatively short plasma half-life in mice (I.V. dosing: 5 mgkg^–1^, *t*_1/2_ 1.5 h)^34^ and is not selective for NUAK1 but demonstrates significant brain penetration, making **3** a promising starting point to develop as a selective NUAK1 tool compound for CNS disorders. In this paper, we describe our efforts to optimize this compound by increasing cellular NUAK1 potency, selectivity and metabolic stability to deliver in vivo tools for NUAK1 inhibition.

During the development of NUAK1 inhibitors, we chose MARK3 as a relevant target to screen for selectivity. MARK3 is from the same AMPK-related protein kinase family as NUAK1 (49.4% human kinase-domain homology) and has also been shown to phosphorylate tau.^35^ Additionally, there is evidence that MARK3 inhibition can lead to undesirable effects on blood pressure in rats.^36^

To assess selectivity more broadly, we also screened selected compounds against NUAK2 (82.1% human kinase domain homology with NUAK1^16^) and additional kinases from the AMPK family (MARK1, MARK2, MARK4 and AMPKA). As our compounds were derived from a CDK inhibitor, we also screened compounds against CDK2, CDK4 and CDK6 kinases.

In our hands, **3** is a potent NUAK1 inhibitor in a cell-free biochemical assay with modest cellular target engagement (pIC_50_ values of 7.6 and 6.0 respectively) but excellent selectivity over MARK3 (biochemical pIC_50_ 5.8). However, the rodent metabolic stability of this compound is poor (mouse liver microsomes (MLM) *t*_1/2_ 8.0 min). Our aims were to improve cell potency and increase the metabolic half-life of this series, while maintaining good brain penetration.

There is no published crystal structure of NUAK1 currently available. An analysis of crystal structures in the PDB at the start of the project revealed that there were six protein–ligand complexes with >40% homology to the NUAK1 kinase domain, where the ligand was not a nucleotide. Two of these complexes contain inhibitors with reported NUAK1 activity^37,38^ (pdb: 5EAK and 5KZ8). As both complexes are MARK2 kinases, a NUAK1 homology model was built based on the crystal structure of MARK2 (see Figure 2 and SI for details). The ligand **3** was docked to the ATP site of the model, which suggests that the NH and N(3) of the amino-pyrimidine are involved in hinge binding to Ala135. The nitrile at C(6) occupies a hydrophobic pocket defined by Met132, Lys84 and Ile116, and does not appear to be making any hydrogen bonding interactions. The piperazine substituent extends toward solvent, potentially picking up an interaction with Asp142.

**Figure 2:**
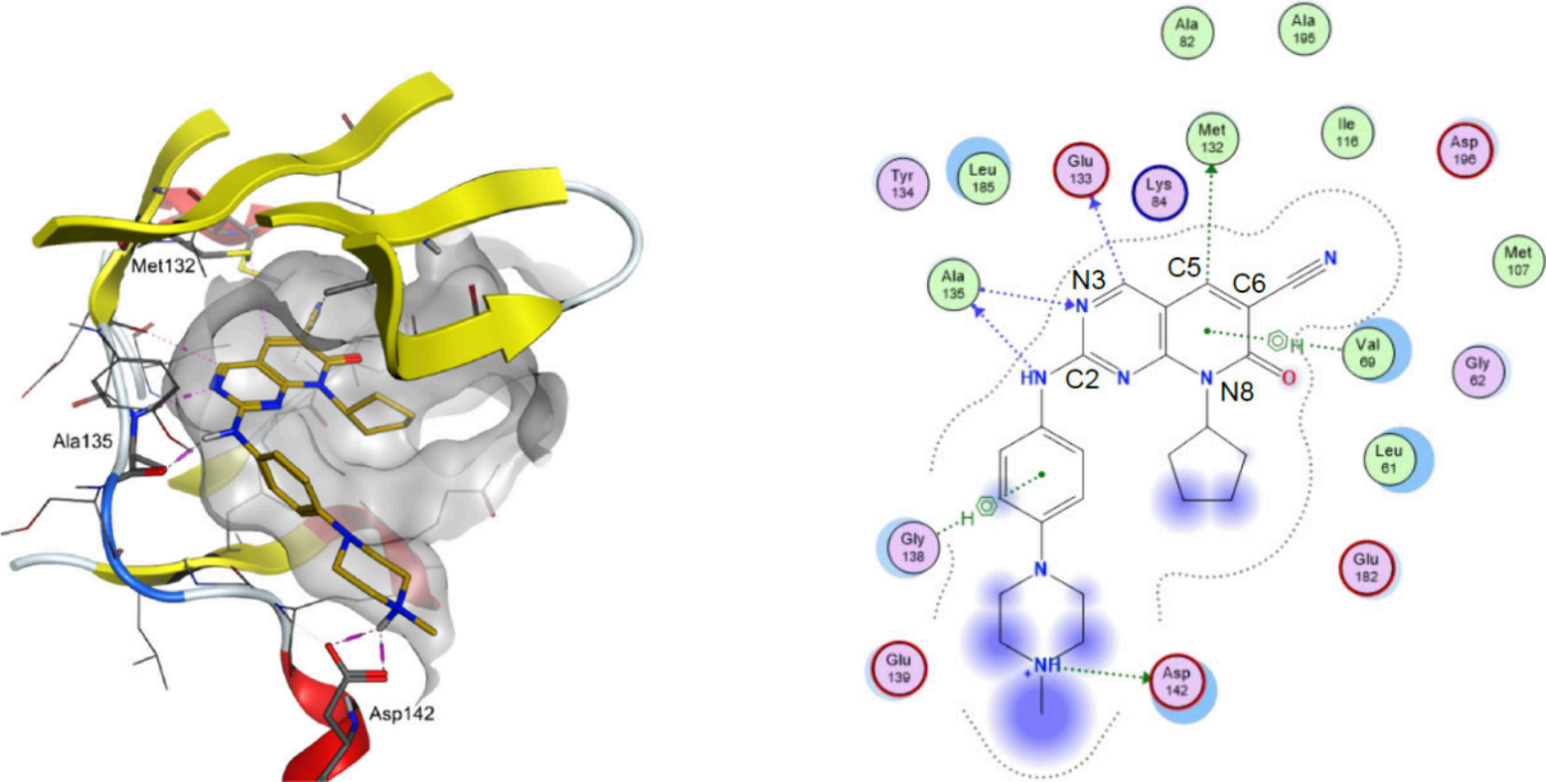
Best docking pose of **3** (gold) in a homology model of NUAK1 based on PDB structure 5EAK, with 2D interaction map.

In order improve the potency and selectivity of the series we initially investigated changes to the ring fused to the amino-pyrimidine core of **3** (Table 1). Substituents in the adjacent hydrophobic pocket are known to contribute to the selectivity of AMPK-related protein kinase inhibitors.^39,40^ Furthermore, alternatives to the nitrile group at C(6) were designed to lower TPSA and remove a possible metabolic hotspot at C(5).

**Table 1:**
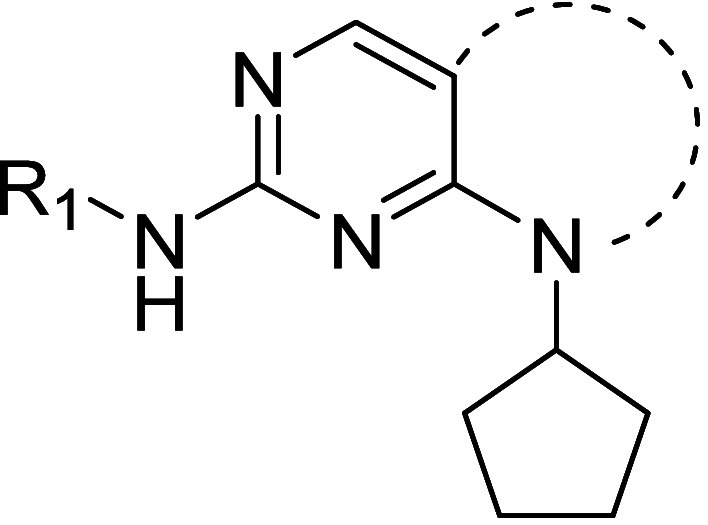

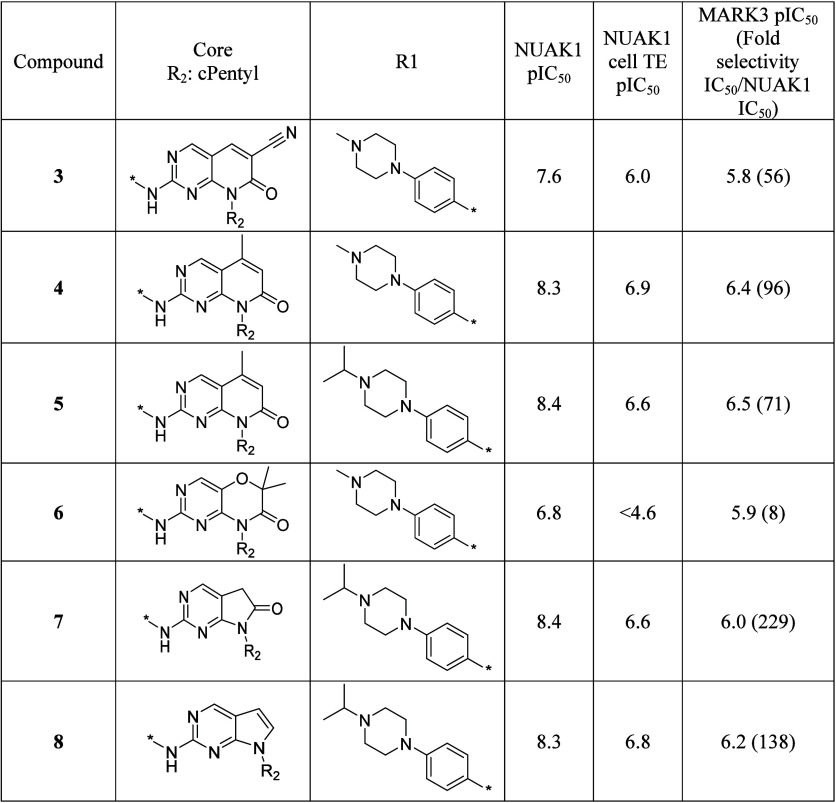
Effect of Core Changes on NUAK1 and MARK3 Potency^a^

a NUAK1: ADP-Glo assay; NUAK1 cell TE: cell-based NanoBRET target engagement assay.

Compound **4** incorporated the scaffold 5-methyl-7-oxo-7,8-dihydropyrido[2,3-*d*]pyrimidine previously used in CDK4 inhibitors.^39^**4** and its *N*-isopropyl analogue **5** proved to be significantly more potent than **3** and selectivity over MARK3 remained. A core change to give dimethylpyrimidooxazinone **6** was considered to reduce the planarity of the series, however this had a detrimental effect on potency and selectivity. The dihydropyrrolopyrimidone and pyrrolopyrimidine^41^ analogues (**7** and **8** respectively) were well tolerated and slightly improved the ligand efficiency of the scaffold (0.33 (**3**) vs 0.38 (**7**) and 0.39 (**8**)). As with **3**, a consistent observation across these series was the significant drop-off between the binding assay and cellular target engagement (TE) assay potencies (10–100x). Following this work, a similar approach was reported in the patent literature with additional core changes showing NUAK1 potency, including 6-methylpyrido[2,3-*d*]pyrimidin-7(8H)-one.^30^

We next explored the nature of the C(2) substituent, which was hypothesized to extend toward the mouth of the ATP pocket and Asp142 (Figure 2). Changing the piperazine from *N*-methyl to N-iso-propyl substituted or to 6-methyl-3,6-diazabicyclo[3.1.1]heptane did not improve potency (Table 2; compare **5** and **9** with **4**, and **10** with **3**), whereas moving the *N*-methylpiperazine from the para- to meta- position of the aniline ring did improve potency slightly (compare **11** with **3**). Ortho-methoxy phenyl substitution (compare **12** with **1**) or phenyl replacement with 2-pyridine (compare **13** with CDK4/6 inhibitor Palbociclib (**14**)^40^) reduced potency. The nonbasic compounds **15**, ON123790 (**16**) and **17** showed comparable potency to the *N*-methyl piperazine analogues, whereas the pyrazole analogue **18** did not. Interestingly, with a trimethoxyphenyl aniline, the dimethylpyrimidooxazinone analogue **19** was more potent than its piperazine analogue **6**, possibly indicating a change to the conformational preference of the ligand. While **16**, **17** and **19** were active in the biochemical assay, they appeared inactive in the cell assay, possibly due to poor solubility.

**Table 2:**
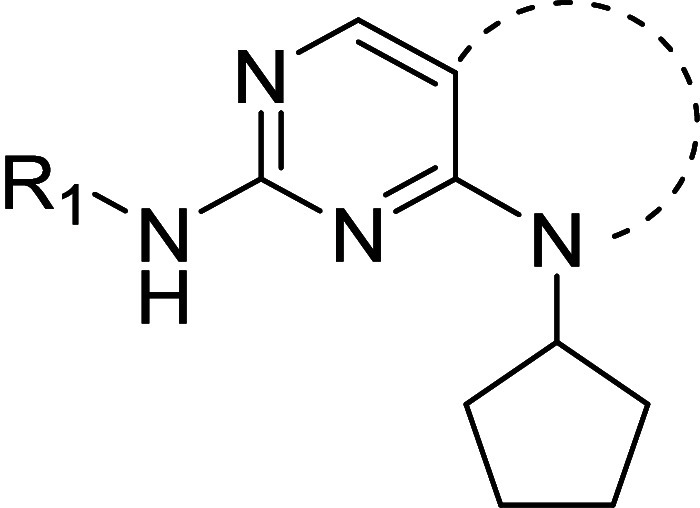

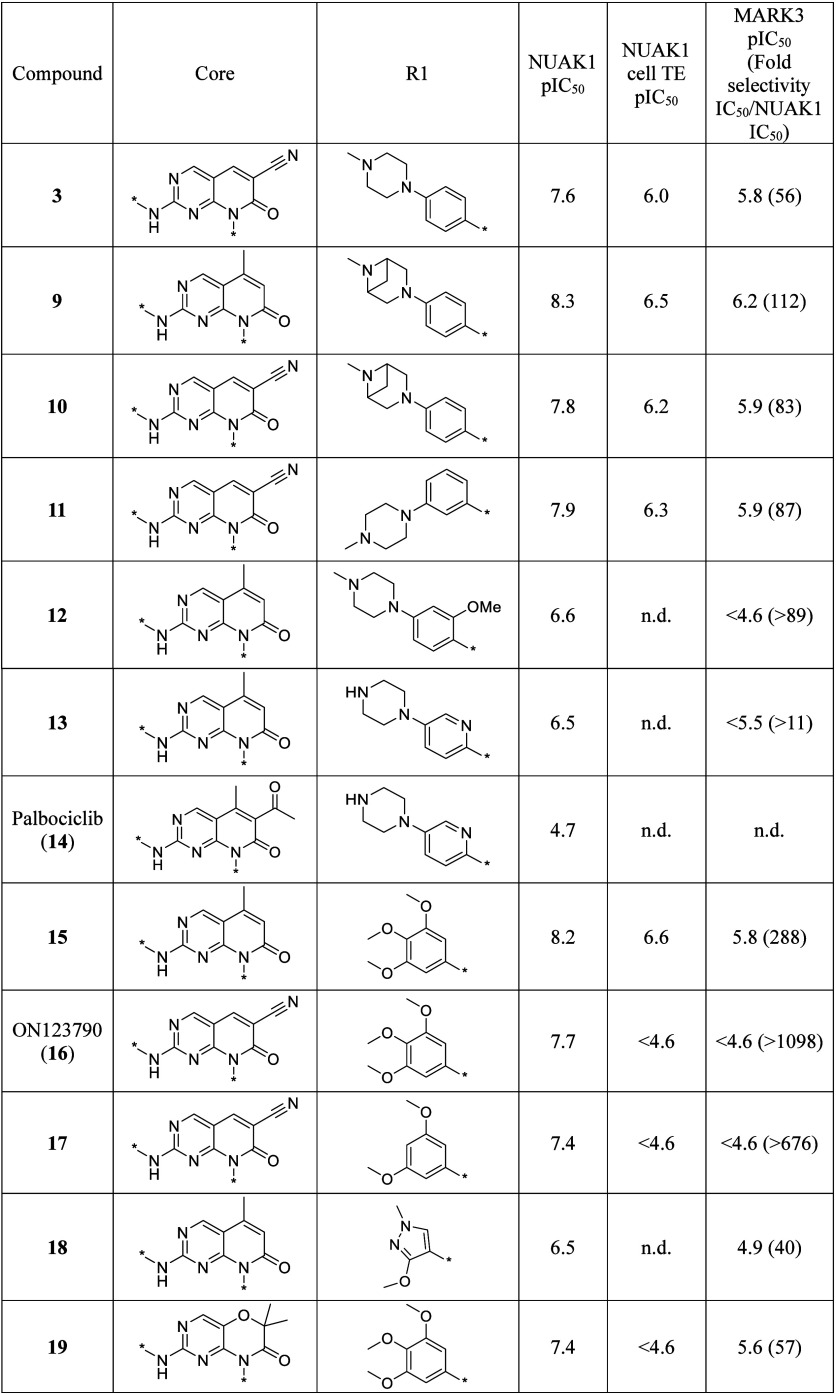
Effect of Aniline Changes on NUAK1 and MARK3 Potency^a^

a NUAK1: ADP-Glo assay; NUAK1 cell TE: cell-based NanoBRET target engagement assay.

The N(8) substituent was predicted to project into the ribose binding pocket of NUAK1 (Figure 2), hence more polar analogues were considered in an effort to improve affinity and metabolic stability. Changing the core N-substitution from cyclopentyl to acyclic groups reduced potency (see **20**-**23**, Table 3). In the pyrrolopyrimidine series, replacing the cyclopentyl group of **8** with methyl-tetrahydropyran derivatives (**24**-**26**) and methyl-tetrahydrofuran (**27**) maintained high NUAK1 potency, though showed structural similarity with inhibitors of ACK.^42^ A methylene spacer appeared to be beneficial for potency with direct linked heterocycles showing a slight reduction in potency (compare **28** and **29** with **30** and **31**). **24** and **25** were the most potent compounds in the cellular target engagement assay across all analogues of **3** tested (pIC_50_ both 7.0) with excellent selectivity over MARK3 (>1000x).

**Table 3:**
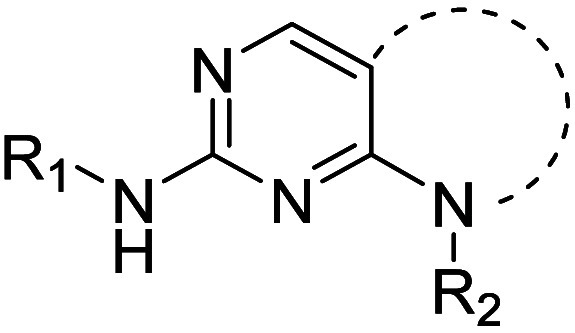

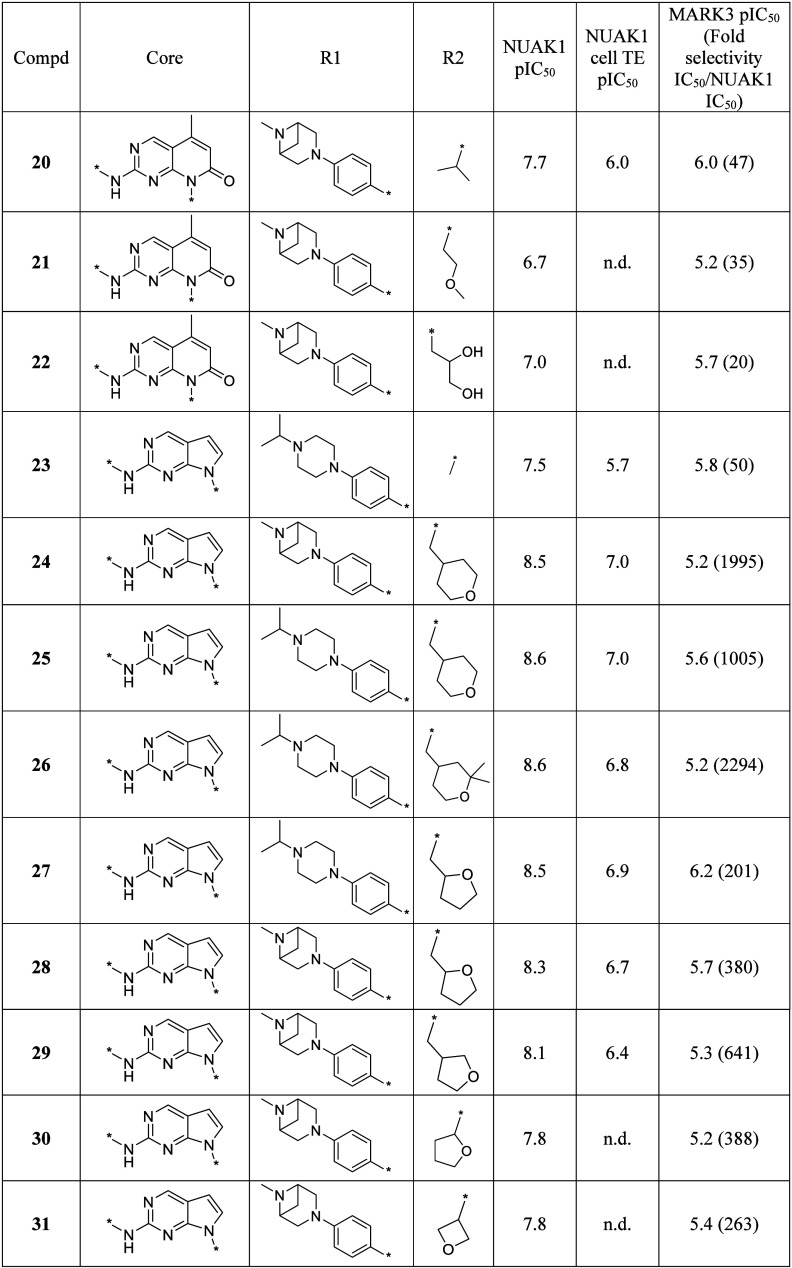
Effect of Core N-Substitution on NUAK1 and MARK3 Potency^a^

a NUAK1: ADP-Glo assay; NUAK1 cell TE: cell-based NanoBRET target engagement assay.

Improvement of rodent metabolic stability over **3** was a key aim for this series in order to deliver a chemical probe for use in mouse models, hence selected compounds were screened in a mouse liver microsomal clearance assay to compare in vitro half-lives (see Table 4 and Table S1). Replacement of the core did not have a significant effect (compare **4** and **7** with **3**), although pyrrolopyrimidine **8** was slightly more stable (MLM *t*_1/2_ 15.1 min). While the iso-propyl substituted piperazine **5** did not show improved stability over the metabolically labile methyl piperazine **4**, diazabicyclo[3.1.1]heptane analogues had extended half-lives (**9**: MLM *t*_1/2_ 20.9 min and **10**: MLM *t*_1/2_ 33.1 min) potentially due to lower lipophilicity.^43^ The nonbasic analogue of **3** (ON123790 (**16**)) had a shorter MLM *t*_1/2_ than the parent compound.

**Table 4:** Mouse Liver Microsome Half-Lives and Calculated Physicochemical Properties

Compound	MLM *t*_1/2_ (mins)	MW/cLogD_7.4_^a^/PSA
**3**	8.4	430/1.89/88
**4**	6.2	419/2.94/65
**5**	2.2	447/3.01/65
**7**	9.5	421/2.38/65
**8**	15.1	405/3.16/49
**9**	20.9	431/1.91/65
**10**	33.1	442/0.82/88
ON123790 (**16**)	1.9	421/2.10/110
**20**	79.9	405/1.48/65
**24**	21.4	419/1.78/59

a cLogD_7.4_ calculated using SimulationsPlus ADMET Predictor.

Changes to the cyclopentyl group generally gave an increase in metabolic stability, as exemplified by the isopropyl analogue **20** (MLM *t*_1/2_ 79.9 min), although these compounds were not as potent in the cell-based assay. The more potent cyclic ether-substituted pyrrolopyrimidines showed modest stability with tetrahydropyran analogue **24** having a MLM *t*_1/2_ of 21.4 min.

To investigate the effect of increasing mouse microsomal stability on in vivo half-lives, **9**, **10** and **24** were coadministered with **3** in a mouse PK cassette (5 mgkg^–1^, I.P.) (Table 5). **24** showed low overall exposure with the shortest half-life of the compounds tested, which may be due in part to the higher plasma free-fraction enabling faster rates of metabolic clearance. MDCK experiments also indicated a high Pgp-dependent efflux ratio for this compound. While **9** and **10** had higher plasma exposure than **3**, they showed the same plasma half-life, which was not predicted from the microsomal data and suggests potential secondary metabolism. At 2 h, **9** and **10** had similar brain exposure to **3** despite significantly different efflux ratios, but **3** had a higher total brain-to-plasma ratio. The partition coefficients of unbound compound (K_p,uu,b_ values) were marginally improved for **9**, **10** and **24** over **3**. While overall brain exposure has not been increased compared to **3**, an improvement in the potential for cellular target engagement of NUAK1 with these compounds has been achieved.

**Table 5:** ADME and Pharmacokinetic Data (mouse, 5 mg/kg, I.P.)

Parameter	Compound **3**	Compound **9**	Compound **10**	Compound **24**
MLM *t*_1/2_ (min)	8.4	20.9	33.1	21.4
Plasma *t*_1/2_ (h)	0.9	0.9	1.1	0.7
AUC_inf_ (hr·ng/mL)	897	1358	1131	365
[Brain] at 30 min (ng/mL)	1157	573	293	220
[Brain] at 2 h (ng/mL)	181	176	146	26.9
Brain/Plasma (30 min)	1.9	0.8	0.7	0.9
PPB F_u_	0.036^a^	0.011	0.019	0.152
BPB F_u_	0.006^a^	0.006	0.010	0.090
MDCK Papp cms^–1^ (ER)	1.98 (5.6)	1.01 (4.8)	1.24 (50)	1.05 (71)
K_p,uu,b_ (30 min)	0.29^a^	0.40	0.37	0.52

a Using data from Lv et al.^44^

In general, the compounds described showed good selectivity toward MARK3 (50- to 100-fold), with pyrrolopyrimidine analogues showing particularly high selectivity, especially with large core N-substituents (e.g., **26**, >1000-fold). All compounds tested against MARK1, MARK2 and MARK4 were less potent toward these isoforms than toward MARK3 (Table 6 and Table S2).

**Table 6:** Kinase Selectivity Data for Selected Compounds

Compound	NUAK1 pIC_50_	NUAK2 pIC_50_	AMPKA pIC_50_	MARK1 pIC_50_	MARK2 pIC_50_	MARK3 pIC_50_	MARK4 pIC_50_	CDK2^a^ % Inh.	CDK4^a^ % Inh.	CDK6^a^ % Inh.
**3**	7.6	8.3	5.9	5.2	4.8	5.8	5.0	n.d.	n.d.	n.d.
**4**	8.3	7.6	n.d.	5.5	5.5	6.4	5.4	57%	98%	87%
**7**	8.4	6.9	6.0	5.5	5.4	6.0	5.2	32%	75%	59%
**8**	8.3	7.4	5.5	5.8	5.7	6.2	5.6	84%	90%	79%
**9**	8.3	7.8	5.9	44%^b^	2%^b^	6.2	51%^b^	38%	91%	67%
**10**	7.8	7.0	6.3	n.d.	n.d.	5.9	n.d.	94%	98%	96%
**11**	7.9	n.d.	5.2	n.d.	n.d.	n.d.	n.d.	n.d.	n.d.	n.d.
**20**	7.7	7.2	n.d.	n.d.	n.d.	n.d.	n.d.	17%	46%	39%
**24**	8.5	7.6	5.1	5.1	4.8	5.2	4.9	29%	29%	11%

a CDK2/Cyclin A, CDK4/Cyclin D1 and CDK6/Cyclin D1 kinase activity data, % inhibition single point at 1 μM performed at Thermo Fisher Scientific, Z′-LYTE screening assay for CDK2 and Adapta screening assay for CDK4 and CDK6.

b MARK kinase activity data, single point % inhibition at 1 μM performed at the MRC PPU International Centre for Kinase Profiling, University of Dundee (radiometric filter binding assay). For data on additional compounds, see Supporting Information.

Selectivity toward NUAK2 was variable among the set of analogues tested (2.5- to 200-fold), with **3** as the only compound with a greater NUAK2 potency relative to NUAK1 (Table 6 and Table S3).

Selected compounds were tested against AMPKA (Table 6 and Table S4), with the majority showing >100- to 1000-fold selectivity toward NUAK1. **3** and **10** showed the least selectivity (47-fold and 35-fold respectively), though interestingly the meta-substituted piperazine analogue **11** was significantly more selective (500-fold).

With respect to CDK potency, selected compounds were tested at 1 μM to establish selectivity over CDK2, CDK4 and CDK6 (Table 6). **3** has been shown to be a potent inhibitor of all 3 isoforms,^28,45^ which was recapitulated in its diazabicyclo[3.1.1]heptane analogue **10**. Conversion to the 5-methyl-7-oxo-7,8-dihydropyrido[2,3-*d*]pyrimidine core (**4**) appeared to remove CDK2 activity but retained CDK4 and CDK6 (as previously reported^39^). The diazabicyclo[3.1.1]heptane analogue **9** reduced CDK6 and replacement of the cyclopentyl group with iPr **20** removed nearly all CDK potency (as previously reported^39^). The lactam analogue **7** was marginally more selective than other cores with only modest activity toward CDK4. The pyrrolopyrimidine core appeared to retain pan-CDK potency (see **8**) though again, replacement of the c-pentyl ring with methyl-tetrahydropyran (**24**) reduced activity toward all 3 isoforms, while retaining high NUAK1 potency (pIC_50_ 8.5).

Against a wider panel of 140 protein kinases, **9** and **24** showed good selectivity, with NUAK1 as the main target for both and at least 10-fold less active toward all other kinases tested in this screen, apart from ULK2 (**9** pIC_50_ 7.4) or JAK3 (**24** pIC_50_ 7.6) (Table S5). The only additional kinase that **24** showed significant activity toward was SIK2 (pIC_50_ 7.1). **9** showed weaker inhibition (pIC_50_ > 7) of MELK, MAP4K, YES1, NTRK1 and STK24, giving a different selectivity profile than that of **3**.^28^

Overall, we report new NUAK1 inhibitors based on **3** with improved selectivity vs NUAK2 and CDKs. Compared to **3**, selected examples show increased NUAK1 activity in a biochemical assay and a cellular measure of target engagement. Further, these compounds have retained brain penetration and plasma half-lives when measured alongside **3** in mice. We hope these tool molecules will be of value to the research community.

## Supporting Information Available

Additional assay data, biological assay procedures, in vivo experiment details, synthesis and characterization of all compounds (PDF)

## Notes

No unexpected safety issues were noted from this work. Novel compounds were assumed toxic as a precaution.

## Abbreviations

ADME: Absorption, distribution, metabolism and excretion
ADP: Adenosine diphosphate
AMP: Adenosine monophosphate
ATP: Adenosine triphosphate
AUC: Area under the curve
BPB: Brain protein binding
CNS: Central nervous system
ER: Efflux ratio
F_u_: Fraction unbound
HLM: Human liver microsomes
K_p,uu,b_: Unbound partition coefficient (brain to plasma)
MDCK-MDR1: Madin-Darby canine kidney-multidrug resistance mutation 1
MLM: Mouse liver microsomes
MW: Molecular weight
ND: Not determined
P_app_: Apparent permeability coefficient
PDB: Protein database
Pgp: P-glycoprotein
PPB: Plasma protein binding
SAR: Structure–activity relationship
TE: Target engagement
