# Supplementary material for: Development of the Pyrido[2,3-d]pyrimidin-7(8H)-one Scaffold toward Potent and Selective NUAK1 Inhibitors
Source: ACS Med Chem Lett. 2025 Jan 25;16(2):327–35. doi: 10.1021/acsmedchemlett.4c00579 (PMC11831560; doi:10.1021/acsmedchemlett.4c00579)
Supplement: Supplementary file 1 — ml4c00579_si_001.pdf [file ml4c00579_si_001.pdf]

Development of the Pyrido[2,3-*d*]pyrimidin-7(8*H*)-one Scaffold towards Potent and Selective NUAK1 Inhibitors

Timothy P. C. Rooney, Gregory G. Aldred, David Winpenny, Helen Scott, Henriette M. G. Willems, Iryna Voytyuk, Jonathan H. Clarke, Helen K. Boffey*, Stephen P. Andrews, John Skidmore*

Corresponding authors

Helen Boffey; Email: hkb32@cam.ac.uk

John Skidmore; Email: js930@cam.ac.uk

Affiliations

The ALBORADA Drug Discovery Institute, University of Cambridge, Island Research Building, Cambridge Biomedical Campus, Hills Road, Cambridge, CB2 0AH, United Kingdom

Supplementary Information

Table of Contents

	Page
Abbreviations	S2
Supplementary Tables	S3
Table S1: ADME properties of NUAK1 inhibitors	S3
Table S2: MARK kinase activity data	S3
Table S3: NUAK2 kinase activity data	S4
Table S4: AMPKA kinase activity data	S5
Table S5: Kinase panel data	S5
Biological experimental procedures	S10
ADMET and PK experimental methods	S12
Homology modelling and docking	S12
Synthetic schemes	S13
Synthetic chemistry methods	S17
Table 1 compounds	S18
Table 2 compounds	S25
Table 3 compounds	S35
NMR spectra and LC-MS traces of compounds from Table 4	S49
References	S61

Abbreviations

ADMET: Absorption, distribution, metabolism, excretion and toxicity, aq: aqueous solution, BPB: Brain protein binding, DCE: 1,2-dichloroethane, DCM: dichloromethane, eq: equivalents, DMSO: dimethylsulfoxide, ER: Efflux ratio, ESI: electrospray ionisation, F_u: Fraction unbound, HLM: Human liver microsomes, HPLC: high-performance liquid chromatography, HRMS: High resolution mass spectrometry, hrs: hours, IPA: iso-propanol, LiHMDS: Lithium bis(trimethylsilyl)amide, *m*CPBA: 3-chlorobenzene-1-carboperoxoic acid, MDCK-MDR1: Madin-Darby canine kidney-multidrug resistance mutation 1, min: minutes, MS: mass spectrometry, MLM: Mouse liver microsomes, NMR: nuclear magnetic resonance, P_{app}: Apparent permeability coefficient, PPB: Plasma protein binding, RMSD: root mean square deviation, rt: room temperature, SCX: strong cation exchange, UPLC: ultra high-performance liquid chromatography. XantPhos: (9,9-dimethyl-9*H*-xanthene-4,5-diyl)bis(diphenylphosphane).

Supplementary tables

Table S1 ADME properties of NUAK1 inhibitors performed at [a]Cyprotex Discovery and [b]ChemPartner Co. Ltd.

Compound	MLM[a] $t_{1/2}$ (mins)	HLM[a] $t_{1/2}$ (mins)	MDCK[a] P_{app} (cms^{-1}) (ER)	PPB[b] F_u	BPB[b] F_u
**3**	8.4	6.9	1.98 (5.6)		0.012
**4**	6.2				
**5**	2.2				
**7**	9.5				
**8**	15.1				
**9**	20.9		1.01 (4.8)	0.011	0.006
**10**	33.1		1.24 (49.5)	0.019	0.01
**11**	2.5	9.1			
**16**	1.9	13.6			
**19**	1.4				
**20**	79.9				
**23**	2.9	125			0.066
**24**	21.4		1.05 (70.8)	0.152	0.09
**25**	13.9				
**26**	14.8				
**27**	8.4				0.039
**28**	17.0				
**29**	12.8				

Table S2: MARK kinase activity data (ADP-Glo assay)

Compound	NUAK1 pIC_{50}	MARK1 pIC_{50} (Fold selectivity IC_{50}/NUAK1 IC_{50})	MARK2 pIC_{50} (Fold selectivity IC_{50}/NUAK1 IC_{50})	MARK3 pIC_{50} (Fold selectivity IC_{50}/NUAK1 IC_{50})	MARK4 pIC_{50} (Fold selectivity IC_{50}/NUAK1 IC_{50})
**3**	7.58	5.23 (224)	4.8 (603)	5.83 (56)	5.02 (363)
**4**	8.34	5.53 (646)	5.47 (741)	6.36 (95)	5.4 (871)

**5**	8.35	5.42 (851)	5.25 (1259)	6.5 (71)	5.04 (2042)
**7**	8.38	5.53 (708)	5.35 (1072)	6.02 (229)	5.19 (1549)
**8**	8.34	5.84 (316)	5.72 (417)	6.2 (138)	5.63 (513)
**24**	8.5	5.05 (2818)	4.81 (4898)	5.22 (1905)	4.9 (3981)
**25**	8.59	5.45 (1380)	5.15 (2754)	5.59 (1000)	4.94 (4467)
**26**	8.56	4.97 (3890)	4.95 (4074)	5.2 (2291)	<4.6 (>4700)
**27**	8.47	5.91 (363)	5.69 (603)	6.17 (200)	5.58 (776)
**28**	8.26	5.37 (776)	5.24 (1047)	5.68 (380)	5.19 (1175)

Table S3: NUAK2 kinase activity data (ADP-Glo assay)

**Compound**	**NUAK1 pIC_{50}**	**NUAK2 pIC_{50} (Fold selectivity IC_{50}/NUAK1 IC_{50})**
**3**	7.58	8.25 (0)
**4**	8.34	7.55 (6)
**7**	8.38	6.85 (34)
**8**	8.34	7.36 (10)
**9**	8.26	7.83 (3)
**10**	7.83	7.01 (7)
**20**	7.68	7.16 (3)
**24**	8.5	7.57 (9)
**26**	8.56	7.41 (14)
**27**	8.47	7.91 (4)
**28**	8.26	7.27 (10)

Table S4: AMPKA kinase activity data (ADP-Glo assay)

**Compound**	**NUAK1 pIC50**	**AMPKA pIC50 (Fold selectivity IC_{50}/NUAK1 IC_{50})**
**3**	7.58	5.91 (47)
**5**	8.35	5.97 (240)
**7**	8.38	6.04 (219)
**8**	8.34	5.46 (759)
**9**	8.26	5.91 (224)
**10**	7.83	6.28 (35)
**11**	7.86	5.15 (513)
**15**	8.21	<4.12 (>12000)
**23**	7.47	5.24 (170)
**24**	8.5	5.05 (2818)
**25**	8.59	5.57 (1047)
**26**	8.56	5.13 (2692)
**27**	8.47	6.00 (295)
**28**	8.26	5.45 (656)
**29**	8.1	5.25 (708)

Table S5. Kinase selectivity screening for **9** and **24** at 1 μM against a general kinase panel of 140 targets in radiometric filter binding assay using 33P-γ–ATP at the MRC PPU International Centre for Kinase Profiling, University of Dundee. pIC_{50} values were determined for compounds with <20% activity remaining at 1 μM.

	**Compound 9**			**Compound 24**		
**Kinase**	**% activity remaining**	**s.d.**	**pIC_{50}**	**% activity remaining**	**s.d.**	**pIC_{50}**
ABL	**41**	5		**56**	5	
AMPK (hum)	**19**	2		**81**	3	

ASK1	**97**	1		**99**	9	
Aurora A	**52**	5		**54**	6	
Aurora B	**61**	13		**64**	12	
BRK	**67**	6		**58**	4	
BRSK1	**81**	2		**75**	4	
BRSK2	**90**	4		**75**	2	
BTK	**91**	11		**82**	7	
CAMK1	**102**	10		**129**	8	
CAMKKb	**86**	6		**43**	3	
CDK2-Cyclin A	**59**	4		**79**	3	
CDK9-Cyclin T1	**67**	3		**56**	13	
CHK1	**57**	9		**75**	0	
CHK2	**72**	11		**98**	7	
CK1γ2	**34**	4		**107**	11	
CK1δ	**66**	14		**93**	2	
CK2	**108**	6		**96**	3	
CLK2	**23**	6		**90**	11	
CSK	**104**	2		**97**	10	
DAPK1	**60**	2		**125**	11	
DDR2	**39**	1		**82**	9	
DYRK1A	**78**	3		**141**	3	
DYRK2	**103**	6		**103**	5	
DYRK3	**74**	14		**65**	8	
EF2K	**72**	10		**102**	0	
EIF2AK3	**41**	4		**86**	12	
EPH-A2	**77**	5		**76**	4	
EPH-A4	**90**	4		**91**	6	
EPH-B1	**51**	3		**87**	12	
EPH-B2	**60**	10		**82**	4	
EPH-B3	**95**	1		**77**	4	
EPH-B4	**61**	3		**86**	3	
ERK1	**97**	5		**98**	0	
ERK2	**93**	0		**114**	12	
ERK5	**80**	9		**102**	1	
ERK8	**47**	2		**76**	5	

FGF-R1	**56**	5		**81**	11	
GCK	**76**	4		**89**	13	
GSK3b	**101**	0		**69**	14	
HER4	**118**	11		**74**	14	
HIPK1	**88**	5		**82**	2	
HIPK2	**54**	2		**85**	6	
HIPK3	**112**	11		**73**	9	
IGF-1R	**18**	3	5.98	**104**	5	
IKKb	**93**	1		**103**	1	
IKKe	**84**	11		**93**	11	
IR	**14**	1	6.58	**87**	5	
IRAK1	**75**	10		**71**	6	
IRAK4	**100**	10		**71**	6	
IRR	**70**	8		**94**	2	
JAK3	**22**	8		**7**	1	7.63
JNK1	**47**	3		**97**	6	
JNK2	**35**	3		**88**	1	
JNK3	**30**	2		**107**	13	
Lck	**64**	1		**67**	4	
LKB1	**104**	8		**100**	9	
MAP4K3	**29**	3		**62**	4	
MAP4K5	**14**	2	6.96	**78**	3	
MAPKAP-K2	**110**	2		**95**	15	
MAPKAP-K3	**84**	6		**109**	3	
MARK1	**56**	10		**94**	3	
MARK2	**98**	8		**101**	11	
MARK3	**35**	2		**104**	18	
MARK4	**49**	7		**95**	2	
MEKK1	**85**	12		**63**	4	
MELK	**6**	0	7.27	**95**	12	
MINK1	**30**	1		**79**	6	
MKK1	**108**	7		**71**	5	
MKK2	**94**	3		**91**	3	
MKK6	**84**	6		**88**	9	
MLK1	**21**	4		**33**	1	
MLK3	**43**	7		**91**	4	

MNK1	**96**	0		**72**	8	
MNK2	**56**	8		**89**	9	
MPSK1	**114**	14		**76**	15	
MSK1	**61**	0		**84**	8	
MST2	**62**	9		**91**	6	
MST3	**17**	3	6.95	**84**	5	
MST4	**61**	1		**92**	0	
NEK2a	**36**	2		**77**	3	
NEK6	**105**	9		**98**	9	
**NUAK1**	**5**	1	8.26*	**14**	13	8.50*
OSR1	**67**	8		**70**	12	
p38a MAPK	**92**	3		**93**	3	
p38b MAPK	**91**	1		**108**	1	
p38d MAPK	**94**	6		**110**	6	
p38g MAPK	**97**	5		**92**	7	
PAK2	**97**	10		**107**	8	
PAK4	**50**	3		**89**	7	
PAK5	**64**	8		**99**	9	
PAK6	**78**	4		**118**	5	
PDGFRA	**57**	9		**90**	7	
PDK1	**90**	9		**95**	7	
PHK	**56**	1		**92**	1	
PIM1	**90**	1		**84**	5	
PIM2	**99**	2		**111**	25	
PIM3	**80**	11		**56**	2	
PINK	**96**	1		**99**	3	
PKA	**84**	4		**90**	6	
PKBa	**103**	10		**110**	12	
PKBb	**99**	3		**93**	12	
PKCa	**86**	9		**96**	2	
PKCz	**110**	3		**118**	3	
PKCγ	**72**	12		**81**	6	
PKD1	**20**	2		**54**	1	
PLK1	**32**	6		**64**	4	
PRAK	**99**	1		**83**	3	
PRK2	**75**	12		**71**	12	

RIPK2	**78**	1		**73**	11	
ROCK 2	**56**	8		**78**	3	
RSK1	**41**	7		**74**	11	
RSK2	**49**	4		**73**	14	
S6K1	**85**	11		**78**	12	
SGK1	**96**	6		**72**	15	
SIK2	**44**	4		**13**	0	7.09
SIK3	**85**	9		**74**	4	
SmMLCK	**61**	3		**102**	0	
Src	**53**	8		**84**	1	
SRPK1	**111**	12		**79**	14	
STK33	**101**	5		**93**	2	
SYK	**49**	9		**81**	4	
TAK1	**6**	1	6.68	**110**	3	
TAO1	**91**	3		**97**	4	
TBK1	**66**	9		**41**	14	
TESK1	**96**	1		**94**	3	
TGFBR1	**92**	4		**75**	12	
TIE2	**115**	7		**62**	12	
TLK1	**59**	7		**86**	1	
TrkA	**12**	0	7.01	**72**	6	
TSSK1	**56**	8		**77**	11	
TTBK1	**92**	8		**103**	7	
TTBK2	**97**	8		**96**	7	
TTK	**54**	9		**99**	2	
ULK1	**23**	2		**77**	3	
ULK2	**17**	5	7.39	**75**	7	
VEG-FR	**71**	9		**17**	3	6.53
WNK1	**103**	14		**84**	6	
YES1	**13**	3	7.12	**46**	10	
ZAP70	**94**	7		**95**	7	

* Data generated at ALBORADA Drug Discovery Institute using ADP-Glo assay

Biological experimental procedures

ADP-Glo biochemical assays

Protein kinase activity in the presence of inhibitor compounds was determined by ADP-Glo assay (Promega), as previously described.[1,2] Compounds were serialised using a Labcyte Echo 520 and transferred to 384 well plates (Greiner 784201) followed by the addition 5 µL of substrate containing peptide and ATP at K_m in buffer (see **Table S6**). 5 µL of purified protein was added and the plate was incubated for 60 minutes at room temperature (ng/well; see **Table S6**). 3 µL of assay mixture was transferred using an Integra Viaflo384 into a white low volume plate (Greiner 781904) prior to the addition of 3 µL of ADP-Glo Reagent™ for a further 40 minute incubation. After incubation with 6 µL of Kinase Detection Reagent for 30 minutes, plate luminescence was read (Pherastar FSX, BMG Labtech).

Buffer A

33 mM HEPES pH7.4, 0.1% CHAPS, 20 mM $MgCl_2$ and 16.7 µM EGTA

Buffer B

30 mM HEPES pH7.4, 3 mM $MgCl_2$, 3 mM $MnCl_2$, 1.2 mM DTT, 0.01% CHAPS

Table S6: Conditions for protein kinase ADP-Glo assays

Protein	ATP conc.	ATP Km	Peptide name	Peptide conc.	Peptide supplier	Enzyme amount /well	Enzyme supplier	Assay vol.	Buffer	Min. n[a]
NUAK1	25 µM	31 µM	Sakamototide	50 µM	Merck	6.7 ng	MRC PPU	10 µl	A	6
AMPK A α1β2γ1	10 µM	9.3 µM	Sakamototide	100 µM	Merck	1 ng	MRC PPU	10 µl	B	2
MARK1	20 µM	ND	CHKTide	100 µM	MRC PPU	1.2 ng	MRC PPU	10 µl	A	2
MARK2	20 µM	ND	CHKTide	100 µM	MRC PPU	4 ng	MRC PPU	10 µl	A	2
MARK3	10 µM	ND	CHKTide	200 µM	MRC PPU	2.65 ng	MRC PPU	10 µl	A	6
MARK4	50 µM	ND	CHKTide	100 µM	MRC PPU	2.65 ng	MRC PPU	10 µl	A	2
NUAK2	20uM	21uM	Sakamototide	100 µM	Merck	1.15 ng	MRC PPU	3 µl	A	2

[a]Min. n: Minimum number of technical replicates per compound

NanoBRET® target engagement assay

The binding of compounds to NUAK1 in intact cells was assessed using an NanoBRET® TE Intracellular Kinase Assay (Promega N2501). HEK293 cells transiently transfected with NanoLuc®-NUAK1 Fusion Vector (Promega NV1831) were incubated with 10 nL K5 tracer (provided in the kit, Promega N2501) and 40 nL of the test compounds in 100% DMSO in a white low volume non-binding 384 assay plate (Grenier Bio One 784904) for 90 min at 37 °C. After incubation, followed by cooling for 15 min at room temperature, 4 µL of the Intracellular TE Nano-Glo® Substrate/Inhibitor (Promega N2160) was added to each well. The plate was read using a Pherastar FSX plate reader (BMG Labtech). Minimum number of 2 technical replicates per compound.

Data Analysis:

Activity pIC_{50} values and standard error of the mean (SEM) were estimated using a 4-parameter fit (Dotmatics). The SEM for all reported data was 0.2 or lower. See Experimental Procedures for minimum number of replicates per compound. Each experiment included a Standard compound whose pIC_{50} value was compared to a quality control chart. If the Standard pIC_{50} value fell outside the range of pIC_{50} +/-3 standard deviation, then the experiment was invalidated.

CDK2/cyclin A: Z′-LYTE™ screening assay performed by Thermo Fisher Scientific: The 2X CDK2/cyclin A / Ser/Thr 12 mixture is prepared in 50 mM HEPES pH 7.5, 0.01% BRIJ-35, 10 mM $MgCl_2$, 1 mM EGTA. The final 10 µL Kinase Reaction consists of 1.22 - 10.3 ng CDK2/cyclin A and 2 µM Ser/Thr 12 in 50 mM HEPES pH 7.5, 0.01% BRIJ-35, 10 mM $MgCl_2$, 1 mM EGTA. After the 1 hour Kinase Reaction incubation, 5 µL of a 1:4096 dilution of Development Reagent A is added.

CDK4/cyclin D1: Adapta™ screening assay was performed by Thermo Fisher Scientific. The 2X CDK4/cyclin D1 / Rb Substrate mixture is prepared in 50 mM HEPES pH 7.5, 0.01% BRIJ-35, 10 mM $MnCl_2$, 1 mM EGTA, 2 mM DTT, 0.02% NaN_3. The final 10 µL Kinase Reaction consists of 7.5 - 30 ng CDK4/cyclin D1 and 1 µM Rb Substrate in 32.5 mM HEPES pH 7.5, 0.005% BRIJ-35, 5 mM $MnCl_2$, 0.5 mM EGTA, 1 mM DTT, 0.01% NaN3. After the 1 hour Kinase Reaction incubation, 5 µL of Detection Mix is added.

CDK6/cyclin D1: Adapta™ screening assay was performed by Thermo Fisher Scientific. The 2X CDK6/cyclin D1 / Rb Substrate mixture is prepared in 50 mM HEPES pH 7.5, 0.01% BRIJ-35, 10 mM $MnCl_2$, 1 mM EGTA, 2 mM DTT, 0.02% NaN3. The final 10 µL Kinase Reaction consists of 1.75 - 7 ng CDK6/cyclin D1 and 1 µM Rb Substrate in 32.5 mM HEPES pH 7.5, 0.005% BRIJ-35, 5 mM $MnCl_2$, 0.5 mM EGTA, 1 mM DTT, 0.01% NaN_3. After the 1 hour Kinase Reaction incubation, 5 µL of

Detection Mix is added.

All single point assay determinations carried out in duplicate.

ADMET and PK experimental methods

Microsomal stability: analysis was performed by Cyprotex Discovery. Briefly, test compounds in DMSO were incubated at a concentration of 1 µM (0.25% DMSO final) with mouse or human hepatic microsomes (0.5 mg protein/mL) in the presence of NADPH (1 mM) at 37 °C. Aliquots were taken at time intervals (0, 5, 15, 30 and 45 min) and stopped by transferring into acetonitrile, then analysed using generic LC-MS/MS conditions for compound remaining, allowing the determination of the half-life for the compound.

MDR1-MDCK Permeability (bi-directional): was performed by Cyprotex Discovery. Briefly, compounds were administered at 10 µM (1% DMSO final) to the apical or basolateral side of a confluent monolayer of MDR1- MDCK cells, then incubated at 37 °C for 60 minutes before appearance on the opposite side of the monolayer was determined LC-MS/MS. The efflux ratio (ER) is calculated from the ratio of B-A to A-B permeabilities.

Plasma protein binding: was performed by ChemPartner Co. Ltd. Briefly, compounds in DMSO (1 µM, 0.2% DMSO final) were added to mouse plasma or brain tissue and dialysed against buffer for 5 hrs at 37 °C. The compound concentration in each of the plasma and buffer compartments was determined by mass spectrometry and used to calculate the percentage compound bound.

Pharmacokinetic analysis: was performed by ChemPartner Co. Ltd. Male CD-1 mice (N=3 per timepoint) were intraperitoneally dosed at 5 mg/kg, as a cassette of five compounds, formulated in 10% DMSO, 10% Solutol HS 15, 80% (50 mM citrate buffer pH3.0) at 0.5 mg/mL. At the designated time points a blood sample was collected from the facial vein into K2EDTA tubes, and plasma separated by centrifugation. Mice were euthanised by CO_2 inhalation and brains were dissected and homogenised. Compound levels were quantified by LC-MS/MS and PK parameters estimated by a non-compartmental model using WinNonlin 8.2.

Homology modelling and docking

A BLAST search with the kinase domain sequence of human NUAK1 (residues 55-306) identified MARK2 as a protein with a homologous kinase domain for which a crystal structure was available. MOE (www.ccg.com) was used to align the NUAK1 kinase domain to human MARK2 structure pdb:5EAK. 25 homology models were built, based on this alignment, sampling 3 side chain conformations and including the 5EAK ligand (which has a reported affinity of 4.2 nM for NUAK1).

The final model was minimized to an RMSD gradient of 0.5. ON123300 was docked flexibly to this homology model with Glide SP (www.schrodinger.com). The 5EAK ligand was used to define the binding site. Post-docking minimization was carried out on 25 solutions, and strain corrections were applied. The top scoring pose was selected. This was further minimized in a flexible binding site in MOE (with QuickPrep, AMBER:EHT forcefield, binding site not tethered, atoms further than 8Å from binding site fixed), resulting in the pose shown in Figure **2** of the main text.

Synthetic schemes

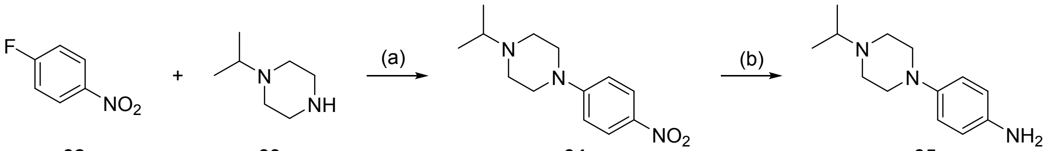

32 **33** **34** **35**

Scheme 1: *Reagents and conditions*: (a) Na_2CO_3, 1,4-dioxane, 80 °C, 16 hrs. (b) H_2, Pd/C, EtOH, rt, 16 hrs.

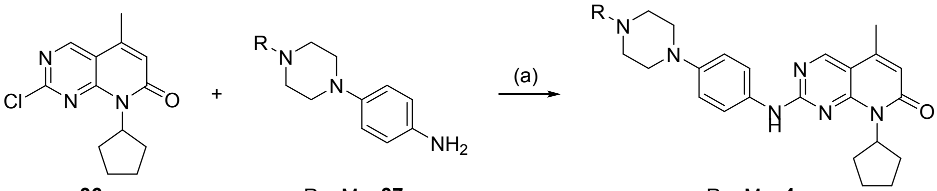

36

R = Me: **37**
R = iPr: **35**

R = Me: **4**
R = ^{i}Pr: **5**

Scheme 2: *Reagents and conditions*: (a) TFA, 2-BuOH, 120 °C, 3 hrs. OR $Pd_2(dba)_3{\cdot}CHCl_3$, XantPhos, K_3PO_4, 60 °C, 64 hrs.

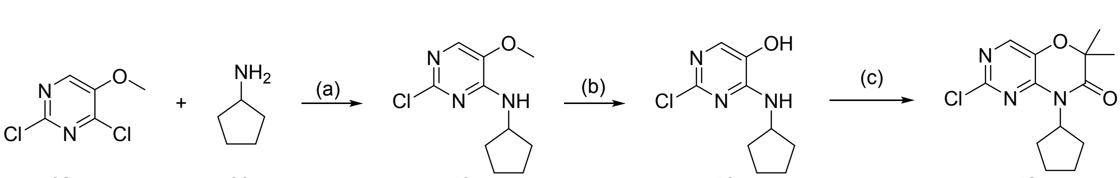

38 **39** **40** **41** **42**

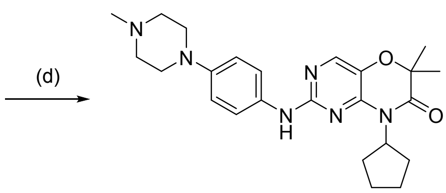

6

Scheme 3: *Reagents and conditions*: (a) Et_3N, THF, rt, 18 hrs. (b) BBr_3, CH_2Cl_2, rt, 27 hrs. (c) methyl 2-bromo-2-methylpropanoate, K_2CO_3, MeCN, 80 °C, 16 hrs. (d) **37**, TFA, 2-BuOH, 120 °C, 3 hrs.

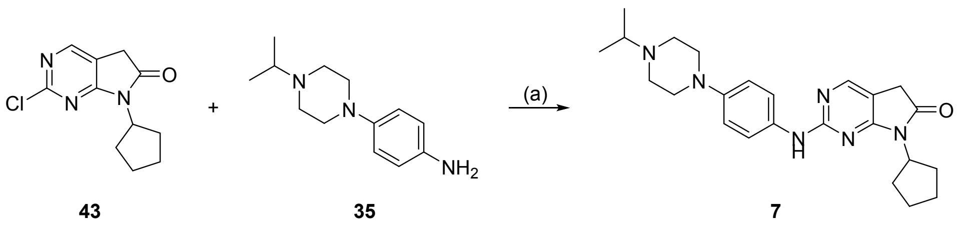

Scheme 4: *Reagents and conditions*: (a) TsOH, 2-BuOH, 125 °C, 1 hr.

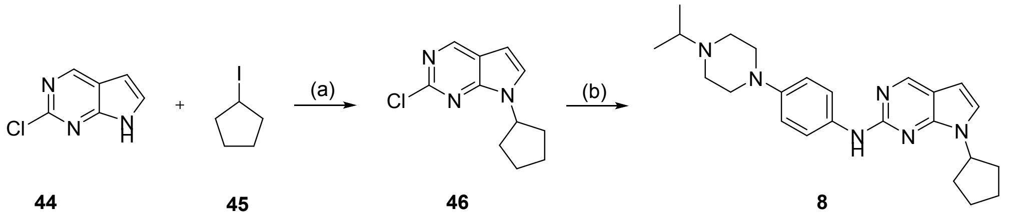

Scheme 5: *Reagents and conditions*: (a) NaH, DMF, rt, 18 hrs. (b) **35**, $Pd_2(dba)_3 \cdot CHCl_3$, Xantphos, Cs_2CO_3, 1,4-dioxane, 105 °C, 2 hrs.

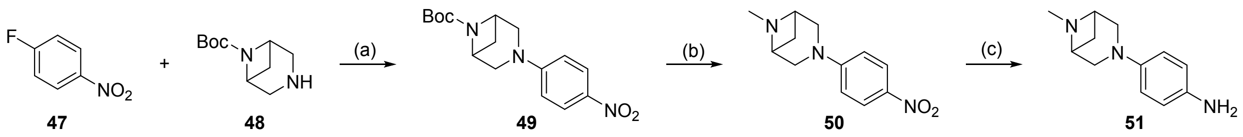

Scheme 6: *Reagents and conditions*: (a) Na_2CO_3, 1-4,dioxane, 90 °C, 18 hrs. (b) i) TFA, CH_2Cl_2, rt, 16 hrs. ii) HCHO, $NaBH(OAc)_3$, AcOH, DCE, rt, 16 hrs. (c) H_2, Pd/C, EtOH, rt, 16 hrs.

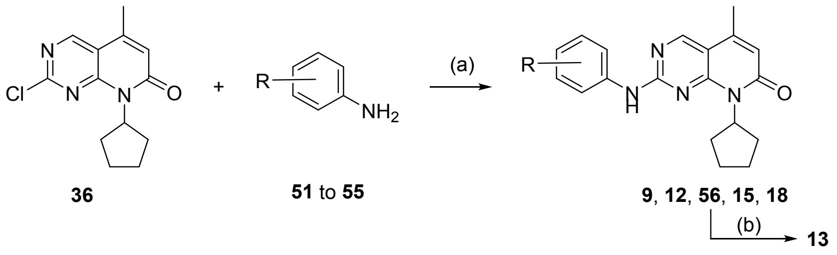

Scheme 7: *Reagents and conditions*: (a) TFA, 2-BuOH, 120 °C, 3 hrs; OR $Pd_2(dba)_3 \cdot CHCl_3$, XantPhos, K_3PO_4, 60 °C, 64 hrs. (b) TFA, CH_2Cl_2, rt 4 hrs.

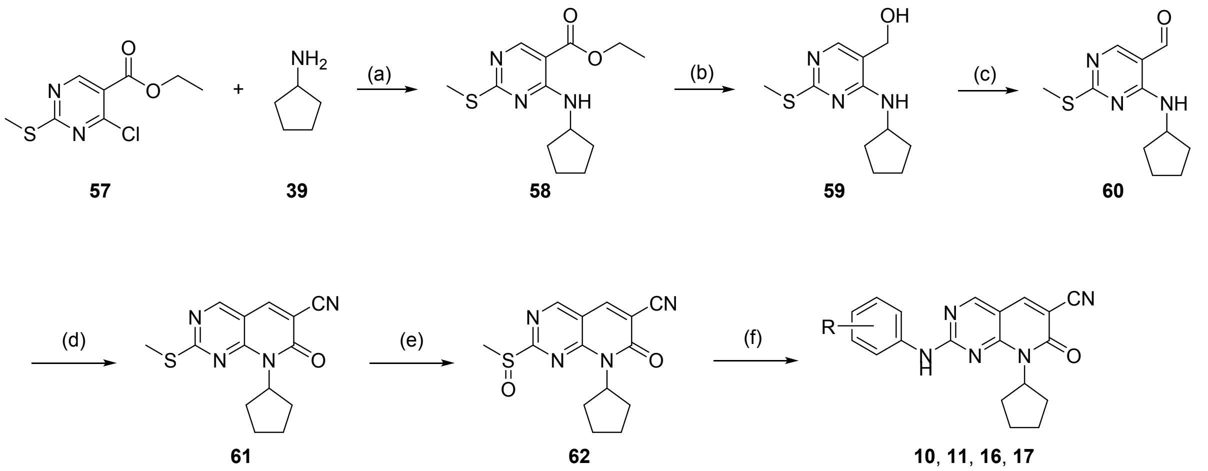

Scheme 8: *Reagents and conditions*: (a) Et_3N, THF, 25 °C, 18 hrs. (b) $LiAlH_4$, THF, 0 °C to rt, 24 hrs. (c) MnO_2, $CHCl_3$, rt, 17 hrs. (d) 2-cyanoacetic acid, AcOH, $BnNH_2$, 120 °C, 18 hrs. (e) *m*CPBA, CH_2Cl_2, rt, 2 hrs. (f) Aniline, toluene, 100 °C, 15 hrs; OR TsOH, 2-BuOH, 120 °C, 3 hrs.

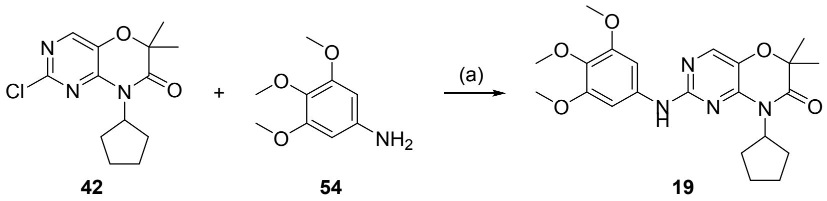

Scheme 9: *Reagents and conditions*: (a) TFA, 1-BuOH, 120 °C, 3 hrs.

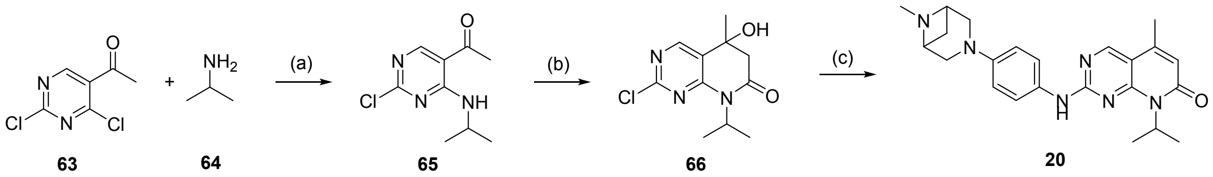

Scheme 10: *Reagents and conditions*: (a) $NaHCO_3$, THF, rt, 2 hrs. (b) EtOAc, LiHMDS, THF, −78 °C to rt, 2 hrs. (c) **51**, TsOH, 2-BuOH, 80 °C, 2 hrs.

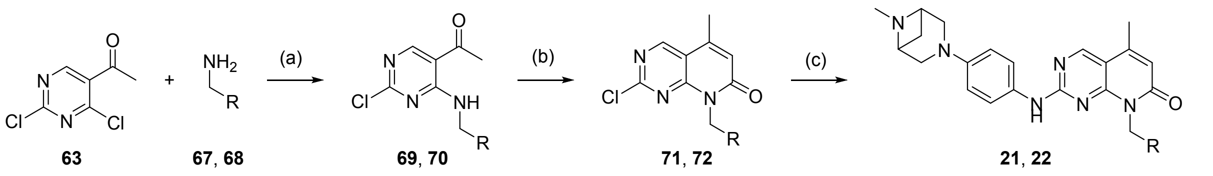

Scheme 11: *Reagents and conditions*: (a) $NaHCO_3$, THF, rt, 2 hrs. (b) EtOAc, LiHMDS, THF, −78 °C to rt, 2 hrs. (c) **51**, TsOH, 2-BuOH, 80 °C, 2 hrs.

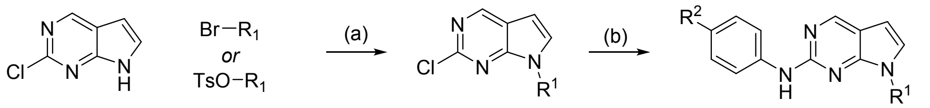

44 **73** to **78** **79** to **85** **23** to **31**

Scheme 12: *Reagents and conditions*: (a) NaH, DMF, rt 1 hr. (b) Requisite-aniline, $Pd_2(dba)_3{\cdot}CHCl_3$, Xantphos, K_3PO_4, 1,4-dioxane, 120 °C, 72 hrs.

Synthetic Chemistry Methods

All screening compounds are >95% pure by HPLC analysis.

Reagents and solvents were of commercially available reagent grade quality and used without further purification. Compound **3** was purchased from MedChemExpress (HY-12344) or Stratech (B6192-APE), compound **1** was purchased from Tocris (5622), compounds **2** and **14** were purchased from Generon (B1374 and A8316 respectively). Reactions requiring anhydrous conditions were carried out in oven dried glassware under an atmosphere of N_2. Reactions were monitored by thin-layer chromatography (TLC) on silica gel 60 F_{254} aluminium or glass supported sheets, or by liquid chromatography-mass spectrometry (LC-MS). Flash column chromatography was carried out on a Biotage Isolera One system using normal phase (SiO_2) or reverse phase (C18) cartridges. Compounds were loaded in solution or adsorbed onto Celite® 545 or ISOLUTE® HM-N and eluted using a linear gradient of the specified solvents. Purification by C18 reverse phase HPLC was carried using an Agilent 1260 Infinity machine and a Waters XBridge BEH C18 OBD column (130 Å, 5 µm, 30 mm × 100 mm) with a linear gradient of H_2O (with 0.1% NH_3) and MeCN (with 0.1% NH_3). LCMS analysis was performed on a Waters Aquity HClass UPLC system with a Aquity QDa for mass detection. High-resolution mass spectra (HRMS) were measured on a Waters Vion IMS QTof spectrometer. NMR spectra were recorded on a Bruker Avance III (^1H = 300 MHz, ^{19}F = 282 MHz) or a Bruker Avance III with Dual $^{13}C/^1H$ Cryoprobe (^{13}C = 126 MHz) spectrometer using the requisite solvent as a reference for internal deuterium lock. The chemical shift data for each signal are given as δ chemical shift (multiplicity, *J* values in Hz, integration) in units of parts per million (ppm) relative to tetramethylsilane (TMS) where δH (TMS) = 0.00 ppm. The multiplicity of each signal is indicated by: s (singlet), d (doublet), t (triplet), q (quartet), quint (quintet), sex (sextet), or m (multiplet). Signals from exchangeable protons are not always detected. UPLC analysis of final compounds was performed on a Waters Aquity HClass UPLC system and is reported as method name, retention time, UV % purity. The method parameters are as follows:

Method	Column	Additive	Flow rate	Gradient (time, %MeCN in H_2O)
A	BEH C18 (130 Å, 1.7 µm, 2.1 mm × 50 mm)	1 mM NH_3	0.6 mL/min	0 min, 5%; 0.8 min, 5%; 3.3 min, 95%; 4.3 min, 95%; 4.5 min, 5%; 5.5 min, 5%.
B	HSS C18 (100 Å, 1.8 µm, 2.1 mm × 50 mm)	0.1% HCO_2H	0.6 mL/min	0 min, 5%; 0.8 min, 5%; 3.3 min, 95%; 4.3 min, 95%; 4.5 min, 5%; 5.5 min, 5%.
C	BEH C18 (130 Å, 1.7 µm, 2.1 mm × 50 mm)	1 mM NH_3	0.6 mL/min	0 min, 5%; 0.8 min, 5%; 8.3 min, 95%; 9.3 min, 95%; 9.5 min, 5%; 10.5 min, 5%.
D	HSS C18 (100 Å, 1.8 µm, 2.1 mm × 50 mm)	0.1% HCO_2H	0.6 mL/min	0 min, 5%; 0.8 min, 5%; 8.3 min, 95%; 9.3 min, 95%; 9.5 min, 5%; 10.5 min, 5%.

General procedure A:

To a solution of aryl-chloride (1.0 eq.) and the requisite aniline (1.3 eq.) in 2-BuOH (0.1 M) was added trifluoroacetic acid (1.1 eq.) and the reaction mixture was heated at 120 °C for 3 hrs. Upon completion the reaction was cooled to rt, diluted with EtOAc, washed with sat. aq. $NaHCO_3$ and brine, then dried (hydrophobic frit) and concentrated in vacuo. Purification was performed via the specified method.

General procedure B:

A solution of aryl-methylsulfoxide (1.0 eq.) and the requisite aniline (1.2 eq.) in toluene (1.0 M) was heated at 100 °C for 16 hrs. Upon completion the reaction was cooled to rt, diluted with E, washed with sat. aq. $NaHCO_3$ and brine, then dried (hydrophobic frit) and concentrated in vacuo. Purification was performed via the specified method.

General procedure C:

To a solution of aryl-chloride (1.0 eq.) and the requisite aniline (1.2 eq.) in 2-BuOH (1.5 mL) was added *p*-toluenesulfonic acid monohydrate (2.0 eq.) and the reaction mixture was heated at 80 °C under MW irradiation for 2 hrs. After this time the reaction mixture was loaded onto an SCX-II column, washed with DCM and MeOH, then eluted with 0.5 M NH_3 in MeOH and concentrated in vacuo. Purification was performed *via* the specified method.

General procedure D:

A suspension of aryl-chloride (1.0 eq), the requisite aniline (1.5 eq), tris(dibenzylideneacetone)dipalladium (0) chloroform adduct (5 mol%), tripotassium phosphate (2.0 eq) and 4,5-bis(diphenylphosphino)-9,9-dimethylxanthene (10 mol%) in 1,4-dioxane (0.1 M) was degassed with N_2 for 10 mins before the reaction mixture was heated at 80 °C for 16 hrs. After this time another aliquot of catalyst was added and the reaction further heated at 120 °C for 72 hrs. The reaction was stopped, and the mixture loaded onto an SCX-II cartridge, washed with DCM and MeOH, then eluted with 0.5 M NH_3 in MeOH and concentrated in vacuo. The crude material taken up in DMSO before purification *via* prep HPLC with basic eluent (40-80% MeCN in H_2O) to yield desired product.

<u>Table 1 Compounds</u>

1-Isopropyl-4-(4-nitrophenyl)piperazine (34)

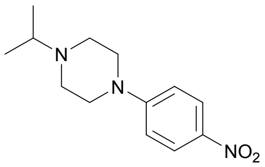

1-Fluoro-4-nitrobenzene (**32**) (250 mg, 1.77 mmol) was dissolved in 1,4-dioxane (10 mL) and (1-iso-propyl)piperazine (**33**) (0.260 mL, 1.77 mmol) was added followed by sodium carbonate (380 mg, 3.54 mmol). The reaction mixture was heated at 80 °C for 16 hrs. After this time a further 0.2 eq of (1-iso-propyl)piperazine (0.052 mL, 0.354 mmol) and sodium carbonate (0.076 g, 0.71 mmol) along with DMF (5 mL) and reaction further heated at 90 °C for 4 hrs. EtOAc added and the organic layer washed with sat. aq. $NaHCO_3$ and separated. The aqueous layer was extracted with EtOAc (×2) and the combined organic fractions were washed with brine and dried ($NaSO_4$) and solvent removed in vacuo. Crude material was purified via silica gel chromatography (0 to 5% MeOH (0.1% Et_3N) in DCM) to yield 1-isopropyl-4-(4-nitrophenyl)piperazine (**34**) (310 mg, 1.24 mmol, 70%) as a yellow solid. MS (ESI+) *m/z* calcd for $C_{13}H_{20}N_3O_2^+$ $[M + H]^+$ 250.3, found 250.3. UPLC analysis (method A), 2.74 min, >98% purity. ^1H NMR (300 MHz, $CDCl_3$) δ 8.18 – 8.06 (m, 2H), 6.97 – 6.75 (m, 2H), 3.48 – 3.38 (m, 4H), 2.81-2.72 (m, 1H), 2.69 – 2.57 (m, 4H), 1.08 (d, J = 8.4 Hz, 6H).

4-(4-Isopropylpiperazin-1-yl)aniline (35)

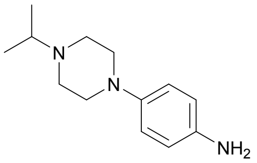

1-Isopropyl-4-(4-nitrophenyl)piperazine (**34**) (300 mg, 1.20 mmol) was taken up in ethanol (10 mL) and the vessel was evacuated and refilled with N_2 (×3). 10% Pd/C (50.0 mg) was added and evacuated once more. Hydrogen gas was introduced via balloon and vessel evacuated and refilled with H_2 (×3) before stirring at rt for 16 hrs. After this time the suspension was filtered through celite and the solvent removed in vacuo to yield 4-(4-isopropylpiperazin-1-yl)aniline (**35**) (175 mg, 0.80 mmol, 66%) as a purple solid. MS (ESI+) *m/z* calcd for $C_{13}H_{22}N_3^+$ $[M + H]^+$ 220.3, found 220.3. UPLC analysis (method A), 2.26 min, >95% purity. ^1H NMR (300 MHz, $CDCl_3$) δ 7.05 – 6.76 (m, 2H), 6.73 – 6.57 (m, 2H), 3.44 (br s, 2H), 3.15 – 3.05 (m, 4H), 2.77 – 2.65 (m, 5H), 1.11 (d, J = 9.1 Hz, 6H).

Caution! *Hydrogen is classified as a GHS Flammable Gas, Category 1. Hydrogen and the hydrogen balloon were handled using standard procedures.*

8-Cyclopentyl-5-methyl-2-((4-(4-methylpiperazin-1-yl)phenyl)amino)pyrido[2,3-*d*]pyrimidin-7(8*H*)-one (4)

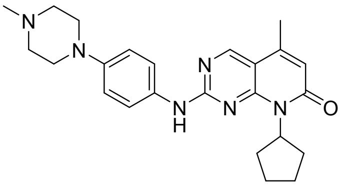

2-Chloro-8-cyclopentyl-5-methylpyrido[2,3-*d*]pyrimidin-7(8*H*)-one (**36**) (50.0 mg, 0.190 mmol) and 4-(4-methylpiperazino)aniline (**37**) (47.1 mg, 0.246 mmol) were reacted according to general procedure **A**. Purification via preparatory HPLC (0.1% NH_3 in MeCN/Water; 30-95%) yielded 8-cyclopentyl-5-methyl-2-((4-(4-methylpiperazin-1-yl)phenyl)amino)pyrido[2,3-*d*]pyrimidin-7(8*H*)-one (**4**) (31.0 mg, 0.012 mmol, 39% yield) as an off white lyophilised solid. MS (ESI+) *m/z* calcd for $C_{24}H_{31}N_6O^+$ [M + H]$^+$ 419.3, found 419.4. UPLC analysis (method D), 3.58 min, >98% purity. ^{1}H NMR (300 MHz, DMSO-*d6*) δ 9.73 (s, 1H), 8.77 (s, 1H), 7.57 – 7.47 (m, 2H), 6.98 – 6.87 (m, 2H), 6.15 (d, *J* = 1.3 Hz, 1H), 5.89 – 5.75 (m, 1H), 3.09 (t, *J* = 5.0 Hz, 4H), 2.46 (t, *J* = 5.0 Hz, 4H), 2.36 (d, *J* = 1.2 Hz, 3H), 2.26-2.19 (m, 5H), 1.94 – 1.80 (m, 2H), 1.79 – 1.66 (m, 2H), 1.57 (t, *J* = 5.8 Hz, 2H). Data consistent with literature.[3]

8-Cyclopentyl-2-((4-(4-isopropylpiperazin-1-yl)phenyl)amino)-5-methylpyrido[2,3-*d*]pyrimidin-7(8*H*)-one (5)

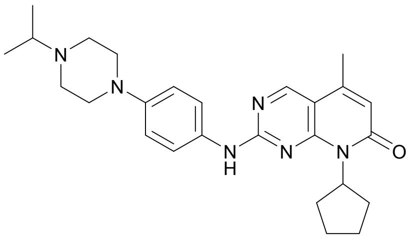

4-(4-Isopropylpiperazin-1-yl)aniline (**35**) (83.2 mg, 0.380 mmol), 2-chloro-8-cyclopentyl-5-methylpyrido[2,3-*d*]pyrimidin-7(8*H*)-one (**36**) (50.0 mg, 0.190 mmol), tripotassium phosphate (88.5 mg, 0.380 mmol), tris(dibenzylideneacetone)dipalladium(0) chloroform adduct (9.8 mg, 0.010 mmol) and 4,5-bis(diphenylphosphino)-9,9-dimethylxanthene (11.0 mg, 0.020 mmol) were sealed in a MW vial before 1,4-dioxane (0.95 mL) was added. The reaction mixture was degassed with N_2 for 5 mins before being heated to 60 °C for 64 hrs. The crude material was purified via preparative HPLC (gradient elution 40 to 80% MeCN in H_2O with 0.1% NH_3) to yield 8-cyclopentyl-2-((4-(4-isopropylpiperazin-1-yl)phenyl)amino)-5-methylpyrido[2,3-*d*]pyrimidin-7(8*H*)-one (**5**) (65 mg, 0.146mmol, 77% yield). MS (ESI+) *m/z* calcd for $C_{26}H_{35}N_6O^+$ [M + H]$^+$ 447.3, found 447.2. UPLC analysis (method D), 3.96 min, >95% purity. ^{1}H NMR (300 MHz, DMSO-d_6) δ 9.74 (s, 1H), 8.76 (s, 1H), 7.50 (d, *J* = 8.7 Hz, 2H), 6.91 (d, *J* = 9.0 Hz, 2H), 6.15 (d, *J* = 1.3 Hz, 1H), 5.81 (s, 1H), 3.07 (t, *J* = 5.0 Hz, 4H), 2.67 (quint, *J* = 6.5 Hz, 1H), 2.57 (t, *J* = 5.0 Hz, 4H), 2.35 (d, *J* = 1.1 Hz, 3H), 2.31 – 2.15 (m, 2H), 1.86 (s, 2H), 1.81 – 1.66 (m, 2H), 1.66 – 1.51 (m, 2H), 1.01 (d, *J* = 6.5 Hz, 6H).

2-Chloro-*N*-cyclopentyl-5-methoxypyrimidin-4-amine (40)

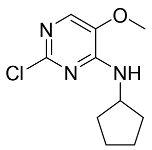

2,4-Dichloro-5-methoxypyrimidine (**38**) (1.00 g, 5.59 mmol) was taken up in THF (28 mL) to which cyclopentylamine (**39**) (0.61 mL, 6.15 mmol) and triethylamine (2.0 mL, 14 mmol) were added. The reaction was stirred at 25 °C for 18 hrs. The mixture was concentrated in vacuo and the resultant residue was taken up in EtOAc (100 mL) and washed with sat. aq. $NaHCO_3$ (2 × 100 mL) and brine (100 mL). The organic layer was dried over $MgSO_4$ and concentrated in vacuo to give 2-chloro-*N*-cyclopentyl-5-methoxypyrimidin-4-amine (**40**) (1008 mg, 4.43 mmol, 79% yield). MS (ESI+) *m/z* calcd for $C_{10}H_{15}ClN_3O^+$ $[M + H]^+$ 228.1, found 228.2. UPLC analysis (method A), 2.92 min, 94% purity. ^1H NMR (300 MHz, $CDCl_3$) δ 7.50 (s, 1H), 5.39 (d, *J* = 7.6 Hz, 1H), 4.43 (sex, *J* = 7.1 Hz, 1H), 3.88 (s, 3H), 2.21 – 2.01 (m, 2H), 1.82 – 1.61 (m, 4H), 1.56 – 1.38 (m, 2H).

2-Chloro-4-(cyclopentylamino)pyrimidin-5-ol (41)

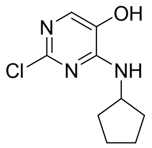

To a solution of 2-chloro-*N*-cyclopentyl-5-methoxypyrimidin-4-amine (**40**) (997 mg, 4.38 mmol) in DCM (22 mL) was added boron tribromide (1.0M in DCM, 22 mL, 22 mmol) and the reaction mixture was stirred at rt for 27 hrs. Upon completion the reaction was quenched with the addition of MeOH (10 mL), diluted with DCM (50 mL), washed with H_2O (50 mL), then with sat. aq. $NaHCO_3$ (50 mL) and brine (50 mL). Then the organics were dried (Na_2SO_4) and concentrated in vacuo to yield 2-chloro-4-(cyclopentylamino)pyrimidin-5-ol (**41**) (870 mg, 4.07 mmol, 93% yield). MS (ESI+) *m/z* calcd for $C_9H_{13}ClN_3O^+$ $[M + H]^+$ 214.1, found 214.2. UPLC analysis (method A), 1.81 min, 91% purity. ^1H NMR (300 MHz, $CDCl_3$) δ 7.35 (s, 1H), 5.83 (d, *J* = 7.8 Hz, 1H), 4.45 (sex, *J* = 7.0 Hz, 1H), 2.22 – 2.05 (m, 2H), 1.87 – 1.40 (m, 6H).

2-Chloro-8-cyclopentyl-6,6-dimethyl-6*H*-pyrimido[5,4-*b*][1,4]oxazin-7(8*H*)-one (42)

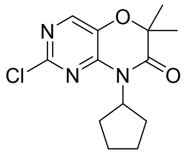

To a solution of 2-chloro-4-(cyclopentylamino)pyrimidin-5-ol (**41**) (870 mg, 4.07 mmol) and potassium carbonate (1.69 g, 12.2 mmol) in MeCN (24 mL) under N_2 was added methyl 2-bromo-2-methylpropanoate (0.68 mL, 5.3 mmol) and the reaction mixture was heated at 80 °C for 16 hrs. Upon completion the reaction mixture was diluted with EtOAc (50 mL), washed with sat. aq. $NaHCO_3$ (2 × 50 mL) and brine (50 mL), then dried (Na_2SO_4), filtered and concentrated in vacuo. Purification via silica gel chromatography (gradient elution 2 to 25% EtOAc in petroleum ether) yielded 2-chloro-8-cyclopentyl-6,6-dimethyl-6*H*-pyrimido[5,4-*b*][1,4]oxazin-7(8*H*)-one (**42**) (815 mg, 2.89 mmol, 71% yield) as a white solid. MS (ESI+) *m/z* calcd for $C_{13}H_{17}ClN_3O_2^+$ [M + H]$^+$ 282.1, found 282.2. UPLC analysis (method A), 3.42 min, >95% purity. ^{1}H NMR (300 MHz, $CDCl_3$) δ 8.10 (s, 1H), 5.45 – 5.28 (m, 1H), 2.16 – 1.98 (m, 4H), 1.98 – 1.83 (m, 2H), 1.71 – 1.61 (m, 2H), 1.55 (s, 6H).

8-Cyclopentyl-6,6-dimethyl-2-((4-(4-methylpiperazin-1-yl)phenyl)amino)-6*H*-pyrimido[5,4-*b*][1,4]oxazin-7(8*H*)-one (6)

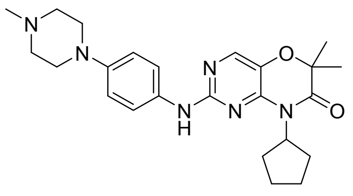

2-Chloro-8-cyclopentyl-6,6-dimethyl-6*H*-pyrimido[5,4-*b*][1,4]oxazin-7(8*H*)-one (**42**) (45.0 mg, 0.160 mmol) and 4-(4-methylpiperazino)aniline (39.7 mg, 0.208 mmol) were reacted according to general procedure **A**. Purification via preparatory HPLC (0.1% NH_3 in MeCN/Water; 30-80%) yielded 8-cyclopentyl-6,6-dimethyl-2-((4-(4-methylpiperazin-1-yl)phenyl)amino)-6*H*-pyrimido[5,4-*b*][1,4]oxazin-7(8*H*)-one (**6**) (9.6 mg, 0.022 mmol, 14% yield) as an off white solid. MS (ESI+) *m/z* calcd for $C_{24}H_{33}N_6O_2^+$ [M + H]$^+$ 437.3, found 437.4. UPLC analysis (method D), 4.29 min, >98% purity. ^{1}H NMR (300 MHz, CDCl3) δ 7.96 (s, 1H), 7.48 – 7.37 (m, 2H), 7.01 – 6.89 (m, 2H), 6.73 (s, 1H), 5.32 (quint, *J* = 8.7 Hz, 1H), 3.24 – 3.15 (m, 4H), 2.66 – 2.57 (m, 4H), 2.38 (s, 3H), 2.22 – 2.07 (m, 2H), 2.00 – 1.82 (m, 4H), 1.67 – 1.62 (m, 2H), 1.51 (s, 6H).

7-Cyclopentyl-2-((4-(4-isopropylpiperazin-1-yl)phenyl)amino)-5,7-dihydro-6*H*-pyrrolo[2,3-*d*]pyrimidin-6-one (7)

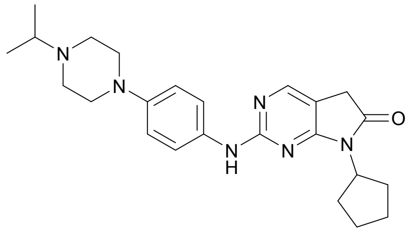

2-Chloro-7-cyclopentyl-5*H*-pyrrolo[2,3-*d*]pyrimidin-6-one (**43**) was prepared as reported previously.[4] Then **43** (50.0 mg, 0.210 mmol), 4-(4-isopropylpiperazin-1-yl)aniline (**35**) (60.4 mg, 0.280 mmol) and *p*-toluenesulfonic acid monohydrate (160 mg, 0.840 mmol) were sealed in a MW vial. 2-BuOH (2.1 mL) was added and the reaction was heated under microwave irradiation at 125 °C for 1 hr. After this time the reaction was stopped and purified *via* preparatory HPLC (gradient elution 50 to 90% MeCN in H_2O with 0.1% NH_3) to yield 7-cyclopentyl-2-((4-(4-isopropylpiperazin-1-yl)phenyl)amino)-5,7-dihydro-6*H*-pyrrolo[2,3-*d*]pyrimidin-6-one (**7**) (25 mg, 0.059 mmol, 28% yield). MS (ESI+) *m/z* calcd for $C_{23}H_{29}N_6O^+$ $[M + H]^+$ 421.3, found 421.3. UPLC analysis, unstable to both method C and D. ^{1}H NMR (300 MHz, DMSO-*d6*) δ 9.17 (s, 1H), 8.00 (s, 1H), 7.54 (d, *J* = 9.0 Hz, 2H), 6.87 (d, *J* = 9.1 Hz, 2H), 4.66 (quint, *J* = 8.7 Hz, 1H), 3.49 (s, 2H), 3.11 – 2.97 (m, 4H), 2.76 – 2.63 (m, 1H), 2.63 – 2.55 (m, 4H), 2.26 – 2.08 (m, 2H), 1.98 – 1.85 (m, 2H), 1.85 – 1.72 (m, 2H), 1.68 – 1.53 (m, 2H), 1.01 (d, *J* = 6.5 Hz, 6H).

2-Chloro-7-cyclopentyl-pyrrolo[2,3-*d*]pyrimidine (46)

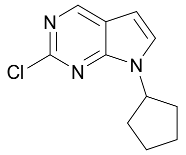

Sodium hydride (26.1 mg, 0.650 mmol) was sealed in a flask and MeCN (0.50 mL) was added followed by a solution of 2-chloro-7*H*-pyrrolo[2,3-*d*]pyrimidine (**44**) (50.0 mg, 0.330 mmol) in DMF (1.60 mL). Iodocyclopentane (**45**) (0.045 mL, 0.0391 mmol) in DMF (0.5 mL) was then added and the reaction was stirred at rt for 18 hrs. After this time the reaction was quenched with sat. aq. NH_4Cl and extracted with EtOAc (x2). The organic layer was washed with 10% LiCl (aq), dried (Na_2SO_4) and concentrated *in vacuo*. Purification via silica gel chromatography (gradient elution 0 to 50% EtOAc in petroleum ether) yielded 2-chloro-7-cyclopentyl-pyrrolo[2,3-*d*]pyrimidine (**46**) (55 mg, 0.248 mmol, 76% yield) as a pale yellow oil. MS (ESI+) *m/z* calcd for $C_{11}H_{13}ClN_3^+$ $[M + H]^+$ 222.1, found 222.2. UPLC analysis (method B), 3.07 min, >95% purity. ^{1}H NMR (300 MHz, CDCl3) δ 8.78 (s, 1H), 7.29 (d, *J* = 3.7 Hz, 1H), 6.57 (d, *J* = 3.7 Hz, 1H), 5.32 – 5.08 (m, 1H), 2.37 – 2.16 (m, 2H), 1.98 – 1.70 (m, 6H).

7-Cyclopentyl-N-(4-(4-isopropylpiperazin-1-yl)phenyl)-7*H*-pyrrolo[2,3-*d*]pyrimidin-2-amine (8)

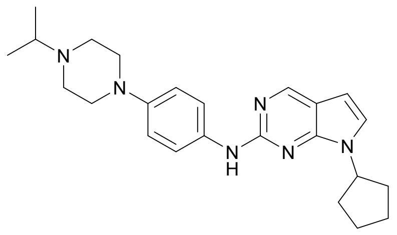

2-Chloro-7-cyclopentyl-pyrrolo[2,3-*d*]pyrimidine (**46**) (60.0 mg, 0.270 mmol) and 4-(4-isopropylpiperazin-1-yl)aniline (**35**) (71.2 mg, 0.32 mmol), tris(dibenzylideneacetone)dipalladium(0) chloroform adduct (14.0 mg, 0.010 mmol), 4,5-bis(diphenylphosphino)-9,9-dimethylxanthene (15.7 mg, 0.030 mmol) and caesium carbonate (176 mg, 0.540 mmol) were sealed in a MW vial and the mixture was taken up in 1,4-dioxane (2.7 mL) before degassing with N_2 for 5 mins. The reaction mixture was then heated on a stirrer hotplate at 105 °C for 2 hrs. After this time the reaction mixture was cooled and loaded onto an SCX-II cartridge, washed with DCM and MeOH, then eluted with 0.5 M NH_3 in MeOH and concentrated in vacuo. The crude material taken up in DMSO and purified by preparatory HPLC (gradient elution 40 to 80% MeCN in H_2O with 0.1% NH_3) to yield 7-cyclopentyl-*N*-(4-(4-isopropylpiperazin-1-yl)phenyl)-7*H*-pyrrolo[2,3-*d*]pyrimidin-2-amine (**8**) (72 mg, 0.178 mmol, 66% yield). MS (ESI+) *m/z* calcd for $C_{24}H_{33}N_6^+$ $[M + H]^+$ 405.3, found 405.3. UPLC analysis (method D), 3.26 min, >98% purity. ^1H NMR (300 MHz, DMSO-*d*6) δ 9.08 (s, 1H), 8.60 (s, 1H), 7.67 (t, *J* = 6.1 Hz, 2H), 7.27 (d, *J* = 3.7 Hz, 1H), 6.88 (t, *J* = 6.2 Hz, 2H), 6.39 (d, *J* = 3.6 Hz, 1H), 5.04 – 4.88 (m, 1H), 3.09 – 2.96 (m, 4H), 2.65 (dt, *J* = 13.0, 6.5 Hz, 1H), 2.60 – 2.54 (m, 4H), 2.18 – 2.08 (m, 2H), 2.00 – 1.80 (m, 4H), 1.80 – 1.62 (m, 2H), 1.00 (d, *J* = 6.5 Hz, 6H).

Table 2 Compounds

***tert*-Butyl 3-(4-nitrophenyl)-3,6-diazabicyclo[3.1.1]heptane-6-carboxylate (49)**

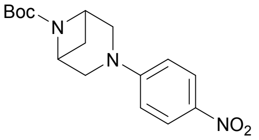

To a stirred solution of 1-fluoro-4-nitrobenzene (**47**) (150 mg, 1.06 mmol) in DMF (4.0 mL) was added *tert*-butyl 3,6-diazabicyclo[3.1.1]heptane-6-carboxylate (**48**) (211 mg, 1.06 mmol), followed by sodium carbonate (0.23 g, 2.13 mmol). The reaction mixture was heated at 90 °C for 18 hrs. After this time the reaction mixture was diluted with EtOAc and washed with sat. aq. $NaHCO_3$. The aqueous fraction was further extracted with EtOAc (x2) and combined organic fractions were dried (Na_2SO_4) and concentrated in vacuo. Purification via silica gel chromatography (gradient elution 0 to 50% EtOAc in petroleum ether) yielded *tert*-butyl 3-(4-nitrophenyl)-3,6-diazabicyclo[3.1.1]heptane-6-carboxylate (**49**) (257 mg, 0.81 mmol, 76%) as a yellow solid. MS (ESI+) *m/z* calcd for $C_{16}H_{22}N_3O_4^+$ $[M + H]^+$ 320.2, found 320.3. UPLC analysis (method A), 3.11 min, >98% purity. ^1H NMR (300 MHz, $CDCl_3$) δ 8.26 – 8.13 (m, 2H), 6.75 – 6.64 (m, 2H), 4.34 (d, *J* = 5.7 Hz, 2H), 4.18 – 3.92 (m, 2H), 3.40 (d, *J* = 11.0 Hz, 2H), 2.73 (dd, *J* = 15.1, 6.4 Hz, 1H), 1.52 (d, *J* = 8.9 Hz, 1H), 1.37 (s, 9H).

6-Methyl-3-(4-nitrophenyl)-3,6-diazabicyclo[3.1.1]heptane (50)

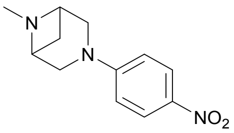

To a stirred solution of *tert*-butyl 3-(4-nitrophenyl)-3,6-diazabicyclo[3.1.1]heptane-6-carboxylate (**49**) (1.45 g, 4.54 mmol) in DCM (45 mL) was added trifluoroacetic acid (3.48 mL, 45.4 mmol) and stirred at rt for 16 hrs. The reaction was stopped, and the reaction mixture concentrated in vacuo with toluene azeotroping (×2). The material was taken up in DCE (15.2 mL) and acetic acid (0.35 mL, 6.2 mmol) and stirred at rt before formaldehyde (2.31 mL, 31.0 mmol) was added, followed by portion wise addition of sodium triacetoxyborohydride (1.31 g, 6.20 mmol). The reaction mixture was allowed to stir for 16 hrs before the reaction was quenched with sat. aq. $NaHCO_3$ and H_2O. The aqueous layer was extracted with DCM:IPA (4:1) (×3) and the combined organic layers were washed with brine, separated, dried (Na_2SO_4) and filtered. The solvent was removed in vacuo and the crude material purified via silica gel chromatography (gradient elution 0 to 20% MeOH in DCM) to yield 6-methyl-3-(4-nitrophenyl)-3,6-diazabicyclo[3.1.1]heptane (**50**) (500 mg, 2.14 mmol, 69% yield) as a yellow solid. MS (ESI+) *m/z* calcd for $C_{12}H_{16}N_3O_2^+$ $[M + H]^+$ 234.1, found 234.3. B-9 UPLC analysis (method A), 2.57 min, >98%

purity. ^{1}H NMR (300 MHz, $CDCl_3$) δ 8.25 – 8.13 (m, 2H), 6.78 – 6.63 (m, 2H), 3.84 (d, *J* = 5.9 Hz, 2H), 3.70 (d, *J* = 11.9 Hz, 2H), 3.47 (d, *J* = 11.7 Hz, 2H), 2.76 (dd, *J* = 14.9, 6.2 Hz, 1H), 2.22 (s, 3H), 1.65 (d, *J* = 9.0 Hz, 1H).

4-(6-Methyl-3,6-diazabicyclo[3.1.1]heptan-3-yl)aniline (51)

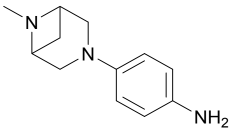

A flask containing 6-methyl-3-(4-nitrophenyl)-3,6-diazabicyclo[3.1.1]heptane (**50**) (500 mg, 2.14 mmol) was evacuated under vacuum and filled with N_2 three times. Methanol (10 mL) was added and the reaction mixture stirred, before Pd/C (10% w/w, 23 mg, 0.210 mmol) was added and the flask evacuated again before being filled with H_2 *via* a balloon and left to stir for 16 hrs. After this time the reaction was stopped, passed through celite, washed with methanol and concentrated *in vacuo* to give 4-(6-methyl-3,6-diazabicyclo[3.1.1]heptan-3-yl)aniline (**51**) (420 mg, 2.07 mmol, 96%). The material was carried forward without further purification. MS (ESI+) *m/z* calcd for $C_{12}H_{18}N_3^+$ $[M + H]^+$ 204.1, found 204.0. UPLC analysis (method A), 0.52 min, >95% purity. ^{1}H NMR (300 MHz, $CDCl_3$) δ 6.80 – 6.70 (m, 2H), 6.63 – 6.56 (m, 2H), 3.74 (d, *J* = 5.8 Hz, 2H), 3.50 (d, *J* = 10.9 Hz, 2H), 3.31 (d, *J* = 10.6 Hz, 2H), 2.65 (dd, *J* = 14.2, 6.2 Hz, 1H), 2.15 (s, 3H), 1.67 (d, *J* = 8.5 Hz, 1H).

Caution! *Hydrogen is classified as a GHS Flammable Gas, Category 1. Hydrogen and the hydrogen balloon were handled using standard procedures.*

8-Cyclopentyl-5-methyl-2-((4-(6-methyl-3,6-diazabicyclo[3.1.1]heptan-3-yl)phenyl)amino)pyrido[2,3-*d*]pyrimidin-7(8*H*)-one (9)

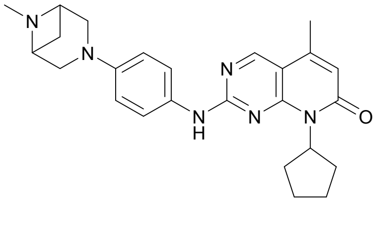

To a solution of tris(dibenzylideneacetone)dipalladium(0) chloroform adduct (9.8 mg, 0.010 mmol) and 4,5-bis(diphenylphosphino)-9,9-dimethylxanthene (11.0 mg, 0.020 mmol) in 1,4-dioxane (1.9 mL) was added 4-(6-methyl-3,6-diazabicyclo[3.1.1]heptan-3-yl)aniline (**51**) (57.8 mg, 0.280 mmol), 2-chloro-8-cyclopentyl-5-methylpyrido[2,3-*d*]pyrimidin-7(8*H*)-one (**36**) (50.0 mg, 0.190 mmol) and potassium phosphate (88.5 mg, 0.380 mmol). The reaction mixture was degassed with N_2 for 5 mins before heating

at 50 °C for 20 hrs. After this time the reaction mixture was cooled to rt, loaded onto an SCX-II cartridge, washed with DCM and MeOH, then eluted with 0.5 M NH_3 in MeOH. The MeOH wash and methanolic ammonia eluent were concentrated in vacuo. The crude residue was taken up in DMSO and purified by preparatory HPLC (gradient elution 40 to 80% MeCN in H_2O with 0.1% NH_3)., followed by further purification via silica gel chromatography (gradient elution 0 to 20% MeOH in DCM) yielded 8-cyclopentyl-5-methyl-2-((4-(6-methyl-3,6-diazabicyclo[3.1.1]heptan-3-yl)phenyl)amino)pyrido[2,3-*d*]pyrimidin-7(8*H*)-one (**9**) (17.1 mg, 0.040 mmol, 21% yield) as a yellow solid. MS (ESI+) *m/z* calcd for $C_{25}H_{31}N_6O^+$ [M + H]$^+$ 431.3, found 431.4. UPLC analysis (method D), 3.69 min, >95% purity. HRMS (ESI+) *m/z* calcd for $C_{25}H_{31}N_6O^+$ [M + H]$^+$ 431.2559, found 431.2543. ^{1}H NMR (300 MHz, $CDCl_3$) δ 8.61 (s, 1H), 7.50 – 7.39 (m, 2H), 7.04 (s, 1H), 6.82 – 6.72 (m, 2H), 6.24 (d, *J* = 1.2 Hz, 1H), 5.85 (quint, *J* = 8.8 Hz, 1H), 3.74 (d, *J* = 5.9 Hz, 2H), 3.59 (d, *J* = 11.0 Hz, 2H), 3.36 (d, *J* = 10.9 Hz, 2H), 2.65 (q, *J* = 6.7 Hz, 1H), 2.41 – 2.25 (m, 5H), 2.18 (s, 3H), 1.99 – 1.76 (m, 4H), 1.73 – 1.66 (m, 2H). ^{13}C NMR (126 MHz, DMSO-*d*6, MeOD) δ 164.8, 160.6, 157.5, 156.7, 147.0, 146.0, 129.5, 124.1, 117.4, 110.5, 107.7, 59.8, 54.0, 44.6, 30.5, 28.5, 26.0, 17.0.

Ethyl 4-(cyclopentylamino)-2-(methylthio)pyrimidine-5-carboxylate (58)

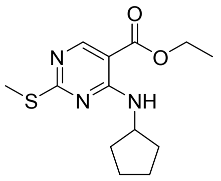

Ethyl 4-chloro-2-(methylthio)pyrimidine-5-carboxylate (**57**) (1.00 g, 4.30 mmol) was taken up in THF (21.5 mL) to which cyclopentylamine (**39**) (0.47 mL, 4.73 mmol) and triethylamine (1.80 mL, 12.9 mmol) were added. The reaction mixture was stirred at 25 °C for 18 hrs. The mixture was then concentrated in vacuo and the resultant residue was taken up in EtOAc (100 mL), washed with sat. aq. $NaHCO_3$ (2 × 100 mL) and brine (100 mL). The organic layer was dried over $MgSO_4$ and concentrated in vacuo to give ethyl 4-(cyclopentylamino)-2-(methylthio)pyrimidine-5-carboxylate (**58**) (642 mg, 2.28 mmol, 53% yield) as a pale brown oil. MS (ESI+) *m/z* calcd for $C_{13}H_{20}N_3O_2S^+$ [M + H]$^+$ 282.1, found 282.2. UPLC analysis (method A), 3.61 min, 94% purity. ^{1}H NMR (300 MHz, $CDCl_3$) δ 8.62 (s, 1H), 8.26 (d, *J* = 6.7 Hz, 1H), 4.51 (sex, *J* = 6.8 Hz, 1H), 4.33 (q, *J* = 7.1 Hz, 2H), 2.55 (s, 3H), 2.16 – 2.03 (m, 2H), 1.81 – 1.49 (m, 6H), 1.39 (t, *J* = 7.1 Hz, 3H).

(4-(Cyclopentylamino)-2-(methylthio)pyrimidin-5-yl)methanol (59)

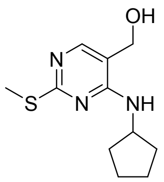

To a solution of ethyl 4-(cyclopentylamino)-2-(methylthio)pyrimidine-5-carboxylate (**58**) (640 mg, 2.27 mmol) in THF (3.0 mL) at 0 °C was added lithium aluminium hydride (1M in THF, 3.4 mL, 3.4 mmol) dropwise over 5 min. The reaction was then warmed to rt and stirred for a further 24 hrs. Upon completion the reaction was quenched with H_2O (100 ml), then extracted with EtOAc (2 × 50 mL). The combined organics were washed with sat. aq. $NaHCO_3$ (100 mL) and brine (100 mL), then dried (Na_2SO_4) and concentrated in vacuo to give (4-(cyclopentylamino)-2-(methylthio)pyrimidin-5-yl)methanol (**59**) (484 mg, 2.02 mmol, 89% yield) as a yellow solid. MS (ESI+) *m/z* calcd for $C_{11}H_{18}N_3OS^+$ $[M + H]^+$ 240.1, found 240.2. UPLC analysis (method A), 2.78 min, >98% purity. ^1H NMR (300 MHz, $CDCl_3$) δ 7.73 (s, 1H), 5.83 (br d, *J* = 6.8 Hz, 1H), 4.52 (d, *J* = 0.6 Hz, 2H), 4.45 (quint, *J* = 6.8 Hz, 1H), 2.53 (s, 3H), 2.17 – 2.02 (m, 2H), 1.84 – 1.58 (m, 6H), 1.57 – 1.42 (m, 1H).

4-(Cyclopentylamino)-2-(methylthio)pyrimidine-5-carbaldehyde (60)

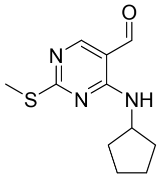

To a solution of (4-(cyclopentylamino)-2-(methylthio)pyrimidin-5-yl)methanol (**59**) (484 mg, 2.02 mmol) in chloroform (20 mL) was added manganese(IV) oxide (1.23 g, 14.2 mmol) and the reaction was stirred at rt for 17 hrs. Upon completion the reaction mixture was filtered through a pad of celite (eluent $CHCl_3$) and concentrated in vacuo to give 4-(cyclopentylamino)-2-(methylthio)pyrimidine-5-carbaldehyde (**60**) (468 mg, 1.97 mmol, 98% yield) as a pale grey oil. MS (ESI+) *m/z* calcd for $C_{11}H_{16}N_3OS^+$ $[M + H]^+$ 238.1, found 238.2. UPLC analysis (method A), 3.19 min, 98% purity. ^1H NMR (300 MHz, $CDCl_3$) δ 9.70 (s, 1H), 8.62 (s, 1H), 8.30 (s, 1H), 4.55 (sex, *J* = 6.8 Hz, 1H), 2.58 (s, 3H), 2.20 – 2.03 (m, 2H), 1.89 – 1.59 (m, 6H).

8-Cyclopentyl-2-(methylthio)-7-oxo-7,8-dihydropyrido[2,3-*d*]pyrimidine-6-carbonitrile (61)

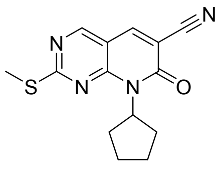

To a solution of 4-(cyclopentylamino)-2-(methylthio)pyrimidine-5-carbaldehyde (**60**) (468 mg, 1.97 mmol) and cyanoacetic acid (201 mg, 2.37 mmol) in acetic acid (20 mL) was added benzylamine (43 µL, 0.39 mmol) and the reaction mixture was stirred at 120 °C for 18 hrs. Upon completion the reaction mixture was cooled to rt and the resulting precipitate collected via filtration to give 8-cyclopentyl-2-(methylthio)-7-oxo-7,8-dihydropyrido[2,3-*d*]pyrimidine-6-carbonitrile (**61**) (133 mg, 0.464 mmol, 24% yield) as an off white solid. MS (ESI+) *m/z* calcd for $C_{14}H_{15}N_4OS^+$ $[M + H]^+$ 287.1, found 287.2. UPLC analysis (method A), 3.15 min, >98% purity. ^1H NMR (300 MHz, $CDCl_3$) δ 8.70 (s, 1H), 8.11 (s, 1H), 5.98 (quint, *J* = 8.7 Hz, 1H), 2.67 (s, 3H), 2.31 (dt, *J* = 19.4, 7.7 Hz, 2H), 2.19 – 2.06 (m, 2H), 2.00 – 1.85 (m, 2H), 1.79 – 1.65 (m, 2H).

8-Cyclopentyl-2-(methylsulfinyl)-7-oxo-7,8-dihydropyrido[2,3-*d*]pyrimidine-6-carbonitrile (62)

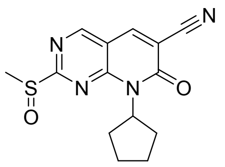

To a solution of 8-cyclopentyl-2-(methylthio)-7-oxo-7,8-dihydropyrido[2,3-*d*]pyrimidine-6-carbonitrile (**61**) (133 mg, 0.464 mmol) in DCM (4.6 mL) was added *m*CPBA (100 mg, 0.581 mmol) and the reaction mixture was stirred at rt for 2 hrs. Upon completion the reaction mixture was diluted with DCM (25 mL), washed with sat. aq. $NaHCO_3$ (25 mL) and brine (25 mL), then dried (hydrophobic frit) and concentrated in vacuo to give 8-cyclopentyl-2-(methylsulfinyl)-7-oxo-7,8-dihydropyrido[2,3-*d*]pyrimidine-6-carbonitrile **62** (134 mg, 0.443 mmol, 95% yield) as an orange solid which was used without further purification due to instability. MS (ESI+) *m/z* calcd for $C_{14}H_{15}N_4O_2S^+$ $[M + H]^+$ 303.1, found 303.2. UPLC analysis (method A), 2.44 min, 73% purity.

8-Cyclopentyl-2-((4-(6-methyl-3,6-diazabicyclo[3.1.1]heptan-3-yl)phenyl)amino)-7-oxo-7,8-dihydropyrido[2,3-*d*]pyrimidine-6-carbonitrile (10)

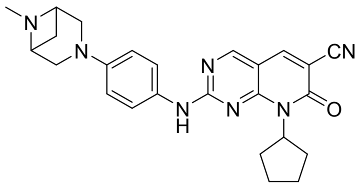

8-Cyclopentyl-2-(methylsulfinyl)-7-oxo-7,8-dihydropyrido[2,3-*d*]pyrimidine-6-carbonitrile (**62**) (90.0 mg, 0.30 mmol) and 4-(6-methyl-3,6-diazabicyclo[3.1.1]heptan-3-yl)aniline (**51**) (78.0 mg, 0.39 mmol) were reacted according to general procedure **B**. Purification via preparatory HPLC (0.1%

NH_3 in MeCN/Water; 40-80%) yielded 8-cyclopentyl-2-((4-(6-methyl-3,6-diazabicyclo[3.1.1]heptan-3-yl)phenyl)amino)-7-oxo-7,8-dihydropyrido[2,3-*d*]pyrimidine-6-carbonitrile (**10**) (37.0 mg, 0.084 mmol, 28% yield) as an orange lyophilised solid. MS (ESI+) *m/z* calcd for $C_{25}H_{28}N_7O^+$ [M + H]$^+$ 442.2, found 442.4. UPLC analysis (method D), 3.86 min, >98% purity. HRMS (ESI+) *m/z* calcd for $C_{25}H_{28}N_7O^+$ [M + H]$^+$ 442.2349, found 442.2355. ^{1}H NMR (500 MHz, DMSO-*d6*) δ 10.40 – 9.98 (m, 1H), 8.74 (s, 1H), 8.48 (s, 1H), 7.46 (d, *J* = 8.8 Hz, 2H), 6.72 (d, *J* = 8.8 Hz, 2H), 6.00 – 5.59 (m, 1H), 3.56 (d, *J* = 5.9 Hz, 2H), 3.42 (d, *J* = 11.1 Hz, 2H), 3.27 – 3.23 (m, 2H), 2.41 (q, *J* = 7.1 Hz, 1H), 2.24 – 2.12 (m, 2H), 1.97 (s, 3H), 1.89 – 1.65 (m, 4H), 1.62 – 1.44 (m, 3H). ^{13}C NMR (126 MHz, DMSO-*d6*) δ 161.6, 160.7, 160.2, 157.0, 145.9, 127.7, 123.7, 123.4, 116.8, 110.0, 105.4, 99.7, 58.7, 54.1, 44.2, 31.8, 30.2, 27.7, 25.3.

8-Cyclopentyl-2-((3-(4-methylpiperazin-1-yl)phenyl)amino)-7-oxo-7,8-dihydropyrido[2,3-*d*]pyrimidine-6-carbonitrile (11)

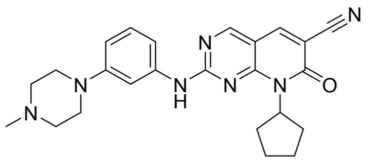

8-Cyclopentyl-2-(methylsulfinyl)-7-oxo-7,8-dihydropyrido[2,3-*d*]pyrimidine-6-carbonitrile (**62**) (35.0 mg, 0.12 mmol) and 3-(4-methylpiperazin-1-yl)aniline (26.6 mg, 0.14 mmol) were reacted according to general procedure **B**. Purification via preparatory HPLC (0.1% NH_3 in MeCN/Water; 30-68%) yielded 8-cyclopentyl-2-((3-(4-methylpiperazin-1-yl)phenyl)amino)-7-oxo-7,8-dihydropyrido[2,3-*d*]pyrimidine-6-carbonitrile (**11**) (13.5 mg, 0.031 mmol, 27% yield) as a yellow lyophilised solid. MS (ESI+) *m/z* calcd for $C_{24}H_{28}N_7O^+$ [M + H]$^+$ 430.2, found 430.5. UPLC analysis (method C), 5.42 min, >98% purity. ^{1}H NMR (300 MHz, DMSO-*d6*) δ 10.41 (s, 1H), 8.84 (s, 1H), 8.57 (s, 1H), 7.39 (s, 1H), 7.20 (t, *J* = 8.1 Hz, 1H), 7.03 (s, 1H), 6.73 (d, *J* = 7.2 Hz, 1H), 5.95 – 5.83 (m, 1H), 3.13 (t, *J* = 5.0 Hz, 4H), 2.46 (t, *J* = 5.0 Hz, 4H), 2.23 (s, 3H), 2.21 – 2.11 (m, 2H), 2.00 – 1.88 (m, 2H), 1.84 – 1.73 (m, 2H), 1.63 – 1.51 (m, 2H).

8-Cyclopentyl-2-((2-methoxy-4-(4-methylpiperazin-1-yl)phenyl)amino)-5-methylpyrido[2,3-d]pyrimidin-7(8*H*)-one (12)

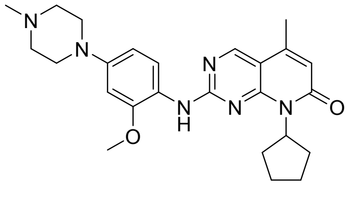

2-Chloro-8-cyclopentyl-5-methylpyrido[2,3-*d*]pyrimidin-7(8*H*)-one (**36**) (50.0 mg, 0.190 mmol) and 2-methoxy-4-(4-methyl-1-piperazinyl)aniline (**52**) (54.5 mg, 0.246mmol) were reacted according to general procedure **A**. Purification via preparatory HPLC (0.1% NH_3 in MeCN/Water; 30-68%) yielded 8-cyclopentyl-2-((2-methoxy-4-(4-methylpiperazin-1-yl)phenyl)amino)-5-methylpyrido[2,3-*d*]pyrimidin-7(8*H*)-one (**12**) (27.7 mg, 0.062 mmol, 33% yield) as a lyophilised pale yellow solid. MS (ESI+) *m/z* calcd for $C_{25}H_{33}N_6O_2^+$ [M + H]$^+$ 449.3, found 449.3. UPLC analysis (method D), 3.83 min, >98% purity. ^{1}H NMR (300 MHz, DMSO-*d6*) δ 8.79 – 8.62 (m, 2H), 7.31 (d, *J* = 8.6 Hz, 1H), 6.63 (d, *J* = 2.5 Hz, 1H), 6.50 (dd, *J* = 8.7, 2.5 Hz, 1H), 6.13 (d, *J* = 1.2 Hz, 1H), 5.61 (s, 1H), 3.75 (s, 3H), 3.15 (t, *J* = 5.0 Hz, 4H), 2.47 (t, *J* = 5.0 Hz, 4H), 2.34 (d, *J* = 1.2 Hz, 3H), 2.23 (s, 3H), 2.15 (s, 2H), 1.57 (s, 4H), 1.41 (s, 2H).

***tert*-Butyl 4-(6-((8-cyclopentyl-5-methyl-7-oxo-7,8-dihydropyrido[2,3-*d*]pyrimidin-2-yl)amino)pyridin-3-yl)piperazine-1-carboxylate (56)**

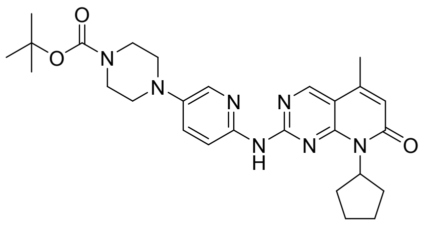

To a solution of *tert*-butyl 4-(6-aminopyridin-3-yl)piperazine-1-carboxylate (**53**) (111 mg, 0.398 mmol) in toluene (1.9 mL) under N_2 was added lithium bis(trimethylsilyl)amide (1.0 M in toluene, 0.40 mL, 0.40 mmol) and the mixture stirred at rt for 10 min. Then a solution of 2-chloro-8-cyclopentyl-5-methylpyrido[2,3-*d*]pyrimidin-7(8*H*)-one (**36**) (50.0 mg, 0.190 mmol) in toluene (1.9 mL) was added and the resulting solution was stirred at rt for 3 hrs. Upon completion the reaction was quenched by the addition of sat. aq. NH_4Cl (25 mL), then extracted with DCM:IPA (4:1 2 x 25 mL) and the combined organics were washed with brine (50 mL) then dried (hydrophobic frit) and concentrated in vacuo. Purification *via* silica gel chromatography (gradient elution 0 to 5% MeOH in DCM) yielded a residue with residual solvent. This was redissolved in EtOAc:IPA (5:1, 100 mL), then washed with 10% LiCl (2 x 100 mL) and brine (100 mL), then concentrated in vacuo to give *tert*-butyl 4-(6-((8-cyclopentyl-5-methyl-7-oxo-7,8-dihydropyrido[2,3-*d*]pyrimidin-2-yl)amino)pyridin-3-yl)piperazine-1-carboxylate (**56**) (86.0 mg, 0.170 mmol, 90% yield). LCMS indicated impurities were still present but due to poor solubility further purification was not attempted and product was carried forward. MS (ESI+) *m/z* calcd for $C_{27}H_{36}N_7O_3^+$ [M + H]$^+$ 506.3, found 506.0. UPLC analysis (method B), 3.06 min, 67% purity.

8-Cyclopentyl-5-methyl-2-((5-(piperazin-1-yl)pyridin-2-yl)amino)pyrido[2,3-*d*]pyrimidin-7(8*H*)-one (13)

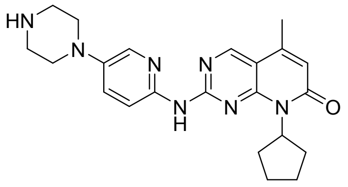

To a solution of *tert*-butyl 4-(6-((8-cyclopentyl-5-methyl-7-oxo-7,8-dihydropyrido[2,3-*d*]pyrimidin-2-yl)amino)pyridin-3-yl)piperazine-1-carboxylate (**56**) (29.0 mg, 0.057 mmol) in DCM (3.2 mL) was added trifluoroacetic acid (0.088 mL, 1.2 mmol) and the reaction mixture was stirred at rt for 4 hr. Upon completion the reaction mixture was concentrated in vacuo. Purification *via* preparatory HPLC (0.1% NH_3 in MeCN/Water; 15-95%) yielded 8-cyclopentyl-5-methyl-2-((5-(piperazin-1-yl)pyridin-2-yl)amino)pyrido[2,3-*d*]pyrimidin-7(8*H*)-one (**13**) (8.6 mg, 0.021 mmol, 37% yield) as a white lyophilised solid. MS (ESI+) *m/z* calcd for $C_{22}H_{28}N_7O^+$ $[M + H]^+$ 406.2, found 406.0. UPLC analysis (method D), 2.96 min, >98% purity. ^{1}H NMR (300 MHz, DMSO-*d6*) δ 9.91 (s, 1H), 8.81 (s, 1H), 8.02 (d, *J* = 2.9 Hz, 1H), 7.86 (d, *J* = 9.0 Hz, 1H), 7.44 (dd, *J* = 9.1, 3.0 Hz, 1H), 6.21 (d, *J* = 1.3 Hz, 1H), 5.80 (quint, *J* = 8.9 Hz, 1H), 3.17 (d, *J* = 5.2 Hz, 1H), 3.11 – 3.01 (m, 4H), 2.89 – 2.80 (m, 4H), 2.38 (d, *J* = 1.1 Hz, 3H), 2.27 – 2.18 (m, 2H), 1.94 – 1.83 (m, 2H), 1.80 – 1.69 (m, 2H), 1.65 – 1.53 (m, 2H).

8-Cyclopentyl-5-methyl-2-((3,4,5-trimethoxyphenyl)amino)pyrido[2,3-*d*]pyrimidin-7(8*H*)-one (15)

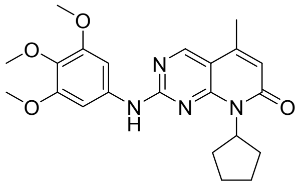

2-Chloro-8-cyclopentyl-5-methylpyrido[2,3-*d*]pyrimidin-7(8*H*)-one (**36**) (50.0 mg, 0.190 mmol) and 3,4,5-trimethoxyaniline (**54**) (45.2 mg, 0.246 mmol) were reacted according to general procedure **A**. Purification via preparatory HPLC (0.1% NH_3 in MeCN/Water; 30-70%) yielded 8-cyclopentyl-5-methyl-2-((3,4,5-trimethoxyphenyl)amino)pyrido[2,3-*d*]pyrimidin-7(8*H*)-one (**15**) (50.0 mg, 0.122 mmol, 64% yield) as a lyophilised white solid. MS (ESI+) *m/z* calcd for $C_{22}H_{27}N_4O_4^+$ $[M + H]^+$ 411.2, found 411.3. UPLC analysis (method D), 5.24 min, >98% purity. ^{1}H NMR (300 MHz, $CDCl_3$) δ 8.66 (s, 1H), 7.19 (s, 1H), 6.92 (s, 2H), 6.29 (d, *J* = 1.3 Hz, 1H), 5.98 (quint, *J* = 8.8 Hz, 1H), 3.92 (s, 6H), 3.87 (s, 3H), 2.41 (d, *J* = 1.2 Hz, 3H), 2.39 – 2.24 (m, 2H), 2.11 – 1.96 (m, 2H), 1.95 – 1.79 (m, 2H), 1.68 – 1.60 (m, 2H).

8-Cyclopentyl-7-oxo-2-((3,4,5-trimethoxyphenyl)amino)-7,8-dihydropyrido[2,3-*d*]pyrimidine-6-carbonitrile (16)

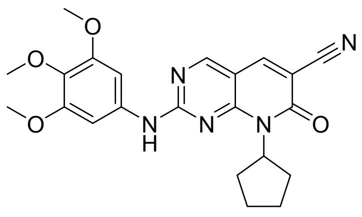

8-Cyclopentyl-2-(methylsulfinyl)-7-oxo-7,8-dihydropyrido[2,3-*d*]pyrimidine-6-carbonitrile (**62**) (35.0 mg, 0.12 mmol) and 3,4,5-trimethoxyaniline (**54**) (25.4 mg, 0.14 mmol) were reacted according to general procedure **B**. Purification via preparatory HPLC (0.1% NH_3 in MeCN/Water; 5-95%) yielded 8-cyclopentyl-7-oxo-2-((3,4,5-trimethoxyphenyl)amino)-7,8-dihydropyrido[2,3-*d*]pyrimidine-6-carbonitrile (**16**) (16.7 mg, 0.040 mmol, 34% yield) as a yellow lyophilised solid. MS (ESI+) *m/z* calcd for $C_{22}H_{24}N_5O_4^+$ $[M + H]^+$ 422.2, found 422.5. UPLC analysis (method D), 5.23 min, >98% purity. ^1H NMR (300 MHz, DMSO-*d6*) δ 10.45 (s, 1H), 8.85 (s, 1H), 8.58 (s, 1H), 7.12 (s, 2H), 5.93 (quint, *J* = 8.7 Hz, 1H), 3.79 (s, 6H), 3.66 (s, 3H), 2.16 (s, 2H), 2.00 – 1.75 (m, 4H), 1.51 (br s, 2H).

8-Cyclopentyl-2-((3,5-dimethoxyphenyl)amino)-7-oxo-7,8-dihydropyrido[2,3-*d*]pyrimidine-6-carbonitrile (17)

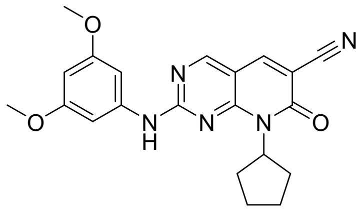

8-Cyclopentyl-2-(methylsulfinyl)-7-oxo-7,8-dihydropyrido[2,3-*d*]pyrimidine-6-carbonitrile (**62**) (35.0 mg, 0.12 mmol) and 3,5-dimethoxyaniline (21.3 mg, 0.14 mmol) were reacted according to general procedure **B**. Purification via preparatory HPLC (0.1% NH_3 in MeCN/Water; 30-76%) yielded 8-cyclopentyl-2-((3,5-dimethoxyphenyl)amino)-7-oxo-7,8-dihydropyrido[2,3-*d*]pyrimidine-6-carbonitrile (**17**) (18.0 mg, 0.046 mmol, 40% yield) as a yellow lyophilised solid. MS (ESI+) *m/z* calcd for $C_{21}H_{22}N_5O_3^+$ $[M + H]^+$ 392.2, found 392.4. UPLC analysis (method C), 5.66 min, 97% purity. ^1H NMR (300 MHz, DMSO-*d6*) δ 10.48 (s, 1H), 8.86 (s, 1H), 8.60 (s, 1H), 7.01 (d, *J* = 2.2 Hz, 2H), 6.29 (t, *J* = 2.2 Hz, 1H), 5.90 (quint, *J* = 8.6 Hz, 1H), 3.75 (s, 6H), 2.26 – 2.12 (m, 2H), 1.95 (br s, 2H), 1.89 – 1.77 (m, 2H), 1.63 – 1.49 (m, 2H).

8-Cyclopentyl-2-((3-methoxy-1-methyl-1*H*-pyrazol-4-yl)amino)-5-methylpyrido[2,3-*d*]pyrimidin-7(8*H*)-one (18)

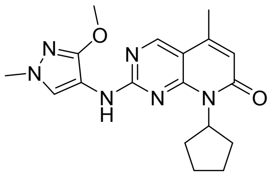

2-Chloro-8-cyclopentyl-5-methylpyrido[2,3-*d*]pyrimidin-7(8*H*)-one (**36**) (50.0 mg, 0.190 mmol) and 3-methoxy-1-methyl-1*H*-pyrazole-4-amine hydrochloride (**55**) (40.3 mg, 0.246 mmol) were reacted according to general procedure **A**. Purification via preparatory HPLC (0.1% NH_3 in MeCN/Water; 15-95%) yielded 8-cyclopentyl-2-((3-methoxy-1-methyl-1*H*-pyrazol-4-yl)amino)-5-methylpyrido[2,3-*d*]pyrimidin-7(8*H*)-one (**18**) (32.0 mg, 0.090 mmol, 48% yield) as a white lyophilised solid. MS (ESI+) *m/z* calcd for $C_{18}H_{23}N_6O_2^+$ $[M + H]^+$ 355.2, found 355.1. UPLC analysis (method D), 4.30 min, >98% purity. ^{1}H NMR (300 MHz, CDCl3) δ 8.62 (s, 1H), 7.67 (s, 1H), 6.72 (s, 1H), 6.25 (d, *J* = 1.2 Hz, 1H), 5.85 (quint, *J* = 8.7 Hz, 1H), 3.99 (s, 3H), 3.79 (s, 3H), 2.45 − 2.25 (m, 5H), 2.01 (s, 2H), 1.90 – 1.77 (m, 2H), 1.67 (s, 2H).

8-Cyclopentyl-6,6-dimethyl-2-((3,4,5-trimethoxyphenyl)amino)-6*H*-pyrimido[5,4-*b*][1,4]oxazin-7(8*H*)-one (19)

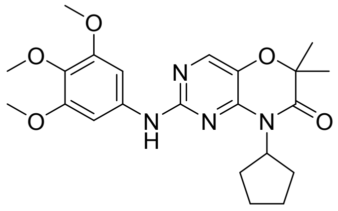

2-Chloro-8-cyclopentyl-6,6-dimethyl-6*H*-pyrimido[5,4-*b*][1,4]oxazin-7(8*H*)-one (**42**) (50.0 mg, 0.18 mmol) and 3,4,5-trimethoxyaniline (**54**) (42.3 mg, 0.23 mmol) in 1-BuOH (1.7 mL) were reacted according to general procedure **A**. Purification via preparatory HPLC (0.1% NH_3 in MeCN/Water; 30-95%) yielded 8-cyclopentyl-6,6-dimethyl-2-((3,4,5-trimethoxyphenyl)amino)-6*H*-pyrimido[5,4-*b*][1,4]oxazin-7(8*H*)-one (**19**) (19.3 mg, 0.045 mmol, 25% yield) as an off white lyophilised solid. MS (ESI+) *m/z* calcd for $C_{22}H_{29}N_4O_5^+$ $[M + H]^+$ 429.2, found 429.5. UPLC analysis (method D), 6.06 min, >98% purity. ^{1}H NMR (300 MHz, CDCl3) δ 7.99 (s, 1H), 6.86 (s, 2H), 6.83 (s, 1H), 5.40 (quint, *J* = 8.6 Hz, 1H), 3.90 (s, 6H), 3.85 (s, 3H), 2.13 (dt, *J* = 11.4, 7.6 Hz, 2H), 2.04 – 1.84 (m, 4H), 1.66 – 1.53 (m, 2H), 1.52 (s, 6H).

Table 3 Compounds

1-(2-Chloro-4-(isopropylamino)pyrimidin-5-yl)ethan-1-one (65)

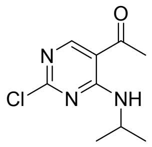

To a solution of 1-(2,4-dichloropyrimidin-5-yl)ethanone (**63**) (75.0 mg, 0.393 mmol) and $NaHCO_3$ (39.6 mg, 0.471 mmol) in THF (3.0 mL) was added a solution of isopropylamine (**64**) (24.4 mg, 0.412 mmol) in THF (1.0 mL) and the reaction mixture was stirred at rt for 2 hrs. After this time the reaction mixture was loaded into a plug of silica, eluted with DCM (50 mL) and concentrated in vacuo to give 1-(2-chloro-4-(isopropylamino)pyrimidin-5-yl)ethan-1-one (**65**) (77.5 mg, 0.363 mmol, 92% yield) as an orange oil which was used without further purification. MS (ESI+) *m/z* calcd for $C_9H_{13}ClN_3O^+$ $[M + H]^+$ 214.1, found 214.2. UPLC analysis (method A), 2.90 min, 49% purity.

2-Chloro-5-hydroxy-8-isopropyl-5-methyl-5,8-dihydropyrido[2,3-*d*]pyrimidin-7(6*H*)-one (66)

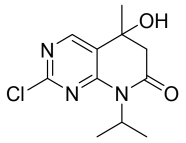

Lithium bis(trimethylsilyl)amide (1.0 M in toluene, 0.80 mL, 0.80 mmol) was added to THF (3.6 mL) and the mixture cooled to −78 °C. Then ethyl acetate (63.9 mg, 0.725 mmol) was added and the reaction mixture stirred at −78 °C for 20 min. Then 1-(2-chloro-4-(isopropylamino)pyrimidin-5-yl)ethan-1-one (**65**) (77.5 mg, 0.363 mmol) in THF (0.5 mL) was added and the reaction mixture stirred at −78 °C for a further 5 min, then removed from the cooling bath and allowed to warm to rt. After 5 hr the reaction was quenched with sat. aq. NH_4Cl, then extracted with EtOAc (2 × 25 mL), then the combined organics were washed with brine (50 mL), dried (hydrophobic frit) and concentrated in vacuo. Purification via silica gel chromatography (gradient elution 10 to 100% EtOAc in petroleum ether) yielded 2-chloro-5-hydroxy-8-isopropyl-5-methyl-5,8-dihydropyrido[2,3-*d*]pyrimidin-7(6*H*)-one (**66**) (17.0 mg, 0.066 mmol, 18% yield) as a yellow solid, with the expected dehydrated product not isolated. MS (ESI+) *m/z* calcd for $C_{11}H_{15}ClN_3O_2^+$ $[M + H]^+$ 256.1, found 256.2. UPLC analysis (method A), 2.68 min, 90% purity. ^1H NMR (300 MHz, $CDCl_3$) δ 8.52 (s, 1H), 5.30 (hept, *J* = 6.9 Hz, 1H), 2.94 (d, *J* = 15.8 Hz, 1H), 2.83 (d, *J* = 15.8 Hz, 1H), 2.39 (s, 1H), 1.62 (s, 3H), 1.52 (dd, *J* = 6.9, 3.1 Hz, 6H).

8-Isopropyl-5-methyl-2-((4-(6-methyl-3,6-diazabicyclo[3.1.1]heptan-3-yl)phenyl)amino)pyrido[2,3-*d*]pyrimidin-7(8*H*)-one (20)

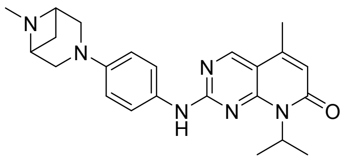

To a solution of 2-chloro-5-hydroxy-8-isopropyl-5-methyl-5,8-dihydropyrido[2,3-*d*]pyrimidin-7(6*H*)-one (**66**) (17.0 mg, 0.066 mmol) and 4-(6-methyl-3,6-diazabicyclo[3.1.1]heptan-3-yl)aniline (**51**) (16.2 mg, 0.080 mmol) were reacted according to general procedure **C**. Purification *via* preparatory HPLC (0.1% NH_3 in MeCN/Water; 15-72%) followed by purification *via* silica gel chromatography (gradient elution 0 to 20% MeOH (with 0.1 M NH_3) in DCM) yielded 8-isopropyl-5-methyl-2-((4-(6-methyl-3,6-diazabicyclo[3.1.1]heptan-3-yl)phenyl)amino)pyrido[2,3-*d*]pyrimidin-7(8*H*)-one (**20**) (3.6 mg, 0.009 mmol, 13% yield) as a yellow solid after lyophilisation. MS (ESI+) *m/z* calcd for $C_{23}H_{29}N_6O^+$ $[M + H]^+$ 405.2, found 405.1. UPLC analysis (method D), 3.62 min, >98% purity. ^1H NMR (300 MHz, $CDCl_3$) δ 8.62 (s, 1H), 7.56 (d, J = 8.9 Hz, 2H), 7.12 (s, 1H), 6.86 – 6.75 (m, 2H), 6.25 (d, J = 1.3 Hz, 1H), 5.76 (quint, J = 6.8 Hz, 1H), 4.31 (s, 2H), 3.74 (s, 3H), 3.25 (s, 1H), 2.48 (s, 2H), 2.38 (d, J = 1.2 Hz, 3H), 1.93 (d, J = 9.5 Hz, 2H), 1.60 (d, J = 7.0 Hz, 6H), 1.28 (s, 1H).

1-(2-Chloro-4-((2-methoxyethyl)amino)pyrimidin-5-yl)ethan-1-one (69)

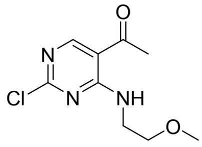

To a solution of 1-(2,4-dichloropyrimidin-5-yl)ethanone (**63**) (50.0 mg, 0.262 mmol) and $NaHCO_3$ (26.4 mg, 0.314 mmol) in THF (2.0 mL) was added a solution of 2-methoxyethylamine (**67**) (20.6 mg, 0.275 mmol) in THF (0.6 mL) and the reaction mixture was stirred at rt for 2 hrs. After this time the reaction mixture was loaded onto a plug of silica, eluted with DCM (50 mL) and concentrated in vacuo to give 1-(2-chloro-4-((2-methoxyethyl)amino)pyrimidin-5-yl)ethan-1-one (**69**) (59.0 mg, 0.257 mmol, 98% yield) as a yellow solid that was used without further purification. MS (ESI+) *m/z* calcd for $C_9H_{13}ClN_3O_2^+$ $[M + H]^+$ 230.1, found 230.1. UPLC analysis (method A), 2.45 min, 64% purity.

2-Chloro-8-(2-methoxyethyl)-5-methylpyrido[2,3-d]pyrimidin-7(8*H*)-one (71)

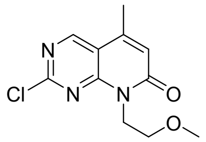

Lithium bis(trimethylsilyl)amide (1.0 M in toluene, 2.3 mL, 2.3 mmol) was added to THF (10.4 mL) and the mixture cooled to −78 °C. To this ethyl acetate (0.20 mL, 2.1 mmol) was added and the reaction mixture stirred at −78 °C for 20 min. To this 1-(2-chloro-4-((2-methoxyethyl)amino)pyrimidin-5-yl)ethan-1-one (**69**) (239 mg, 1.04 mmol) in THF (0.5 mL) was added and the reaction mixture stirred at −78 °C for a further 5 min, then removed from the cooling bath and allowed to warm to rt. After 2 hr the reaction was quenched by the addition of sat. aq. NH_4Cl (5 mL), then extracted with EtOAc (2 × 25 mL), then the combined organics were washed with brine (50 mL), dried (hydrophobic frit) and concentrated in vacuo. Purification via silica gel chromatography (gradient elution 10 to 100% EtOAc in petroleum ether) yielded 2-chloro-8-(2-methoxyethyl)-5-methylpyrido[2,3-d]pyrimidin-7(8*H*)-one (**71**) (56.0 mg, 0.221 mmol, 21% yield) as a yellow solid. MS (ESI+) *m/z* calcd for $C_{11}H_{13}ClN_3O_2^+$ $[M + H]^+$ 254.1, found 254.2. UPLC analysis (method A), 2.48 min, 90% purity. ^1H NMR (300 MHz, $CDCl_3$) δ 8.79 (s, 1H), 6.63 – 6.59 (m, 1H), 4.65 (t, *J* = 5.7 Hz, 2H), 3.76 (t, *J* = 5.7 Hz, 2H), 3.40 (s, 3H), 2.50 (d, *J* = 1.3 Hz, 3H).

8-(2-Methoxyethyl)-5-methyl-2-((4-(6-methyl-3,6-diazabicyclo[3.1.1]heptan-3-yl)phenyl)amino)pyrido[2,3-d]pyrimidin-7(8*H*)-one (21)

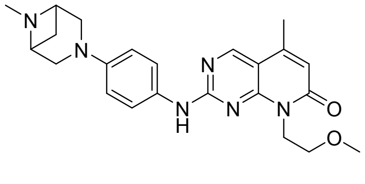

2-Chloro-8-(2-methoxyethyl)-5-methylpyrido[2,3-d]pyrimidin-7(8*H*)-one (**71**) (28.0 mg, 0.110 mmol) and 4-(6-methyl-3,6-diazabicyclo[3.1.1]heptan-3-yl)aniline (**51**) (26.9 mg, 0.132 mmol) were reacted according to general procedure **C**. Purification *via* preparatory HPLC (0.1% NH_3 in MeCN/Water; 15-62%) followed by purification *via* silica gel chromatography (gradient elution 0% to 20% MeOH (with 0.1% NH_3) in DCM) yielded 8-(2-methoxyethyl)-5-methyl-2-((4-(6-methyl-3,6-diazabicyclo[3.1.1]heptan-3-yl)phenyl)amino)pyrido[2,3-d]pyrimidin-7(8*H*)-one (**21**) (12.0 mg, 0.029 mmol, 26% yield) as a lyophilised yellow solid. MS (ESI+) *m/z* calcd for $C_{23}H_{29}N_6O_2^+$ $[M + H]^+$ 421.2, found 421.4. UPLC analysis (method D), 3.29 min, >95% purity. ^1H NMR (300 MHz, $CDCl_3$) δ 8.63 (s, 1H), 7.60 (d, *J* = 8.8 Hz, 2H), 7.20 (s, 1H), 6.80 (d, *J* = 9.1 Hz, 2H), 6.31 (d, *J* = 1.2 Hz, 1H), 4.61 (t, *J* = 6.4 Hz, 2H), 4.20 (s, 2H), 3.80 – 3.64 (m, 5H), 3.39 (s, 3H), 3.20 – 3.12 (m, 1H), 2.46 – 2.38 (m, 5H), 1.88 (d, *J* = 9.4 Hz, 2H), 1.33 – 1.26 (m, 1H).

1-(2-Chloro-4-(((2,2-dimethyl-1,3-dioxolan-4-yl)methyl)amino)pyrimidin-5-yl)ethan-1-one (70)

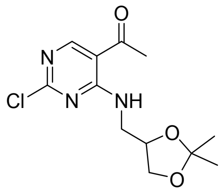

To a solution of 1-(2,4-dichloropyrimidin-5-yl)ethanone (**63**) (75.0 mg, 0.393 mmol) and $NaHCO_3$ (39.6 mg, 0.471 mmol) in THF (3.0 mL) was added a solution of (2,2-dimethyl-1,3-dioxolan-4-yl)methanamine (**68**) (54.1 mg, 0.412 mmol) in THF (1.0 mL) and the reaction mixture was stirred at rt for 2 hrs. After this time the reaction mixture was loaded into a plug of silica, eluted with DCM (50 mL) and concentrated in vacuo to give 1-(2-chloro-4-(((2,2-dimethyl-1,3-dioxolan-4-yl)methyl)amino)pyrimidin-5-yl)ethan-1-one (**70**) (90.0 mg, 0.315 mmol, 80% yield) as an orange oil which was used without further purification. MS (ESI+) *m/z* calcd for $C_{12}H_{17}ClN_3O_3^+$ $[M + H]^+$ 286.1, found 286.1. UPLC analysis (method A), 2.71 min, 54% purity.

2-Chloro-8-((2,2-dimethyl-1,3-dioxolan-4-yl)methyl)-5-methylpyrido[2,3-d]pyrimidin-7(8*H*)-one (72)

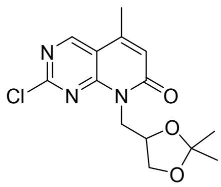

Lithium bis(trimethylsilyl)amide (1.0 M in toluene, 0.13 mL, 0.13 mmol) was added to THF (3.2 mL) and the mixture cooled to −78 °C. Then ethyl acetate (55.5 mg, 0.630 mmol) was added and the reaction mixture stirred at −78 °C for 20 min. Then 1-(2-chloro-4-(((2,2-dimethyl-1,3-dioxolan-4-yl)methyl)amino)pyrimidin-5-yl)ethan-1-one (**70**) (90.0 mg, 0.315 mmol) in THF (0.5 mL) was added and the reaction mixture stirred at −78 °C for a further 5 min, then removed from the cooling bath and allowed to warm to rt. After 2 hr the reaction was quenched by the addition of sat. aq. NH_4Cl, then extracted with EtOAc (2 × 25 mL), then the combined organics were washed with brine (50 mL), dried (hydrophobic frit) and concentrated in vacuo. Purification *via* silica gel chromatography (gradient elution 10 to 100% EtOAc in petroleum ether) yielded 2-chloro-8-((2,2-dimethyl-1,3-dioxolan-4-yl)methyl)-5-methylpyrido[2,3-d]pyrimidin-7(8*H*)-one (**72**) (26.0 mg, 0.084 mmol, 27% yield) as a pale yellow oil. An impurity corresponding to product plus H_2O was also present (29%) but carried forward without further purification. MS (ESI+) *m/z* calcd for $C_{14}H_{17}ClN_3O_3^+$ $[M + H]^+$ 310.1, found 310.1. UPLC analysis (method A), 2.71 min, 71% purity.

8-(2,3-Dihydroxypropyl)-5-methyl-2-((4-(6-methyl-3,6-diazabicyclo[3.1.1]heptan-3-yl)phenyl)amino)pyrido[2,3-*d*]pyrimidin-7(8*H*)-one (22)

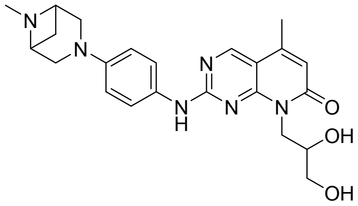

2-Chloro-8-((2,2-dimethyl-1,3-dioxolan-4-yl)methyl)-5-methylpyrido[2,3-*d*]pyrimidin-7(8*H*)-one (**72**) (26.0 mg, 0.084 mmol) and 4-(6-methyl-3,6-diazabicyclo[3.1.1]heptan-3-yl)aniline (**51**) (20.5 mg, 0.101 mmol) were reacted according to general procedure **C**. Purification *via* preparatory HPLC (0.1% NH_3 in MeCN/Water; 15-52%) yielded 8-(2,3-dihydroxypropyl)-5-methyl-2-((4-(6-methyl-3,6-diazabicyclo[3.1.1]heptan-3-yl)phenyl)amino)pyrido[2,3-d]pyrimidin-7(8*H*)-one (**22**) (6.1 mg, 0.014 mmol, 17% yield) as a yellow lyophilised solid. MS (ESI+) *m/z* calcd for $C_{23}H_{29}N_6O_3^+$ [M + H]$^+$ 437.2, found 437.4. UPLC analysis (method D), 2.77 min, >98% purity. ^{1}H NMR (300 MHz, $CDCl_3$) δ 8.69 (s, 1H), 7.45 (d, J = 8.9 Hz, 2H), 7.23 (s, 1H), 6.80 (d, J = 9.0 Hz, 2H), 6.34 (d, J = 1.2 Hz, 1H), 4.61 – 4.48 (m, 2H), 4.08 (s, 1H), 3.74 (d, J = 5.9 Hz, 2H), 3.59 (d, J = 11.0 Hz, 2H), 3.53 (d, J = 4.4 Hz, 2H), 3.36 (d, J = 10.9 Hz, 2H), 2.71 – 2.59 (m, 1H), 2.45 (d, J = 1.1 Hz, 3H), 2.18 (s, 3H), 1.68 (br s, 1H).

7-Methyl-*N*-(4-(4-methylpiperazin-1-yl)phenyl)-7*H*-pyrrolo[2,3-*d*]pyrimidin-2-amine (23)

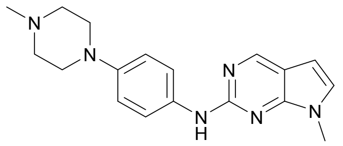

2-Chloro-7-methyl-7*H*-pyrrolo[2,3-*d*]pyrimidine (**79**) (50.0 mg, 0.300 mmol), 4-(4-methylpiperazin-1-yl)anilin (**37**) (86.6 mg, 0.450 mmol), tris(dibenzylideneacetone)dipalladium(0) chloroform adduct (15.4 mg, 0.010 mmol), *R*-BINAP (18.6 mg, 0.030 mmol) and sodium *tert*-butoxide (57.4 mg, 0.600 mmol) were sealed in a MW vial before 2-BuOH (1.5 mL) was added. The reaction mixture was degassed with N_2, then heated under microwave irradiation at 120 °C for 1h before the reaction mixture was diluted with EtOAc (25 mL) and washed with sat $NaHCO_3$ (25 mL). The aqueous layer was further extracted with EtOAc (25 mL) and combined organic extracts were washed with brine, dried (Na_2SO_4) and concentrated in vacuo. The crude material was taken up in DMSO and purified via preparative HPLC (elution gradient 40-80% of MeCN in H_2O with 0.1% NH_3) to yield 7-methyl-*N*-(4-(4-methylpiperazin-1-yl)phenyl)-7*H*-pyrrolo[2,3-*d*]pyrimidin-2-amine (**23**) (7.5 mg, 0.023 mmol, 8%

yield) as a white solid. MS (ESI+) *m/z* calcd for $C_{18}H_{23}N_6^+$ [M + H]$^+$ 323.2, found 323.2. UPLC analysis (method C), 4.69 min, >95% purity. ^{1}H NMR (300 MHz, DMSO-*d6*) δ 9.13 (s, 1H), 8.62 (s, 1H), 7.78 – 7.66 (m, 2H), 7.18 (d, *J* = 3.6 Hz, 1H), 6.95 – 6.83 (m, 2H), 6.39 (d, *J* = 3.5 Hz, 1H), 3.70 (s, 3H), 3.10 – 3.00 (m, 4H), 2.48 − 2.43 (m, 4H), 2.22 (s, 3H).

2-Chloro-7-((tetrahydro-2*H*-pyran-4-yl)methyl)-7*H*-pyrrolo[2,3-*d*]pyrimidine (80)

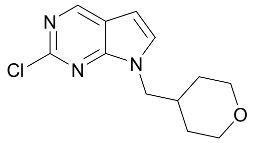

Sodium hydride (26.0 mg, 0.650 mmol) was sealed in a flask and DMF (1 mL) was added, followed by a solution of 2-chloro-7*H*-pyrrolo[2,3-*d*]pyrimidine (**44**) (50.0 mg, 0.330 mmol) in DMF (3.26 mL), and was stirred for 5 min until effervescences had stopped. Then 4-(bromomethyl)tetrahydro-2*H*-pyran (**73**) (87.5 mg, 0.490 mmol) was added and the reaction stirred at rt for 1 hr. After this time the reaction was quenched with water and extracted with EtOAc before washing with 10% wt aq. LiCl solution. The organic layer was dried (Na_2SO_4), filtered and concentrated *in vacuo*. Crude material was purified via silica gel chromatography (gradient elution 0 to 50% EtOAc in petroleum ether) to yield 2-chloro-7-((tetrahydro-2*H*-pyran-4-yl)methyl)-7*H*-pyrrolo[2,3-*d*]pyrimidine (**80**) (55 mg, 0.219 mmol, 67% yield). Material carried forward for next step without further purification. MS (ESI+) *m/z* calcd for $C_{12}H_{15}ClN_3O^+$ [M + H]$^+$ 252.2, found 252.2. UPLC analysis (method A), 2.54 min, 76% purity.

***N*-(4-(6-Methyl-3,6-diazabicyclo[3.1.1]heptan-3-yl)phenyl)-7-((tetrahydro-2*H*-pyran-4-yl)methyl)-7*H*-pyrrolo[2,3-*d*]pyrimidin-2-amine (24)**

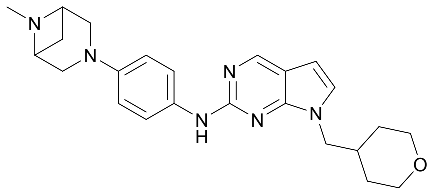

2-Chloro-7-((tetrahydro-2*H*-pyran-4-yl)methyl)-7*H*-pyrrolo[2,3-*d*]pyrimidine (**80**) (28.0 mg, 0.110 mmol) and 4-(3-ethyl-4-methyl-piperazin-1-yl)aniline (**51**) (36.6 mg, 0.170 mmol) were reacted via general procedure **D** to yield *N*-(4-(6-methyl-3,6-diazabicyclo[3.1.1]heptan-3-yl)phenyl)-7-((tetrahydro-2*H*-pyran-4-yl)methyl)-7*H*-pyrrolo[2,3-*d*]pyrimidin-2-amine (**24**) (22 mg, 0.053 mmol, 47% yield). MS (ESI+) *m/z* calcd for $C_{24}H_{31}N_6O^+$ [M + H]$^+$ 419.3, found 419.4. UPLC analysis (method D), 2.44 min, >98% purity. MS (ESI+) *m/z* calcd for $C_{24}H_{31}N_6O^+$ [M + H]$^+$ 419.2559, found 419.2540. ^{1}H NMR (300 MHz, DMSO-*d6*) δ 8.96 (s, 1H), 8.60 (s, 1H), 7.70 (d, *J* = 9.1 Hz, 2H), 7.17 (d, *J* = 3.6

Hz, 1H), 6.68 (d, *J* = 9.1 Hz, 2H), 6.38 (d, *J* = 3.5 Hz, 1H), 4.01 (d, *J* = 7.1 Hz, 2H), 3.84 (dd, *J* = 11.4, 2.4 Hz, 2H), 3.57 (d, *J* = 5.7 Hz, 2H), 3.46 – 3.41 (m, 2H), 3.29 – 3.15 (m, 4H), 2.42 (dd, *J* = 13.2, 6.1 Hz, 1H), 2.23 – 2.07 (m, 1H), 1.98 (s, 3H), 1.54 (d, *J* = 8.1 Hz, 1H), 1.43 (d, *J* = 10.5 Hz, 2H), 1.36 – 1.20 (m, 2H). ^{13}C NMR (126 MHz, DMSO-*d6*) δ 156.4, 151.9, 150.3, 143.0, 131.0, 126.6, 120.1, 111.3, 109.5, 99.2, 66.5, 58.6, 48.9, 43.7, 35.4, 31.3, 30.3, 29.6, 29.0.

***N*-(4-(4-Isopropylpiperazin-1-yl)phenyl)-7-((tetrahydro-2*H*-pyran-4-yl)methyl)-7*H*-pyrrolo[2,3-*d*]pyrimidin-2-amine (25)**

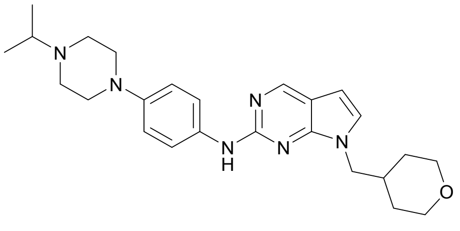

A suspension of 2-chloro-7-((tetrahydro-2*H*-pyran-4-yl)methyl)-7*H*-pyrrolo[2,3-*d*]pyrimidine (**80**) (80.0 mg, 0.320 mmol), 4-(4-isopropylpiperazin-1-yl)aniline (**35**) (105 mg, 0.480 mmol), tris(dibenzylideneacetone)dipalladium (0) chloroform adduct (16.5 mg, 0.020 mmol), 4,5-bis(diphenylphosphino)-9,9-dimethylxanthene (18.4mg, 0.030 mmol) and caesium carbonate (310 mg, 0.950 mmol) in 1,4-dioxane (1.60 mL) was degassed with N_2 for 10 mins before heating under MW irradiation 120 °C for 1 hr. After this time the reaction was stopped and the mixture placed onto an SCX-II cartridge, washed with DCM and MeOH before eluting with 0.5 M NH_3 in MeOH. The filtrate was concentrated *in vacuo* and the crude material taken up in DMSO before purification *via* preparative HPLC (gradient elution 50 to 90% MeCN in H_2O with 0.1% NH_3) to yield *N*-(4-(4-isopropylpiperazin-1-yl)phenyl)-7-((tetrahydro-2*H*-pyran-4-yl)methyl)-7*H*-pyrrolo[2,3-*d*]pyrimidin-2-amine (**25**) (50 mg, 0.115 mmol, 36% yield) as a lyophilised white solid. MS (ESI+) *m/z* calcd for $C_{25}H_{35}N_6O^+$ $[M + H]^+$ 435.3, found 435.3. UPLC analysis (method D), 2.88 min, >98% purity. ^{1}H NMR (300 MHz, DMSO-*d6*) δ 9.12 (s, 1H), 8.62 (s, 1H), 7.70 (d, *J* = 9.1 Hz, 2H), 7.20 (d, *J* = 3.6 Hz, 1H), 6.87 (d, *J* = 9.1 Hz, 2H), 6.39 (d, *J* = 3.5 Hz, 1H), 4.02 (d, *J* = 7.1 Hz, 2H), 3.83 (dd, *J* = 11.5, 2.5 Hz, 2H), 3.23 (td, *J* = 11.4, 1.9 Hz, 2H), 3.10 – 2.97 (m, 4H), 2.74 – 2.60 (m, 1H), 2.60 – 2.53 (m, 4H), 2.22 – 2.04 (m, 1H), 1.46 – 1.37 (m, 2H), 1.37 – 1.20 (m, 2H), 1.00 (d, *J* = 6.5 Hz, 6H).

(2,2-Dimethyltetrahydro-2*H*-pyran-4-yl)methyl 4-methylbenzenesulfonate (74)

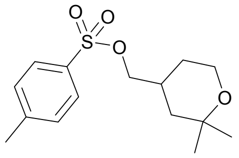

To a solution of (2,2-dimethyltetrahydro-2*H*-pyran-4-yl)methanol (50.0 mg, 0.350 mmol) in DCM (3.50 mL) was added 4-dimethylaminopyridine (50.8 mg, 0.420 mmol) and triethylamine (0.06 mL, 0.420 mmol) and the reaction mixture allowed to stir for 10 mins. After this time *p*-toluenesulfonyl chloride (72.7 mg, 0.380 mmol) was added and the reaction stirred at rt for 16 hrs. The reaction was concentrated in vacuo and the residue taken up in EtOAc before washing with NH_4Cl (aq), water and brine. The organic layer was dried (Na_2SO_4), filtered and solvent removed *in vacuo*. Crude material was purified *via* silica gel chromatography (gradient elution 2 to 50% EtOAc in petroleum ether) to yield (2,2-dimethyltetrahydro-2*H*-pyran-4-yl)methyl 4-methylbenzenesulfonate (**74**) (100 mg, 0.335 mmol, 97% yield) as a white waxy solid. MS (ESI+) *m/z* calcd for $C_{15}H_{23}O_4S^+$ $[M + H]^+$ 299.1, found 299.1. UPLC analysis (method B), 3.11 min, >98% purity. ^1H NMR (300 MHz, $CDCl_3$) δ 7.79 (d, J = 8.3 Hz, 2H), 7.35 (d, J = 8.0 Hz, 2H), 3.81 (d, J = 6.6 Hz, 2H), 3.78 – 3.66 (m, 1H), 3.60 (td, J = 12.2, 2.3 Hz, 1H), 2.46 (s, 3H), 2.17 – 2.01 (m, 2H), 1.58 – 1.47 (m, 2H), 1.22 (d, J = 2.7 Hz, 1H), 1.17 (d, J = 7.6 Hz, 6H).

2-Chloro-7-((2,2-dimethyltetrahydro-2*H*-pyran-4-yl)methyl)-7H-pyrrolo[2,3-*d*]pyrimidine (81)

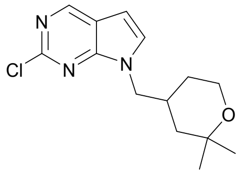

To a stirred suspension of sodium hydride (28.6 mg, 0.720 mmol) in THF (2.00 mL) was added 2-chloro-7*H*-pyrrolo[2,3-*d*]pyrimidine (**44**) (55 mg, 0.36 mmol) and the reaction mixture stirred for 5 min before the addition of (2,2-dimethyltetrahydropyran-4-yl)methyl 4-methylbenzenesulfonate (**74**) (89.0 mg, 0.300 mmol) in THF (1.0 mL). The reaction mixture was then heated at 80 °C for 48 hrs. After this time the reaction mixture was cooled and quenched with addition of H_2O. The aqueous layer was extracted with EtOAc (x3), the combined organic layer washed with brine, dried (Na_2SO_4), filtered and concentrated *in vacuo*. Crude material was purified via silica gel chromatography (gradient elution 0 to 20% MeOH in DCM) to yield 2-chloro-7-((2,2-dimethyltetrahydro-2*H*-pyran-4-yl)methyl)-7*H*-pyrrolo[2,3-*d*]pyrimidine (**81**) (35.0 mg, 0.125 mmol, 42% yield). MS (ESI+) *m/z* calcd for $C_{14}H_{19}ClN_3O^+$ $[M + H]^+$ 280.1, found 280.1. UPLC analysis (method A), 2.88 min, >98% purity

7-((2,2-Dimethyltetrahydro-2*H*-pyran-4-yl)methyl)-*N*-(4-(4-isopropylpiperazin-1-yl)phenyl)-7*H*-pyrrolo[2,3-*d*]pyrimidin-2-amine (26)

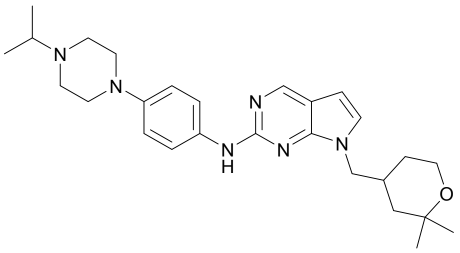

A suspension of 2-chloro-7-(tetrahydropyran-4-ylmethyl)pyrrolo[2,3-*d*]pyrimidine (**81**) (27.0 mg, 0.097 mmol), 4-(4-isopropylpiperazin-1-yl)aniline (**35**) (31.8 mg, 0.145 mmol), tris(dibenzylideneacetone)dipalladium (0) chloroform adduct (5.0 mg, 0.005 mmol), 4,5-bis(diphenylphosphino)-9,9-dimethylxanthene (5.6 mg, 0.010 mmol) and caesium carbonate (94.5 mg, 0.290 mmol) in 1,4-dioxane (0.95 mL) was degassed with N_2 for 10 mins before heating at 120 °C for 24 hrs. After this time the reaction was stopped and the mixture was placed onto an SCX-II cartridge, washed with DCM and MeOH, then eluted with 0.5 M NH_3 in MeOH and concentrated in vacuo. The crude material was taken up in DMSO before purification *via* preparative HPLC (gradient elution 50 to 90% MeCN in H_2O with 0.1% NH_3) to yield 7-((2,2-dimethyltetrahydro-2*H*-pyran-4-yl)methyl)-N-(4-(4-isopropylpiperazin-1-yl)phenyl)-7*H*-pyrrolo[2,3-*d*]pyrimidin-2-amine (**26**) (23 mg, 0.050 mmol, 52% yield) as a lyophilised white solid. MS (ESI+) *m/z* calcd for $C_{27}H_{39}N_6O^+$ $[M + H]^+$ 463.3, found 463.3. UPLC analysis (method D), 2.96 min, >98% purity. ^1H NMR (300 MHz, DMSO-d_6) δ 9.13 (s, 1H), 8.62 (s, 1H), 7.77 – 7.66 (m, 2H), 7.21 (d, *J* = 3.6 Hz, 1H), 6.94 – 6.81 (m, 2H), 6.39 (d, *J* = 3.5 Hz, 1H), 3.95 (sex, *J* = 7.1 Hz, 2H), 3.59 (dd, *J* = 12.0, 4.5 Hz, 1H), 3.48 (dd, *J* = 12.3, 9.9 Hz, 1H), 3.04 (t, *J* = 5.0 Hz, 4H), 2.67 (quint, *J* = 6.5 Hz, 1H), 2.57 (dd, *J* = 9.7, 4.7 Hz, 4H), 2.36 (s, 1H), 1.45 (d, *J* = 12.3 Hz, 1H), 1.34 (d, *J* = 12.6 Hz, 1H), 1.23 – 1.08 (m, 8H), 1.01 (d, *J* = 6.5 Hz, 6H).

2-Chloro-7-((tetrahydrofuran-2-yl)methyl)-7*H*-pyrrolo[2,3-*d*]pyrimidine (82)

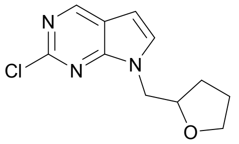

Sodium hydride (26.0 mg, 0.650 mmol) was sealed in a flask and DMF (1.0 mL) was added followed by a solution of 2-(bromomethyl)oxolane (**75**) (80.6 mg, 0.490 mmol) in DMF (3.3 mL) and stirred for 5 mins until effervescences has stopped. 2-Chloro-7*H*-pyrrolo[2,3-*d*]pyrimidine (**44**) (50.0 mg, 0.330 mmol) was then added and the reaction stirred at rt for 16 hrs. After this time the reaction was quenched

with water, extracted with EtOAc (x2) and the combined organic layer washed with 10% wt aq. LiCl solution. The organic layer was dried (Na_2SO_4), filtered and concentrated *in vacuo*. Crude material purified via silica gel chromatography (gradient elution 1 to 20% MeOH in DCM) yielding 2-chloro-7-((tetrahydrofuran-2-yl)methyl)-7*H*-pyrrolo[2,3-*d*]pyrimidine (**82**) (76.2 mg, 0.321 mmol, 98% yield). Material carried forward for next step without further purification. MS (ESI+) *m/z* calcd for $C_{11}H_{13}ClN_3O^+$ $[M + H]^+$ 238.1, found 238.2. UPLC analysis (method A), 2.58 min, 57% purity.

N-(4-(4-Isopropylpiperazin-1-yl)phenyl)-7-((tetrahydrofuran-2-yl)methyl)-7*H*-pyrrolo[2,3-*d*]pyrimidin-2-amine (27)

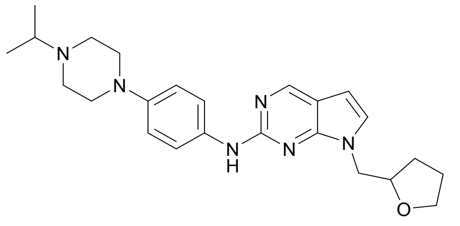

2-Chloro-7-((tetrahydrofuran-2-yl)methyl)-7*H*-pyrrolo[2,3-*d*]pyrimidine (**82**) (76.2 mg, 0.320 mmol) and 4-(4-isopropylpiperazin-1-yl)aniline (**35**) (140.6 mg, 0.640 mmol) were reacted according to general procedure **D** to yield (*N*-(4-(4-isopropylpiperazin-1-yl)phenyl)-7-((tetrahydrofuran-2-yl)methyl)-7*H*-pyrrolo[2,3-*d*]pyrimidin-2-amine (**27**) (78 mg,0.185 mmol, 58% yield). MS (ESI+) *m/z* calcd for $C_{24}H_{33}N_6O^+$ $[M + H]^+$ 421.3, found 421.5. UPLC analysis (method D), 2.80 min, >98% purity. ^1H NMR (300 MHz, DMSO-d_6) δ 9.11 (s, 1H), 8.62 (s, 1H), 7.69 (d, *J* = 8.9 Hz, 2H), 7.19 (d, *J* = 3.6 Hz, 1H), 6.87 (d, *J* = 9.0 Hz, 2H), 6.39 (d, *J* = 3.6 Hz, 1H), 4.29 – 4.19 (m, 1H), 4.16 (d, *J* = 5.0 Hz, 2H), 3.78 (q, *J* = 7.3 Hz, 1H), 3.63 (q, *J* = 7.3 Hz, 1H), 3.03 (t, *J* = 4.8 Hz, 4H), 2.67 (quint, *J* = 6.5 Hz, 1H), 2.57 (t, *J* = 4.8 Hz, 4H), 1.98 – 1.84 (m, 1H), 1.83 – 1.71 (m, 2H), 1.67 – 1.55 (m, 1H), 1.01 (d, *J* = 6.4 Hz, 6H).

N-(4-(6-Methyl-3,6-diazabicyclo[3.1.1]heptan-3-yl)phenyl)-7-((tetrahydrofuran-2-yl)methyl)-7*H*-pyrrolo[2,3-*d*]pyrimidin-2-amine (28)

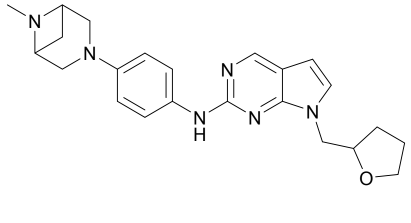

2-Chloro-7-((tetrahydrofuran-2-yl)methyl)-7*H*-pyrrolo[2,3-*d*]pyrimidine (**82**) (58.0 mg, 0.240 mmol) and 4-(3-ethyl-4-methyl-piperazin-1-yl)aniline (**51**) (80.3 mg, 0.370 mmol) were reacted according to

general procedure **D** to yield *N*-(4-(6-methyl-3,6-diazabicyclo[3.1.1]heptan-3-yl)phenyl)-7-((tetrahydrofuran-2-yl)methyl)-7*H*-pyrrolo[2,3-*d*]pyrimidin-2-amine (**28**) (30 mg, 0.074 mmol, 30% yield) as a lyophilised white solid. MS (ESI+) *m/z* calcd for $C_{23}H_{29}N_6O^+$ $[M + H]^+$ 405.2, found 405.4. UPLC analysis (method D), 2.43 min, >98% purity. ^{1}H NMR (300 MHz, DMSO-*d*6) δ 8.96 (s, 1H), 8.59 (s, 1H), 7.73 – 7.63 (m, 2H), 7.16 (d, *J* = 3.6 Hz, 1H), 6.71 – 6.63 (m, 2H), 6.36 (d, *J* = 3.6 Hz, 1H), 4.28 – 4.19 (m, 1H), 4.14 (dd, *J* = 5.4, 1.8 Hz, 2H), 3.83 – 3.73 (m, 1H), 3.68 – 3.60 (m, 1H), 3.56 (d, *J* = 5.8 Hz, 2H), 3.40 (d, *J* = 11.0 Hz, 2H), 3.24 (d, *J* = 11.0 Hz, 2H), 2.40 (q, *J* = 6.6 Hz, 1H), 1.97 (s, 3H), 1.95 – 1.85 (m, 1H), 1.83 – 1.72 (m, 2H), 1.67 – 1.57 (m, 1H), 1.53 (d, *J* = 8.0 Hz, 1H).

2-Chloro-7-((tetrahydrofuran-3-yl)methyl)-7*H*-pyrrolo[2,3-*d*]pyrimidine (83)

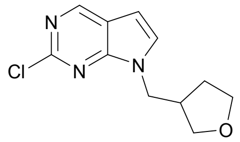

Sodium hydride (26.0 mg, 0.650 mmol) was sealed in a flask and DMF (1.0 mL) was added followed by a solution of 2-chloro-7*H*-pyrrolo[2,3-*d*]pyrimidine (**44**) (50.0 mg, 0.330 mmol) in DMF (3.3 mL). The reaction mixture was stirred for 5 min until effervescences has stopped. Tetrahydro-3-(iodomethyl)furan (**76**) (0.05 mL, 0.390 mmol) was then added and the reaction mixture stirred for 1 hr. After this time the reaction mixture was diluted with EtOAc and quenched with the addition of water. The organic layer was separated and washed with 10% wt aq. LiCl solution, dried (Na_2SO_4), filtered and concentrated in vacuo. The crude material was purified via silica gel chromatography (gradient elution 1 to 50% EtOAc in petroleum ether) to yield 2-chloro-7-((tetrahydrofuran-3-yl)methyl)-7*H*-pyrrolo[2,3-*d*]pyrimidine (**83**) (55.0 mg, 0.231 mmol, 71% yield) and carried forward without further purification. MS (ESI+) *m/z* calcd for $C_{11}H_{13}ClN_3O^+$ $[M + H]^+$ 238.1, found 238.2 UPLC analysis (method A), 2.44 min, 81% purity.

***N*-(4-(6-Methyl-3,6-diazabicyclo[3.1.1]heptan-3-yl)phenyl)-7-((tetrahydrofuran-3-yl)methyl)-7*H*-pyrrolo[2,3-*d*]pyrimidin-2-amine (29)**

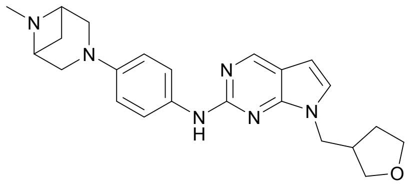

2-Chloro-7-((tetrahydrofuran-3-yl)methyl)-7*H*-pyrrolo[2,3-*d*]pyrimidine (**83**) (40.0 mg, 0.170 mmol) and 4-(3-ethyl-4-methyl-piperazin-1-yl)aniline (**51**) (55.4 mg, 0.250 mmol) were reacted according to

general procedure **D** to yield *N*-(4-(6-methyl-3,6-diazabicyclo[3.1.1]heptan-3-yl)phenyl)-7-((tetrahydrofuran-3-yl)methyl)-7*H*-pyrrolo[2,3-*d*]pyrimidin-2-amine (**29**) (14 mg, 0.035 mmol, 21% yield) as a lyophilised brown solid. MS (ESI+) *m/z* calcd for $C_{23}H_{29}N_6O^+$ $[M + H]^+$ 405.2, found 405.3. UPLC analysis (method D), 2.43 min, >98% purity. ^1H NMR (300 MHz, DMSO-*d6*) δ 8.96 (s, 1H), 8.59 (s, 1H), 7.67 (d, *J* = 9.0 Hz, 2H), 7.23 (d, *J* = 3.6 Hz, 1H), 6.67 (d, *J* = 9.1 Hz, 2H), 6.38 (d, *J* = 3.6 Hz, 1H), 4.09 (d, *J* = 7.5 Hz, 2H), 3.86 – 3.74 (m, 1H), 3.70 – 3.61 (m, 2H), 3.56 (d, *J* = 5.7 Hz, 2H), 3.55 – 3.48 (m, 1H), 3.45 – 3.20 (m, 4H), 2.90 – 2.76 (m, 1H), 2.41 (dd, *J* = 13.4, 6.2 Hz, 1H), 1.98 (s, 3H), 1.95 – 1.81 (m, 1H), 1.71 – 1.58 (m, 1H), 1.53 (d, *J* = 8.1 Hz, 1H).

2-Chloro-7-(tetrahydrofuran-3-yl)-7*H*-pyrrolo[2,3-*d*]pyrimidine (84)

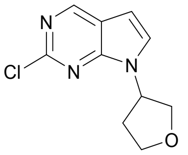

Sodium hydride (26.1 mg, 0.650 mmol) was sealed in a flask and DMF (1.0 mL) was added followed by a solution of 2-chloro-7*H*-pyrrolo[2,3-*d*]pyrimidine (**44**) (50.0 mg, 0.330 mmol) in DMF (3.3 mL). The reaction mixture was stirred for 5 mins until effervescences had stopped. Then 3-bromotetrahydrofuran (**77**) (0.05 mL, 0.49 mmol) was added and the reaction stirred for 16 hrs. After this time the reaction was quenched with water, extracted with EtOAc (x2) before washing with 10% wt aq. LiCl solution. The organic layer was dried (Na_2SO_4), filtered and solvent removed *in vacuo*. Crude material was purified via silica gel chromatography (gradient elution 0 to 50% EtOAc in petroleum ether) yielding 2-chloro-7-(tetrahydrofuran-3-yl)-7*H*-pyrrolo[2,3-*d*]pyrimidine (**84**) (55.0 mg, 0.246 mmol, 76% yield). Material carried forward for next step without further purification. MS (ESI+) *m/z* calcd for $C_{10}H_{11}ClN_3O^+$ $[M + H]^+$ 224.1, found 224.2. UPLC analysis (method A), 2.39 min, 70% purity.

***N*-(4-(6-Methyl-3,6-diazabicyclo[3.1.1]heptan-3-yl)phenyl)-7-(tetrahydrofuran-3-yl)-7*H*-pyrrolo[2,3-*d*]pyrimidin-2-amine (30)**

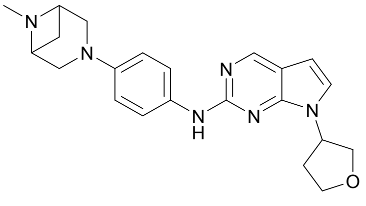

2-Chloro-7-(tetrahydrofuran-3-yl)-7*H*-pyrrolo[2,3-*d*]pyrimidine (**84**) (35.0 mg, 0.160 mmol) and 4-(3-ethyl-4-methyl-piperazin-1-yl)aniline (**51**) (51.5 mg, 0.230 mmol) were reacted according to general procedure **D** to yield *N*-(4-(6-methyl-3,6-diazabicyclo[3.1.1]heptan-3-yl)phenyl)-7-(tetrahydrofuran-3-yl)-7*H*-pyrrolo[2,3-*d*]pyrimidin-2-amine (**30**) (8.0 mg, 0.020 mmol, 13% yield) as a lyophilised brown solid. MS (ESI+) *m/z* calcd for $C_{22}H_{27}N_6O^+$ $[M + H]^+$ 391.2, found 391.3. UPLC analysis (method D), 2.14 min, >95% purity. ^{1}H NMR (300 MHz, DMSO-*d6*) δ 8.98 (s, 1H), 8.61 (s, 1H), 7.67 (d, *J* = 9.0 Hz, 2H), 7.19 (d, *J* = 3.7 Hz, 1H), 6.69 (d, *J* = 9.1 Hz, 2H), 6.42 (d, *J* = 3.7 Hz, 1H), 5.29 (ddt, *J* = 8.3, 6.1, 4.1 Hz, 1H), 4.09 (td, *J* = 8.2, 6.5 Hz, 1H), 4.01 (dd, *J* = 9.3, 6.1 Hz, 1H), 3.92 – 3.80 (m, 2H), 3.57 (d, *J* = 5.7 Hz, 2H), 3.46 – 3.43 (m, 1H), 3.25 (d, *J* = 10.8 Hz, 2H), 2.55 (s, 2H), 2.48 – 2.37 (m, 1H), 2.24 – 2.14 (m, 1H), 1.98 (s, 3H), 1.54 (d, *J* = 8.1 Hz, 1H).

2-Chloro-7-(oxetan-3-yl)pyrrolo[2,3-*d*]pyrimidine (85)

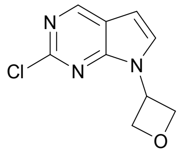

Sodium hydride (26.1 mg, 0.650 mmol) was sealed in a flask and DMF (1.0 mL) was added, followed by a solution of 2-chloro-7*H*-pyrrolo[2,3-*d*]pyrimidine (**44**) (50.0 mg, 0.330 mmol) in DMF (3.3 mL) and stirred for 5 mins until effervescences has stopped. Then 3-bromooxetane (**78**) (67.0 mg, 0.490 mmol) was added and the reaction stirred 16 hrs. After this time the reaction mixture was quenched with water, extracted with EtOAc before washing with 10% wt aq. LiCl solution. The organic layer was dried (Na_2SO_4), filtered and solvent removed *in vacuo*. Crude material was purified via silica gel chromatography (gradient elution 0 to 50% EA in Petrol) yielding 2-chloro-7-(oxetan-3-yl)pyrrolo[2,3-*d*]pyrimidine (**85**) (55 mg, 0.262 mmol, 81% yield). Material carried forward for next step without further purification. MS (ESI+) *m/z* calcd for $C_9H_9ClN_3O^+$ $[M + H]^+$ 210.1, found 210.1. UPLC analysis (method A), 2.20 min, 60% purity.

***N*-(4-(6-Methyl-3,6-diazabicyclo[3.1.1]heptan-3-yl)phenyl)-7-(oxetan-3-yl)-7*H*-pyrrolo[2,3-*d*]pyrimidin-2-amine (31)**

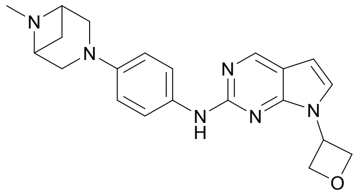

2-Chloro-7-(oxetan-3-yl)pyrrolo[2,3-*d*]pyrimidine (**85**) (14.0 mg, 0.070 mmol) and 4-(3-ethyl-4-methyl-piperazin-1-yl)aniline (**51**) (22.0 mg, 0.100 mmol) were reacted according to general procedure **D** to *N*-(4-(6-methyl-3,6-diazabicyclo[3.1.1]heptan-3-yl)phenyl)-7-(oxetan-3-yl)-7*H*-pyrrolo[2,3-*d*]pyrimidin-2-amine (**31**) (5.7 mg, 0.015 mmol, 23% yield). MS (ESI+) *m/z* calcd for $C_{21}H_{25}N_6O^+$ [M + H]$^+$ 376.2, found 377.3. UPLC analysis (method D), 2.25 min, >98% purity. ^{1}H NMR (300 MHz, DMSO-*d6*) δ 9.03 (s, 1H), 8.63 (s, 1H), 7.69 (d, *J* = 9.1 Hz, 2H), 7.53 (d, *J* = 3.7 Hz, 1H), 6.70 (d, *J* = 9.1 Hz, 2H), 6.49 (d, *J* = 3.7 Hz, 1H), 5.78 (quint, *J* = 7.4 Hz, 1H), 5.02 (dt, *J* = 14.7, 6.8 Hz, 4H), 3.61 – 3.49 (m, 2H), 3.28 – 3.18 (m, 2H), 2.46 – 2.36 (m, 2H), 2.05 – 2.00 (m, 1H), 1.99 (s, 3H), 1.55 (d, *J* = 8.0 Hz, 1H).

NMR spectra and LC-MS traces of compounds from Table 4

Compound 4

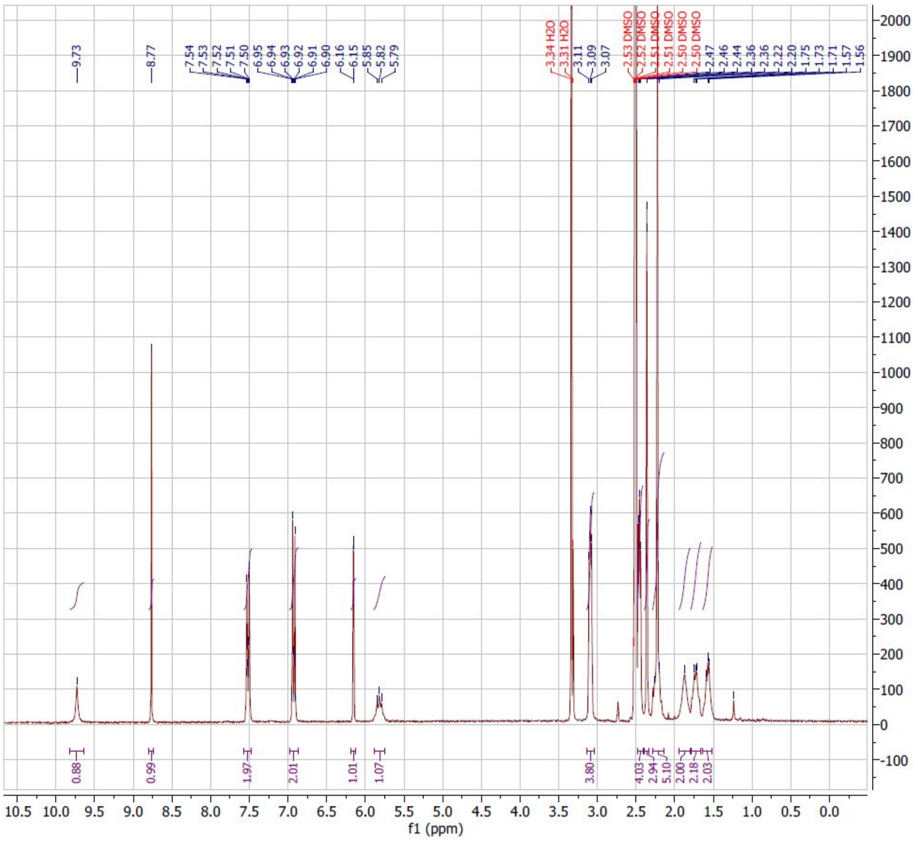

	Parameter	Value
1	Title	tpcr6-075-4.10.fid
2	Origin	Bruker BioSpin GmbH
3	Solvent	DMSO
4	Temperature	295.7
5	Pulse Sequence	zg30
6	Experiment	1D
7	Probe	5 mm PABBO BB-1H/ D Z-GRD Z104275/ 0242
8	Number of Scans	64
9	Receiver Gain	191.9
10	Relaxation Delay	1.0000
11	Pulse Width	14.0000
12	Presaturation Frequency	
13	Modification Date	2019-08-05T13:23:04
14	Spectrometer Frequency	300.13
15	Spectral Width	6009.6
16	Lowest Frequency	-1151.4
17	Nucleus	1H
18	Acquired Size	32768
19	Spectral Size	65536
20	Digital Resolution	

^{1}H NMR (300 MHz, DMSO) δ 9.73 (s, 1H), 8.77 (s, 1H), 7.57 – 7.47 (m, 2H), 6.98 – 6.87 (m, 2H), 6.15 (d, *J* = 1.3 Hz, 1H), 5.89 – 5.75 (m, 1H), 3.09 (t, *J* = 5.0 Hz, 4H), 2.46 (t, *J* = 5.0 Hz, 4H), 2.36 (d, *J* = 1.2 Hz, 3H), 2.22 (s, 5H), 1.94 – 1.80 (m, 2H), 1.79 – 1.66 (m, 2H), 1.57 (t, *J* = 5.8 Hz, 2H).

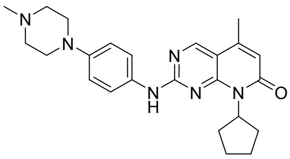

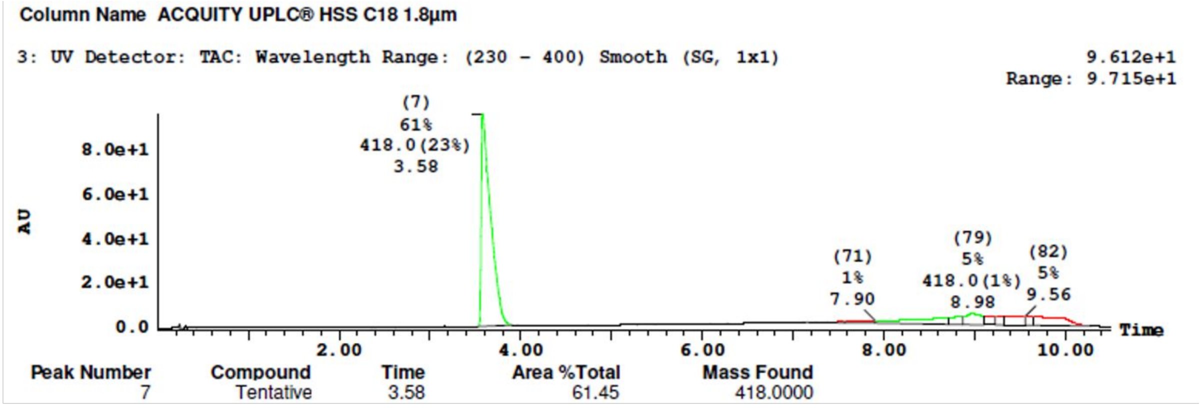

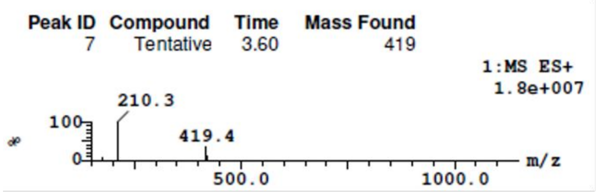

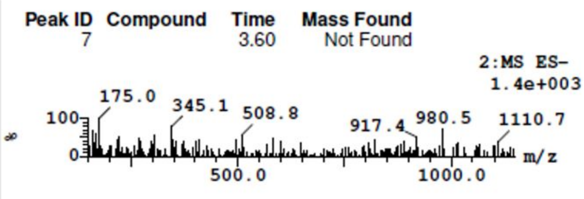

Compound 5

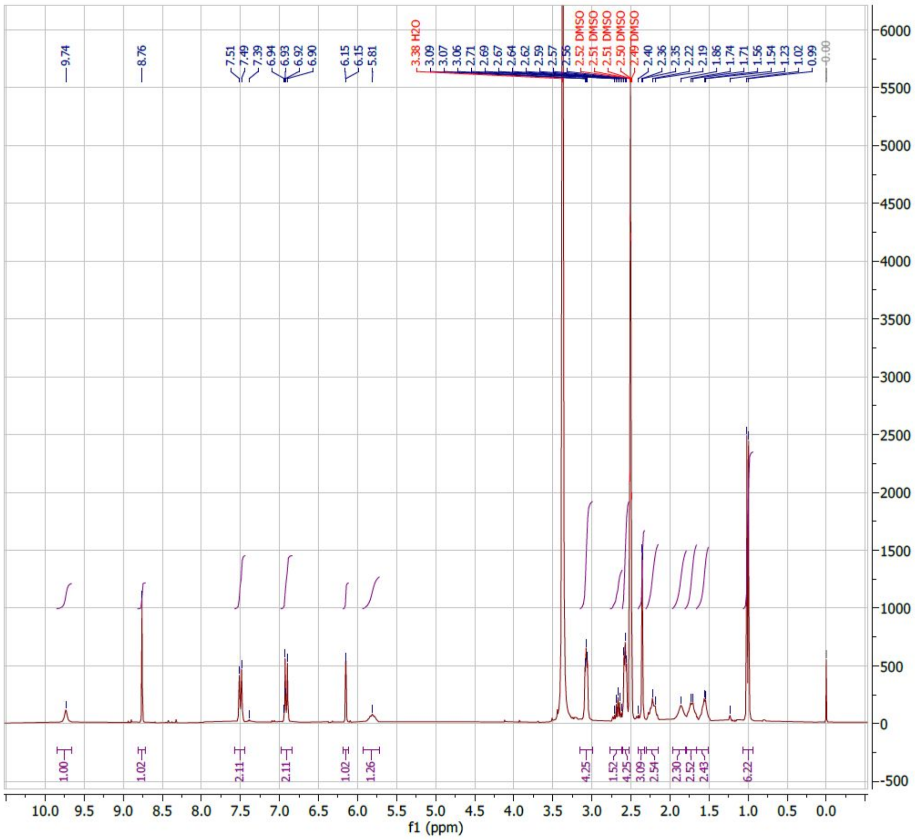

Parameter		Value
1	Title	GGA4-073-2-6.10.fid
2	Origin	Bruker BioSpin GmbH
3	Solvent	DMSO
4	Temperature	291.2
5	Pulse Sequence	zg30
6	Experiment	1D
7	Probe	5 mm PABBO BB-1H/ D Z-GRD Z104275/ 0242
8	Number of Scans	64
9	Receiver Gain	191.9
10	Relaxation Delay	1.0000
11	Pulse Width	14.0000
12	Presaturation Frequency	
13	Modification Date	2020-03-12T10:06:16
14	Spectrometer Frequency	300.13
15	Spectral Width	6009.6
16	Lowest Frequency	-1152.0
17	Nucleus	1H
18	Acquired Size	32768
19	Spectral Size	65536
20	Digital Resolution	

^{1}H NMR (300 MHz, DMSO) δ 9.74 (s, 1H), 8.76 (s, 1H), 7.50 (d, *J* = 8.7 Hz, 2H), 6.91 (d, *J* = 9.0 Hz, 2H), 6.15 (d, *J* = 1.3 Hz, 1H), 5.81 (s, 1H), 3.07 (t, *J* = 5.0 Hz, 4H), 2.67 (p, *J* = 6.5 Hz, 2H), 2.57 (t, *J* = 5.0 Hz, 4H), 2.35 (d, *J* = 1.1 Hz, 3H), 2.31 – 2.15 (m, 3H), 1.86 (s, 2H), 1.81 – 1.66 (m, 3H), 1.66 – 1.51 (m, 2H), 1.01 (d, *J* = 6.5 Hz, 6H).

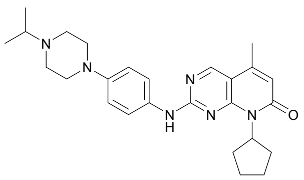

Column Name ACQUITY UPLC® HSS C18 1.8μm

3: UV Detector: TAC: Wavelength Range: (230 - 400) Smooth (SG, 1x1) 1.344e+1
Range: 1.679e+1

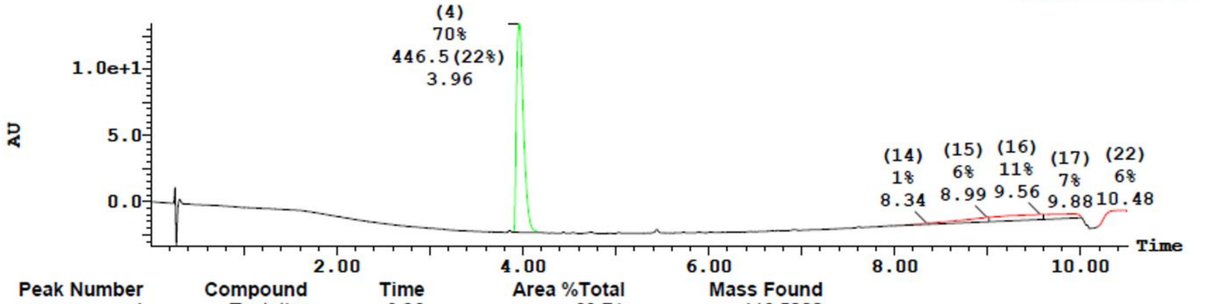

Peak Number	Compound	Time	Area %Total	Mass Found
4	Tentative	3.96	69.71	446.5000

Peak ID	Compound	Time	Mass Found
4	Tentative	3.96	447

1:MS ES+
2.3e+007

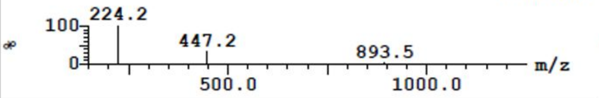

Peak ID	Compound	Time	Mass Found
4		3.96	Not Found

2:MS ES-
3.5e+003

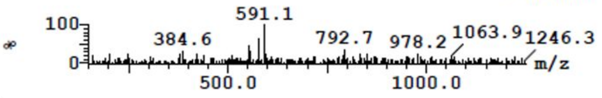

Compound 7

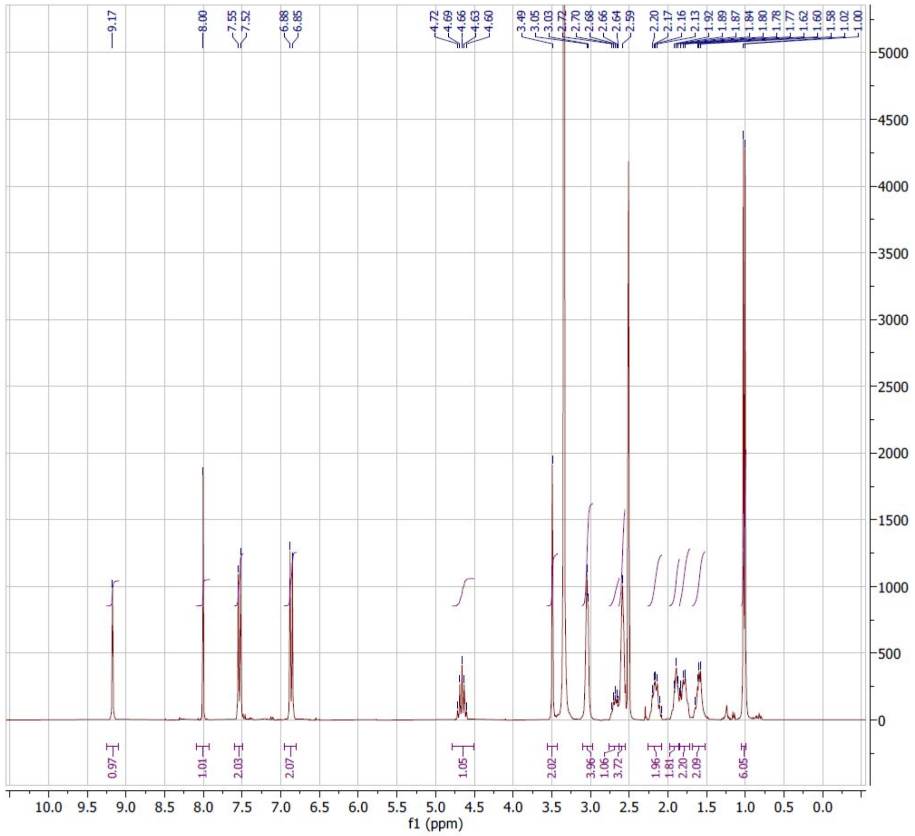

Parameter		Value
1	Title	GGA5-019-2
2	Origin	Bruker BioSpin GmbH
3	Solvent	DMSO
4	Temperature	296.2
5	Pulse Sequence	zg30
6	Experiment	
7	Probe	5 mm PABBO BB-1H/ D Z-GRD Z104275/ 0242
8	Number of Scans	64
9	Receiver Gain	191.9
10	Relaxation Delay	1.0000
11	Pulse Width	14.0000
12	Presaturation Frequency	
13	Modification Date	2020-10-08T15:02:08
14	Spectrometer Frequency	300.13
15	Spectral Width	6009.6
16	Lowest Frequency	-1151.4
17	Nucleus	1H
18	Acquired Size	32768
19	Spectral Size	65536
20	Digital Resolution	

^{1}H NMR (300 MHz, DMSO) δ 9.17 (s, 1H), 8.00 (s, 1H), 7.54 (d, *J* = 9.0 Hz, 2H), 6.87 (d, *J* = 9.1 Hz, 2H), 4.66 (p, *J* = 8.7 Hz, 1H), 3.49 (s, *J* = 20.1 Hz, 2H), 3.11 – 2.97 (m, 4H), 2.76 – 2.63 (m, 1H), 2.63 – 2.55 (m, 4H), 2.26 – 2.08 (m, 2H), 1.98 – 1.85 (m, 2H), 1.85 – 1.72 (m, 2H), 1.68 – 1.53 (m, 2H), 1.01 (d, *J* = 6.5 Hz, 6H).

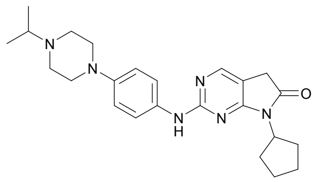

Unstable to LCMS conditions.

Compound 8

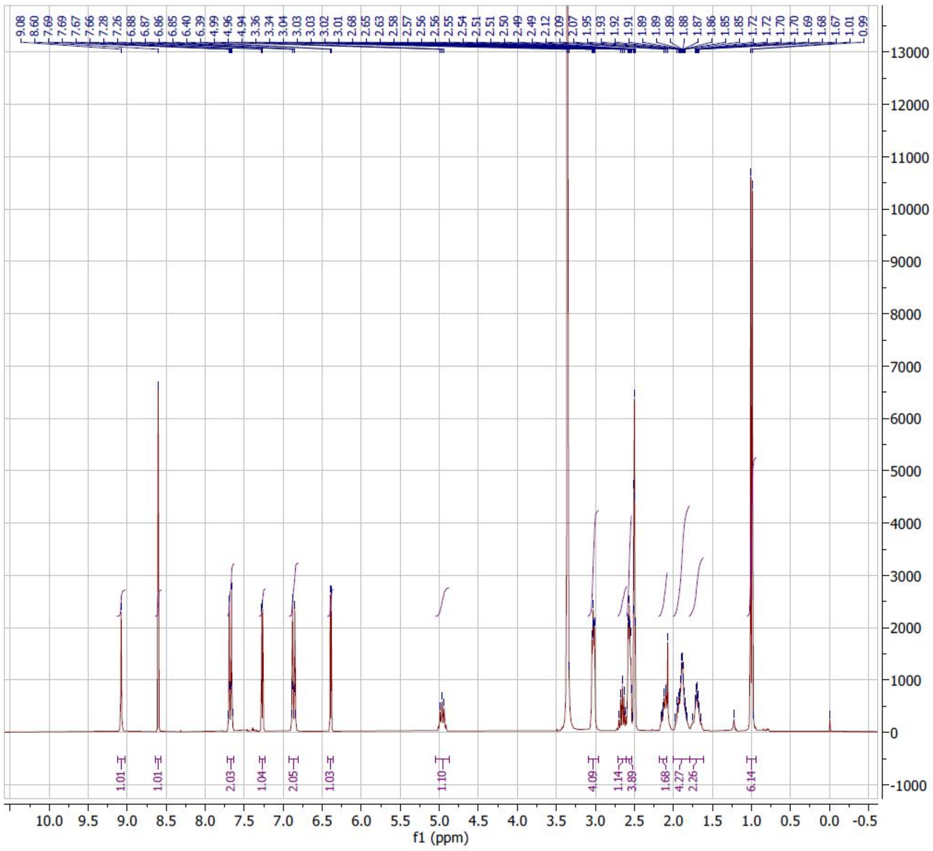

	Parameter	Value
1	Title	GGA4-063-2
2	Origin	Bruker BioSpin GmbH
3	Solvent	DMSO
4	Temperature	295.0
5	Pulse Sequence	zg30
6	Experiment	
7	Probe	5 mm PABBO BB-1H/ D Z-GRD Z104275/ 0242
8	Number of Scans	64
9	Receiver Gain	191.9
10	Relaxation Delay	1.0000
11	Pulse Width	14.0000
12	Presaturation Frequency	
13	Modification Date	2020-02-18T18:23:24
14	Spectrometer Frequency	300.13
15	Spectral Width	6009.6
16	Lowest Frequency	-1153.8
17	Nucleus	1H
18	Acquired Size	32768
19	Spectral Size	65536
20	Digital Resolution	

^{1}H NMR (300 MHz, DMSO) δ 9.08 (s, 1H), 8.60 (s, 1H), 7.71 – 7.63 (m, 2H), 7.27 (d, J = 3.7 Hz, 1H), 6.92 – 6.81 (m, 2H), 6.39 (d, J = 3.6 Hz, 1H), 5.04 – 4.88 (m, 1H), 3.09 – 2.96 (m, 4H), 2.65 (p, J = 6.5 Hz, 1H), 2.60 – 2.54 (m, 4H), 2.18 – 2.08 (m, 2H), 2.00 – 1.80 (m, 4H), 1.80 – 1.62 (m, 2H), 1.00 (d, J = 6.5 Hz, 6H).

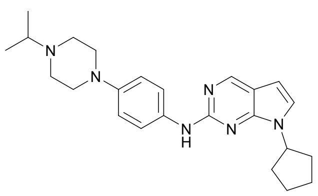

Column Name ACQUITY UPLC® HSS C18 1.8μm

3: UV Detector: TAC: Wavelength Range: (230 - 400) Smooth (SG, 1x1)

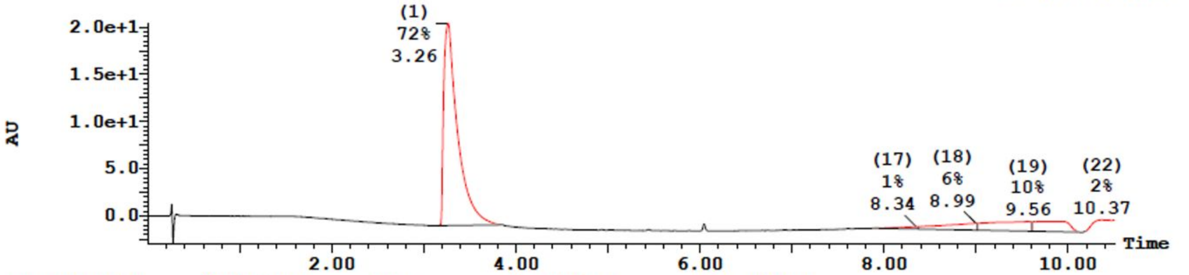

Peak Number	Compound	Time	Area %Total	Mass Found
1		3.26	71.98	Not Found

Peak ID	Compound	Time	Mass Found
1		3.26	Not Found

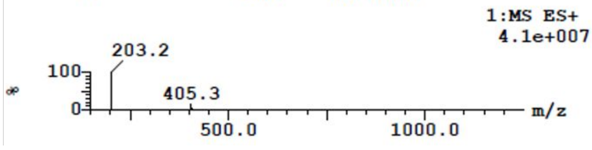

Peak ID	Compound	Time	Mass Found
1		3.26	Not Found

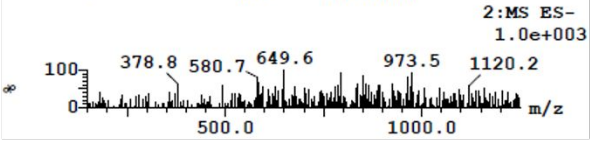

Compound 9

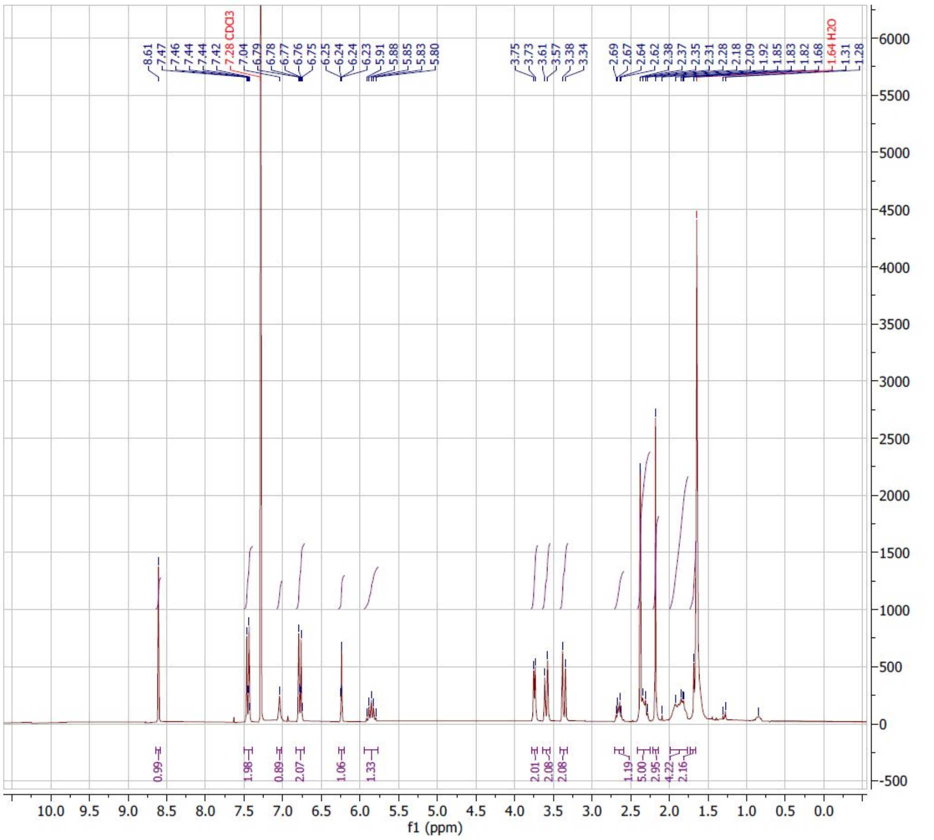

Parameter	Value
1 Title	tpcr7-048-3.10.fid
2 Origin	Bruker BioSpin GmbH
3 Solvent	CDCl3
4 Temperature	294.4
5 Pulse Sequence	zg30
6 Experiment	1D
7 Probe	5 mm PABBO BB-1H/ D Z-GRD Z104275/ 0242
8 Number of Scans	128
9 Receiver Gain	191.9
10 Relaxation Delay	1.0000
11 Pulse Width	14.0000
12 Presaturation Frequency	
13 Modification Date	2019-11-27T12:04:26
14 Spectrometer Frequency	300.13
15 Spectral Width	6009.6
16 Lowest Frequency	-1151.4
17 Nucleus	1H
18 Acquired Size	32768
19 Spectral Size	65536
20 Digital Resolution	

^{1}H NMR (300 MHz, $CDCl_3$) δ 8.61 (s, 1H), 7.50 – 7.39 (m, 2H), 7.04 (s, 1H), 6.82 – 6.72 (m, 2H), 6.24 (d, *J* = 1.2 Hz, 1H), 5.85 (p, *J* = 8.8 Hz, 1H), 3.74 (d, *J* = 5.9 Hz, 2H), 3.59 (d, *J* = 11.0 Hz, 2H), 3.36 (d, *J* = 10.9 Hz, 2H), 2.65 (q, *J* = 6.7 Hz, 1H), 2.41 – 2.25 (m, 5H), 2.18 (s, 3H), 1.99 – 1.76 (m, 4H), 1.73 – 1.66 (m, 2H).

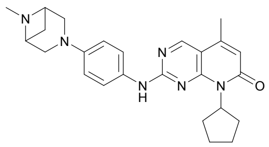

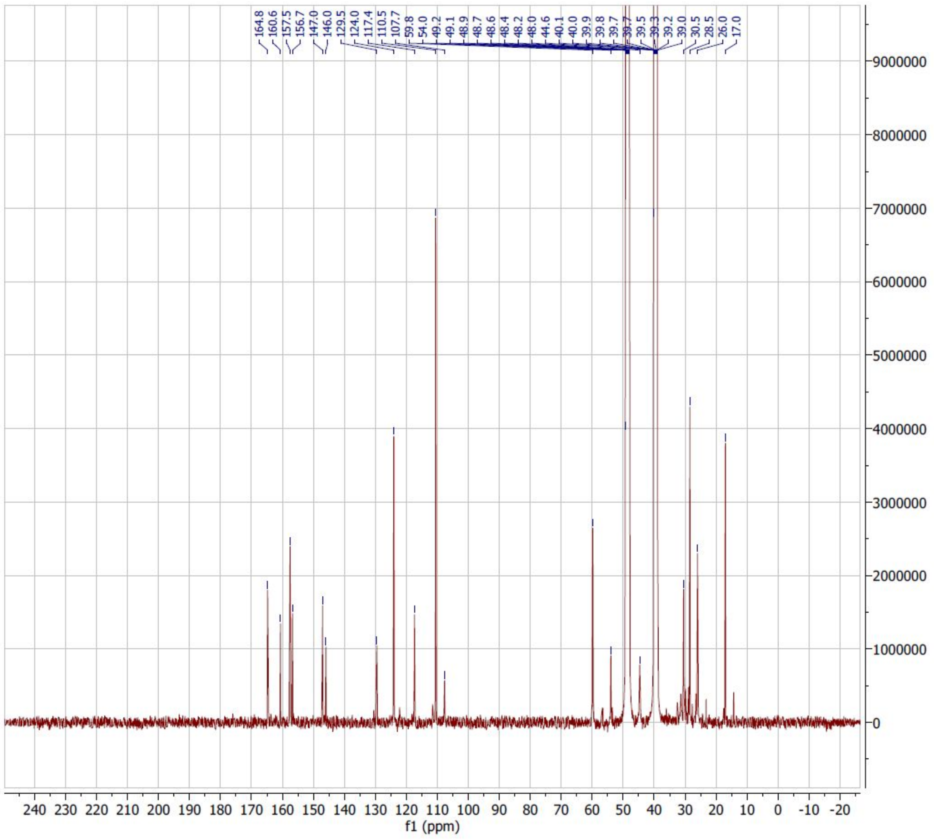

Parameter	Value
1 Title	alborada-hkb13-009-4_reproc
2 Origin	Bruker BioSpin GmbH
3 Instrument	aberlour
4 Solvent	DMSO
5 Temperature	298.0
6 Pulse Sequence	zgpg30
7 Experiment	
8 Probe	Z115434_0002 (CP DCH 500S2 C/ H-D-05 Z ET)
9 Number of Scans	4096
10 Receiver Gain	1820.0
11 Relaxation Delay	2.0000
12 Pulse Width	10.5000
13 Presaturation Frequency	
14 Spectrometer Frequency	125.74
15 Spectral Width	34722.2
16 Lowest Frequency	-3335.6
17 Nucleus	13C
18 Acquired Size	104893
19 Spectral Size	524288
20 Digital Resolution	

^{13}C NMR (126 MHz, DMSO) δ 164.8, 160.6, 157.5, 156.7, 147.0, 146.0, 129.5, 124.1, 117.4, 110.5, 107.7, 59.8, 54.0, 44.6, 30.5, 28.5, 26.0, 17.0.

Column Name ACQUITY UPLC® HSS C18 1.8µm

3: UV Detector: TAC: Wavelength Range: (230 - 400) Smooth (SG, 1x1)

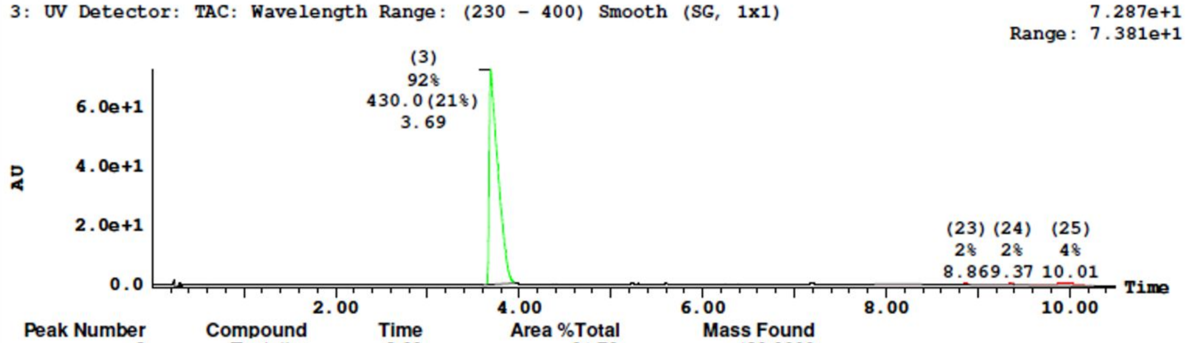

Peak Number	Compound	Time	Area %Total	Mass Found
3	Tentative	3.69	91.78	430.0000

Peak ID	Compound	Time	Mass Found
3	Tentative	3.70	431

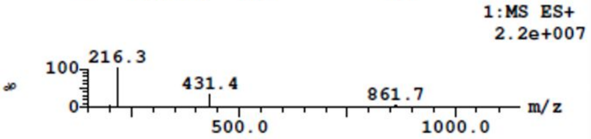

Peak ID	Compound	Time	Mass Found
3		3.70	Not Found

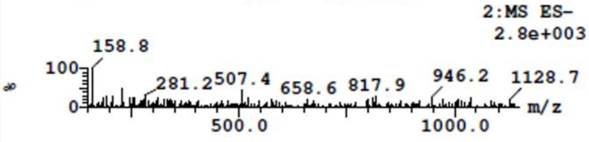

Compound 10

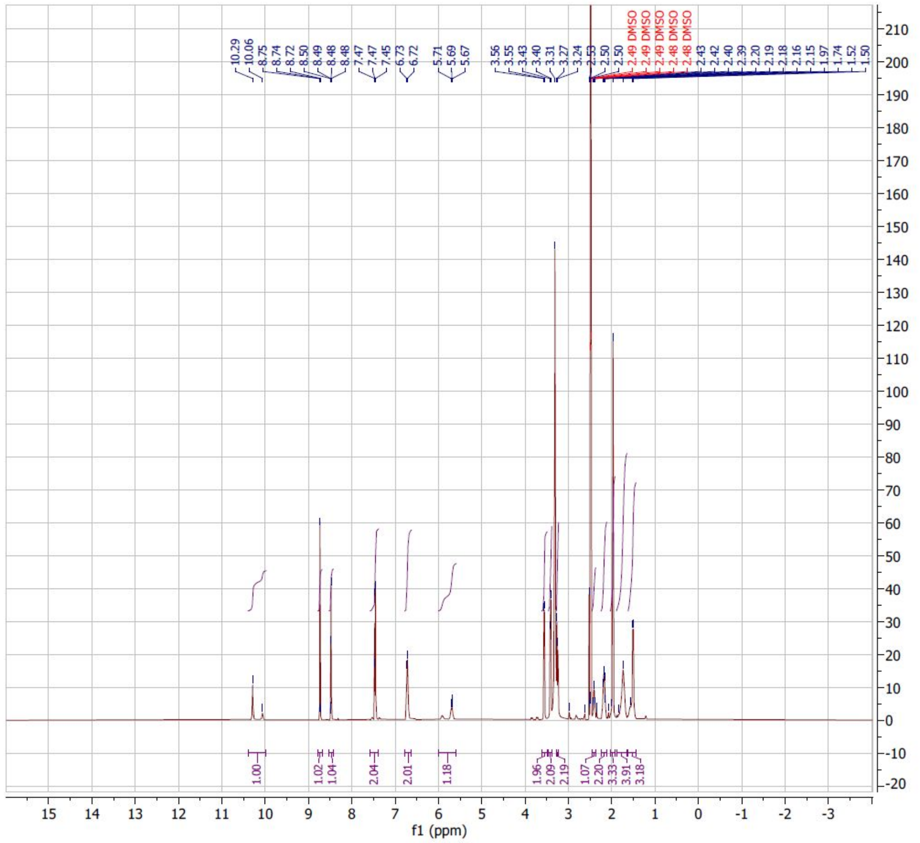

Parameter		Value
1	Title	alborada-hkb7-031-3.10.fid
2	Origin	Bruker BioSpin GmbH
3	Solvent	DMSO
4	Temperature	298.0
5	Pulse Sequence	zg
6	Experiment	1D
7	Probe	Z115434_0002 (CP DCH 500S2 C/ H-D-05 Z ET)
8	Number of Scans	1
9	Receiver Gain	18.9
10	Relaxation Delay	0.1000
11	Pulse Width	10.5000
12	Presaturation Frequency	
13	Modification Date	2024-10-03T15:01:44
14	Spectrometer Frequency	500.05
15	Spectral Width	10000.0
16	Lowest Frequency	-2010.7
17	Nucleus	1H
18	Acquired Size	32768
19	Spectral Size	65536
20	Digital Resolution	0.15

^{1}H NMR (500 MHz, DMSO) δ 10.40 – 9.98 (m, 1H), 8.74 (s, 1H), 8.48 (s, 1H), 7.46 (d, J = 8.8 Hz, 2H), 6.72 (d, J = 8.8 Hz, 2H), 6.00 – 5.59 (m, 1H), 3.56 (d, J = 5.9 Hz, 2H), 3.42 (d, J = 11.1 Hz, 2H), 3.27 – 3.23 (m, 2H), 2.41 (q, J = 7.1 Hz, 1H), 2.24 – 2.12 (m, 2H), 1.97 (s, 3H), 1.89 – 1.65 (m, 4H), 1.62 – 1.44 (m, 3H).

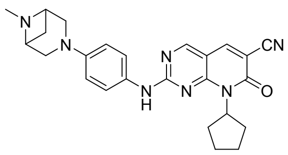

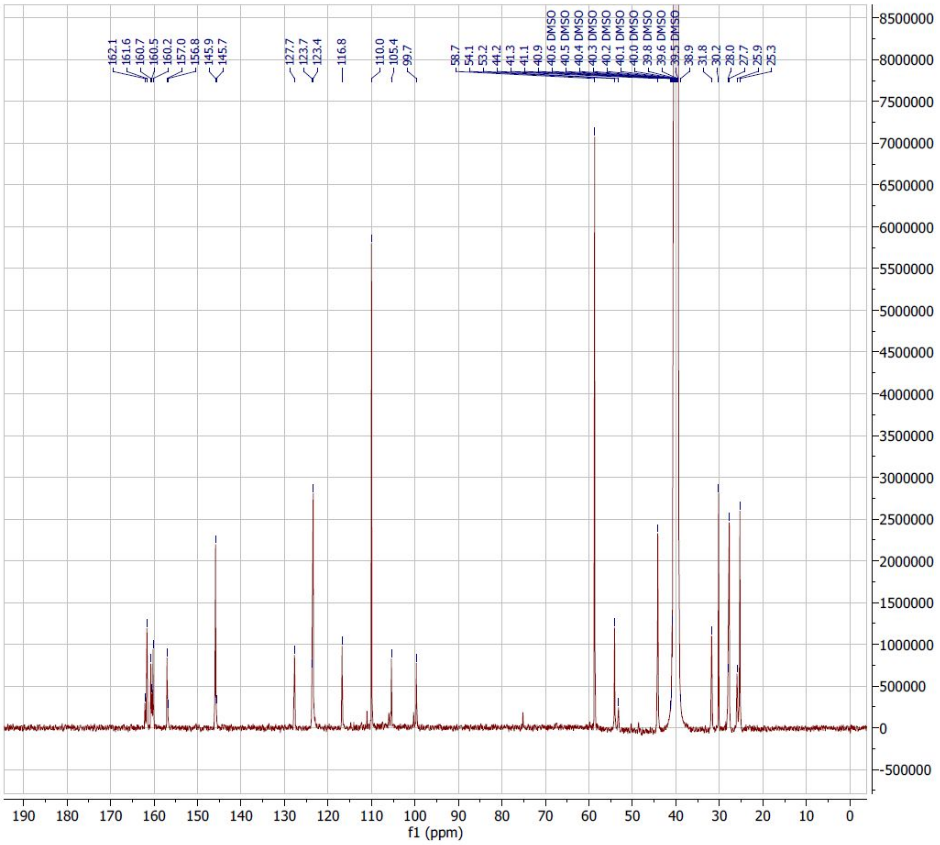

Parameter		Value
1	Title	alborada-hkb7-031-3_reproc
2	Origin	Bruker BioSpin GmbH
3	Instrument	aberlour
4	Solvent	DMSO
5	Temperature	298.0
6	Pulse Sequence	zgpg30
7	Experiment	
8	Probe	Z115434_0002 (CP DCH 500S2 C/ H-D-05 Z ET)
9	Number of Scans	4096
10	Receiver Gain	1820.0
11	Relaxation Delay	2.0000
12	Pulse Width	10.5000
13	Presaturation Frequency	
14	Spectrometer Frequency	125.74
15	Spectral Width	34722.2
16	Lowest Frequency	-3532.2
17	Nucleus	13C
18	Acquired Size	104893
19	Spectral Size	524288
20	Digital Resolution	

^{13}C NMR (126 MHz, DMSO) δ 161.64, 160.74, 160.23, 157.03, 145.87, 127.70, 123.70, 123.41, 116.76, 109.99, 105.42, 99.68, 58.68, 54.09, 44.17, 31.79, 30.19, 27.73, 25.28.

Column Name ACQUITY UPLC® HSS C18 1.8µm

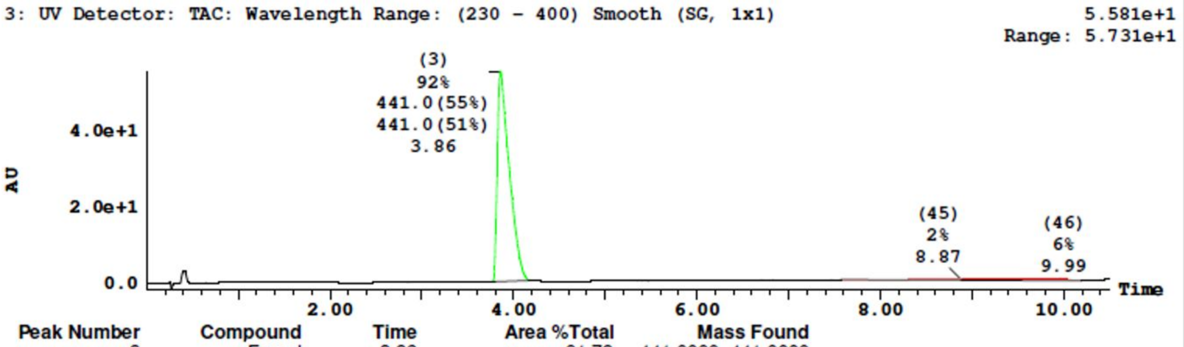

Peak Number	Compound	Time	Area %Total	Mass Found
3	Found	3.86	91.78	441.0000, 441.0000

Peak ID	Compound	Time	Mass Found
3	Found	3.86	442

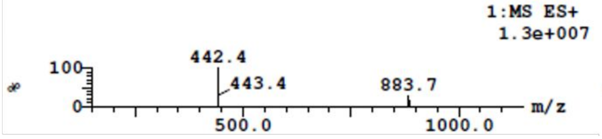

Peak ID	Compound	Time	Mass Found
3	Found	3.86	440

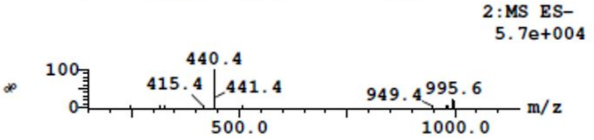

Compound 16

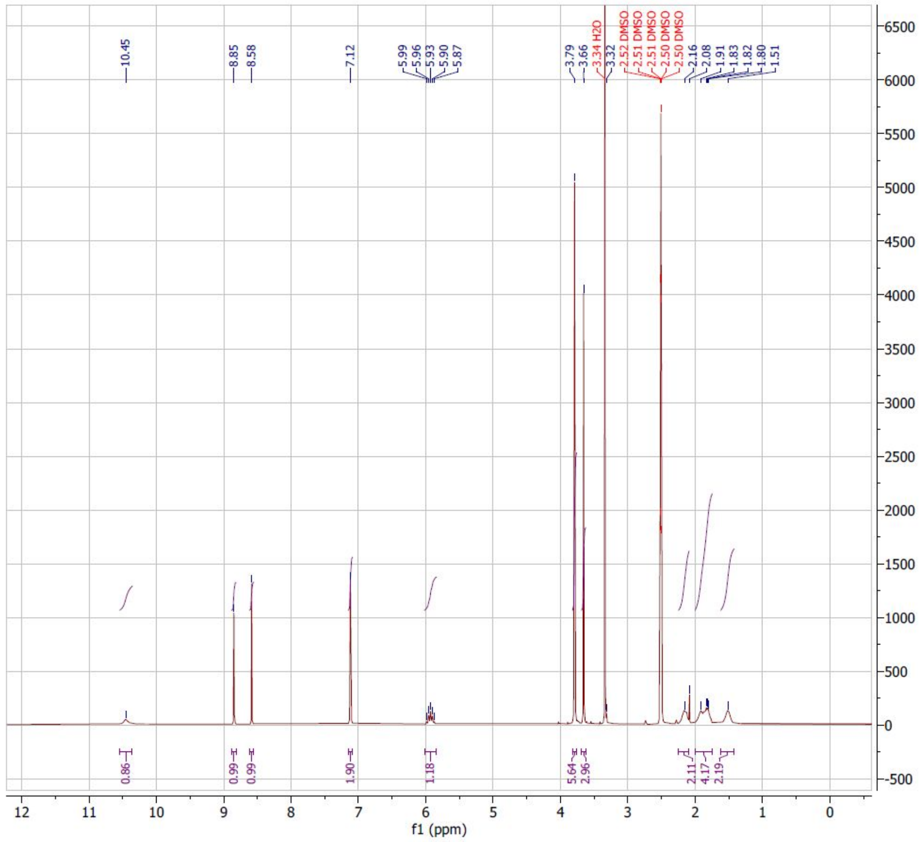

Parameter	Value
1 Title	tpcr6-051-3.10.fid
2 Origin	Bruker BioSpin GmbH
3 Solvent	DMSO
4 Temperature	295.1
5 Pulse Sequence	zg30
6 Experiment	1D
7 Probe	5 mm PABBO BB-1H/ D Z-GRD Z104275/ 0242
8 Number of Scans	64
9 Receiver Gain	191.9
10 Relaxation Delay	1.0000
11 Pulse Width	14.0000
12 Presaturation Frequency	
13 Modification Date	2019-06-18T20:03:08
14 Spectrometer Frequency	300.13
15 Spectral Width	6009.6
16 Lowest Frequency	-1151.4
17 Nucleus	1H
18 Acquired Size	32768
19 Spectral Size	65536
20 Digital Resolution	

^{1}H NMR (300 MHz, DMSO) δ 10.45 (s, 1H), 8.85 (s, 1H), 8.58 (s, 1H), 7.12 (s, 2H), 5.93 (p, J = 8.7 Hz, 1H), 3.79 (s, 6H), 3.66 (s, 3H), 2.16 (s, 2H), 2.00 – 1.75 (m, 4H), 1.51 (s, 2H).

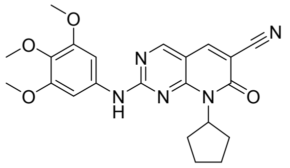

Column Name ACQUITY UPLC® HSS C18 1.8µm

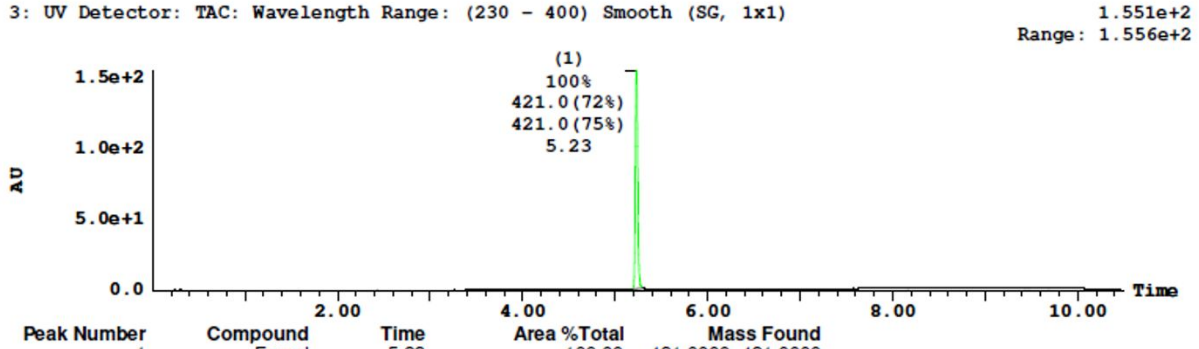

Peak Number	Compound	Time	Area %Total	Mass Found
1	Found	5.23	100.00	421.0000, 421.0000

Peak ID	Compound	Time	Mass Found
1	Found	5.23	444,422

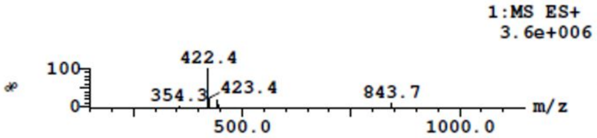

Peak ID	Compound	Time	Mass Found
1	Found	5.23	420

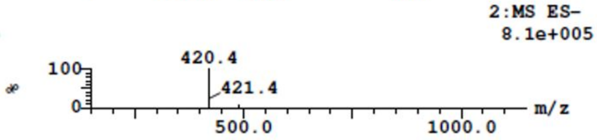

Compound 20

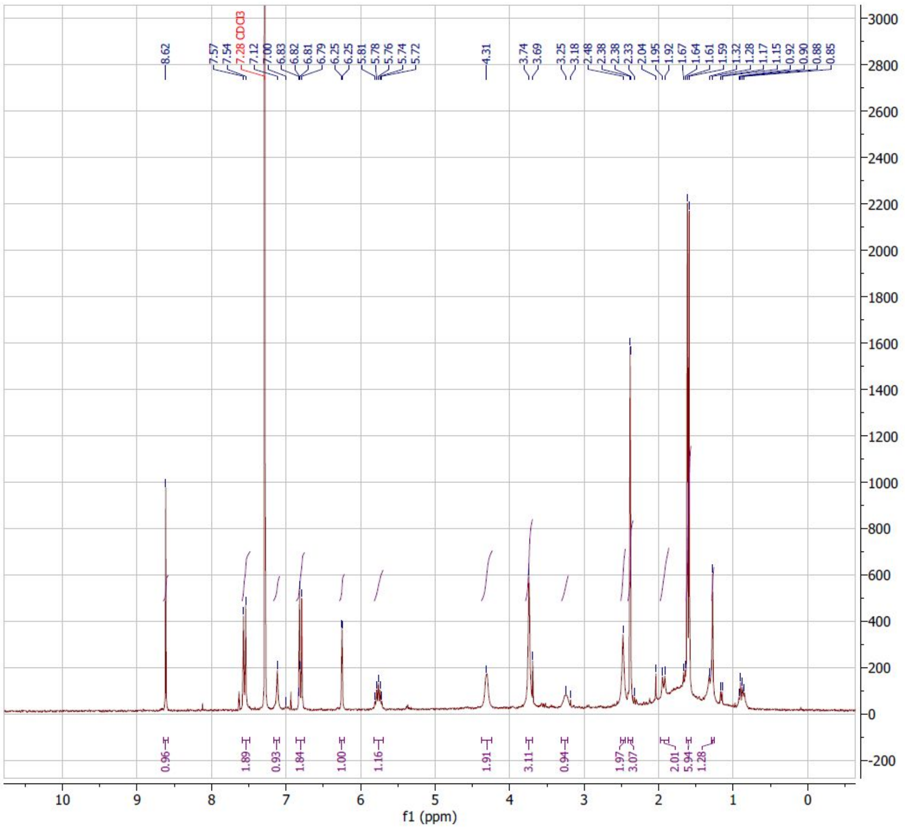

	Parameter	Value
1	Title	tpcr7-058-4.10.fid
2	Origin	Bruker BioSpin GmbH
3	Solvent	CDCl3
4	Temperature	294.1
5	Pulse Sequence	zg30
6	Experiment	1D
7	Probe	5 mm PABBO BB-1H/ D Z-GRD Z104275/ 0242
8	Number of Scans	128
9	Receiver Gain	191.9
10	Relaxation Delay	1.0000
11	Pulse Width	14.0000
12	Presaturation Frequency	
13	Modification Date	2019-12-18T13:57:52
14	Spectrometer Frequency	300.13
15	Spectral Width	6009.6
16	Lowest Frequency	-1151.4
17	Nucleus	1H
18	Acquired Size	32768
19	Spectral Size	65536
20	Digital Resolution	

^{1}H NMR (300 MHz, $CDCl_3$) δ 8.62 (s, 1H), 7.56 (d, *J* = 8.9 Hz, 2H), 7.12 (s, 1H), 6.86 – 6.75 (m, 2H), 6.25 (d, *J* = 1.3 Hz, 1H), 5.76 (p, *J* = 6.8 Hz, 1H), 4.31 (s, 2H), 3.74 (s, 3H), 3.25 (s, 1H), 2.48 (s, 2H), 2.38 (d, *J* = 1.2 Hz, 3H), 1.93 (d, *J* = 9.5 Hz, 2H), 1.60 (d, *J* = 7.0 Hz, 6H), 1.28 (s, 1H).

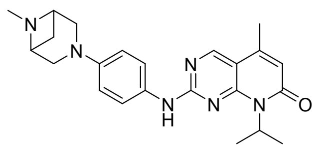

Column Name ACQUITY UPLC® HSS C18 1.8µm

3: UV Detector: TAC: Wavelength Range: (230 - 400) Smooth (SG, 1x1)

4.15e+1
Range: 4.262e+1

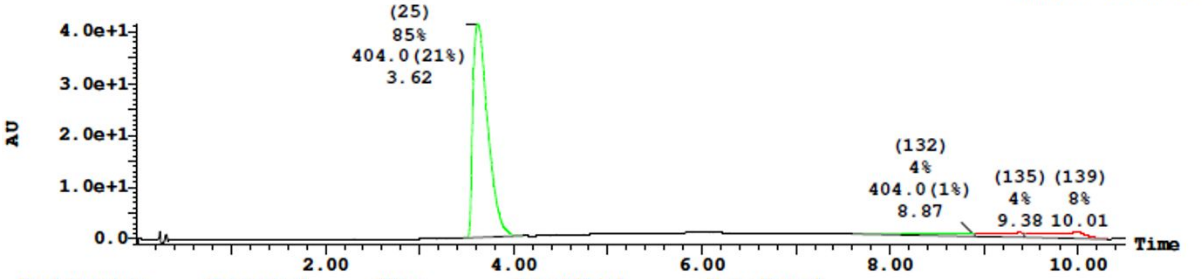

Peak Number	Compound	Time	Area %Total	Mass Found
25	Tentative	3.62	84.62	404.0000

Peak ID	Compound	Time	Mass Found
25	Tentative	3.62	405

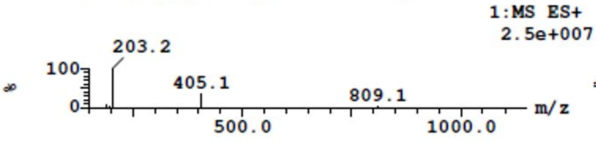

Peak ID	Compound	Time	Mass Found
25		3.62	Not Found

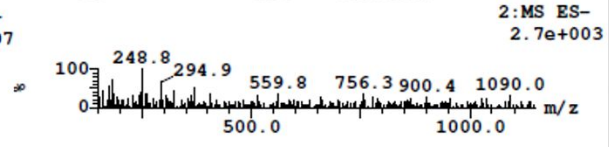

Compound 24

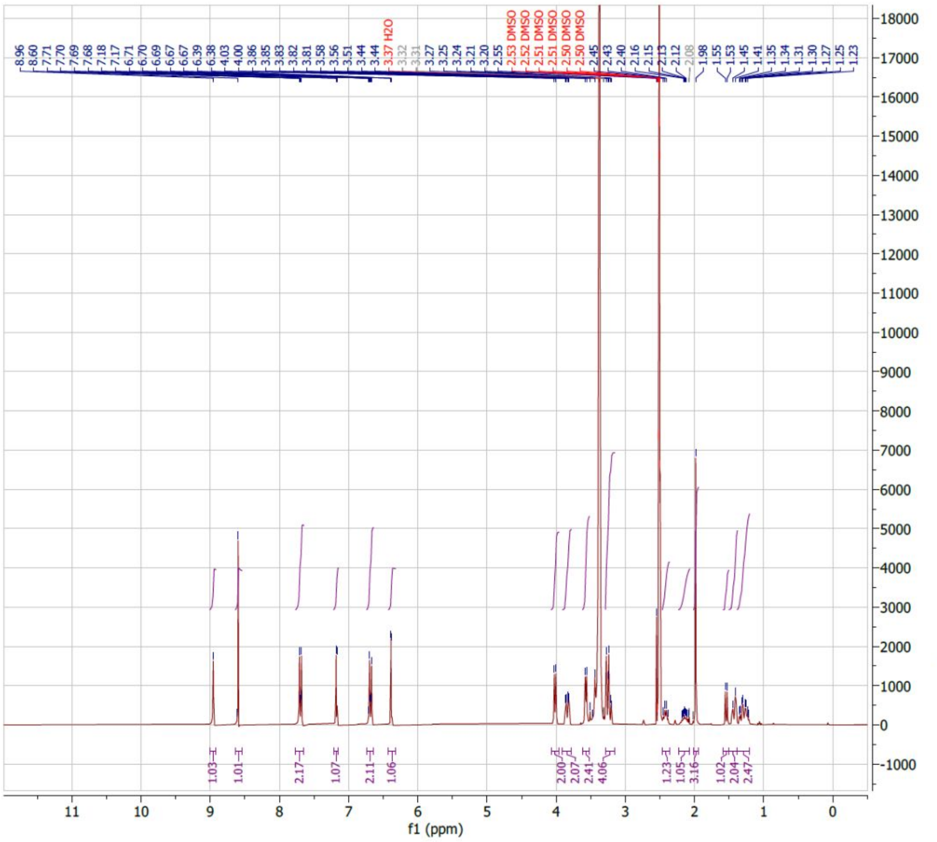

Parameter	Value
1 Title	GGA4-093-2.10.fid
2 Origin	Bruker BioSpin GmbH
3 Solvent	DMSO
4 Temperature	297.5
5 Pulse Sequence	zg30
6 Experiment	1D
7 Probe	5 mm PABBO BB-1H/ D Z-GRD Z104275/ 0242
8 Number of Scans	128
9 Receiver Gain	191.9
10 Relaxation Delay	1.0000
11 Pulse Width	14.0000
12 Presaturation Frequency	
13 Modification Date	2020-07-29T16:09:40
14 Spectrometer Frequency	300.13
15 Spectral Width	6009.6
16 Lowest Frequency	-1151.4
17 Nucleus	1H
18 Acquired Size	32768
19 Spectral Size	65536
20 Digital Resolution	

^{1}H NMR (300 MHz, DMSO) δ 8.96 (s, 1H), 8.60 (s, 1H), 7.76 – 7.65 (m, 2H), 7.17 (d, *J* = 3.5 Hz, 1H), 6.74 – 6.64 (m, 2H), 6.38 (d, *J* = 3.5 Hz, 1H), 4.01 (d, *J* = 7.1 Hz, 2H), 3.91 – 3.78 (m, 2H), 3.57 (d, *J* = 5.8 Hz, 2H), 3.24 (dd, *J* = 11.4, 9.0 Hz, 4H), 2.42 (q, *J* = 6.5 Hz, 1H), 2.23 – 2.07 (m, 1H), 1.98 (s, 3H), 1.54 (d, *J* = 8.1 Hz, 1H), 1.43 (d, *J* = 12.3 Hz, 2H), 1.28 (qd, *J* = 11.8, 5.2 Hz, 2H).

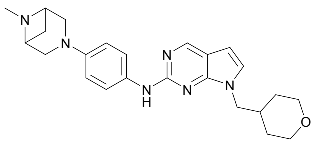

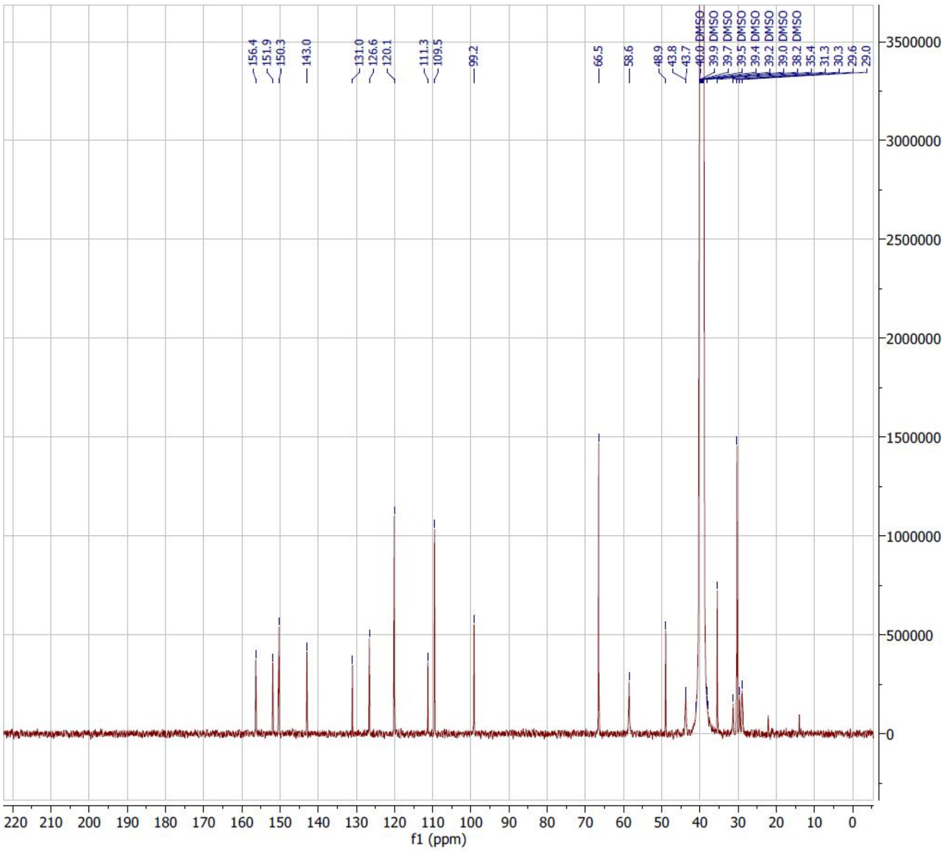

Parameter	Value
1 Title	
2 Origin	Bruker BioSpin GmbH
3 Instrument	aberlour
4 Solvent	DMSO
5 Temperature	298.0
6 Pulse Sequence	zgpg30
7 Experiment	1D
8 Probe	Z115434_0002 (CP DCH 500S2 C/ H-D-05 Z ET)
9 Number of Scans	4096
10 Receiver Gain	1820.0
11 Relaxation Delay	2.0000
12 Pulse Width	10.5000
13 Presaturation Frequency	
14 Spectrometer Frequency	125.74
15 Spectral Width	34722.2
16 Lowest Frequency	-3589.4
17 Nucleus	13C
18 Acquired Size	104893
19 Spectral Size	524288
20 Digital Resolution	0.07

^{13}C NMR (126 MHz, DMSO) δ 156.4, 151.9, 150.3, 143.0, 131.0, 126.6, 120.1, 111.3, 109.5, 99.2, 66.5, 58.6, 48.9, 43.7, 35.4, 31.3, 30.3, 29.6, 29.0.

Column Name ACQUITY UPLC® HSS C18 1.8µm

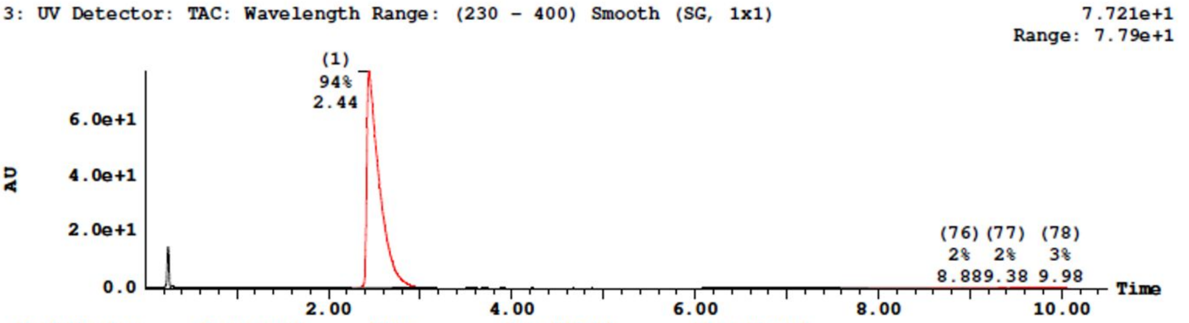

Peak Number	Compound	Time	Area %Total	Mass Found
1		2.44	94.10	Not Found

Peak ID	Compound	Time	Mass Found
1		2.45	Not Found

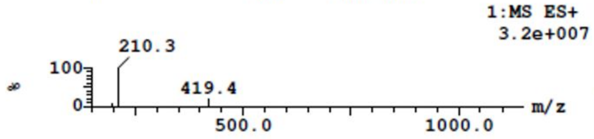

Peak ID	Compound	Time	Mass Found
1		2.45	Not Found

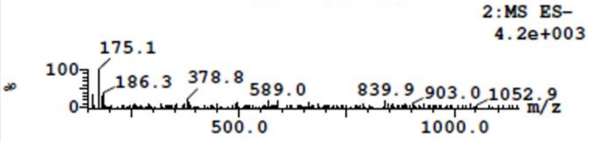

References

(1) Davis, M. I.; Sasaki, A. T.; Shen, M.; Emerling, B. M.; Thorne, N.; Michael, S.; Pragani, R.; Boxer, M.; Sumita, K.; Takeuchi, K.; Auld, D. S.; Li, Z.; Cantley, L. C.; Simeonov, A. A Homogeneous, High-Throughput Assay for Phosphatidylinositol 5-Phosphate 4- Kinase with a Novel, Rapid Substrate Preparation. PLoS One **2013**, *8*, No. e54127. https://doi.org/10.1371/journal.pone.0054127

(2) Banerjee, S.; Buhrlage, S. J.; Huang, H. T.; Deng, X.; Zhou, W.; Wang, J.; Traynor, R.; Prescott, A. R.; Alessi, D. R.; Gray, N. S. Characterization of WZ4003 and HTH-01-015 as Selective Inhibitors of the LKB1-Tumour-Suppressor-Activated NUAK Kinases. *Biochemical Journal* **2014**, *457* (1), 215–225. https://doi.org/10.1042/BJ20131152.

(3) VanderWel, S.N.; Harvey, P.J.; McNamara, D.J. Pyrido[2,3-d]pyrimidin-7-ones as specific inhibitors of cyclin-dependent kinase 4. *J Med Chem*. **2005**, *48* (7), 2371-2387. doi:10.1021/jm049355+

(4) Katamreddy, S.; Saved, Y.; Ramireddy, N. Cyclin-Dependent Kinase Inhibitors, WO2020140054 A1, 2020
